# Noncanonical WNT5A controls the activation of latent TGF-**β** to drive fibroblast activation and tissue fibrosis

**DOI:** 10.1172/JCI159884

**Published:** 2024-03-26

**Authors:** Thuong Trinh-Minh, Chih-Wei Chen, Cuong Tran Manh, Yi-Nan Li, Honglin Zhu, Xiang Zhou, Debomita Chakraborty, Yun Zhang, Simon Rauber, Clara Dees, Neng-Yu Lin, Delf Kah, Richard Gerum, Christina Bergmann, Alexander Kreuter, Christiane Reuter, Florian Groeber-Becker, Beate Eckes, Oliver Distler, Ben Fabry, Andreas Ramming, Alexandra Schambony, Georg Schett, Jörg H.W. Distler

**Affiliations:** 1 Department of Rheumatology and; 2 Hiller Research Center, University Hospital Düsseldorf, Medical Faculty of Heinrich Heine University, Düsseldorf, North-Rhine-Westphalia, Germany.; 3 Department of Internal Medicine 3 – Rheumatology and Immunology, Friedrich-Alexander-University Erlangen-Nürnberg (FAU) and University Hospital Erlangen, Erlangen, Bavaria, Germany.; 4 German Center for Immunotherapy, Friedrich-Alexander-University Erlangen-Nürnberg (FAU) and University of Erlangen, Erlangen, Bavaria, Germany.; 5 Department of Rheumatology, Xiangya Hospital, Central South University, Changsha, Hunan Province, China.; 6 Graduate Institute of Anatomy and Cell Biology, National Taiwan University College of Medicine, Taipei, Taiwan.; 7 Department of Physics, Friedrich-Alexander-University Erlangen-Nürnberg (FAU), Erlangen, Bavaria, Germany.; 8 Clinic for Dermatology, Venereology and Allergology, HELIOS St. Elisabeth Clinic Oberhausen, North-Rhine-Westphalia, Germany.; 9 Translational Center for Regenerative Therapies, Fraunhofer Institute for Silicate Research (ISC) Würzburg, Bavaria, Germany.; 10 Translational Matrix Biology, University of Cologne, Cologne, North-Rhine-Westphalia, Germany.; 11 Cologne Excellence Cluster on Cellular Stress Responses in Aging-Associated Diseases (CECAD), University of Cologne, Cologne, North-Rhine-Westphalia, Germany.; 12 Rheumaklinik, University Hospital Zurich, Zurich, Switzerland.; 13 Division of Developmental Biology, Biology Department, Friedrich-Alexander-University Erlangen-Nürnberg (FAU), Erlangen, Bavaria, Germany.

**Keywords:** Dermatology, Pulmonology, Fibrosis

## Abstract

Transforming growth factor β (TGF-β) signaling is a core pathway of fibrosis, but the molecular regulation of the activation of latent TGF-β remains incompletely understood. Here, we demonstrate a crucial role of WNT5A/JNK/ROCK signaling that rapidly coordinates the activation of latent TGF-β in fibrotic diseases. WNT5A was identified as a predominant noncanonical WNT ligand in fibrotic diseases such as systemic sclerosis, sclerodermatous chronic graft-versus-host disease, and idiopathic pulmonary fibrosis, stimulating fibroblast-to-myofibroblast transition and tissue fibrosis by activation of latent TGF-β. The activation of latent TGF-β requires rapid JNK- and ROCK-dependent cytoskeletal rearrangements and integrin α_V_ (ITGAV). Conditional ablation of WNT5A or its downstream targets prevented activation of latent TGF-β, rebalanced TGF-β signaling, and ameliorated experimental fibrosis. We thus uncovered what we believe to be a novel mechanism for the aberrant activation of latent TGF-β in fibrotic diseases and provided evidence for targeting WNT5A/JNK/ROCK signaling in fibrotic diseases as a new therapeutic approach.

## Abstract 2

WNT5A induces integrin &alpha;_V_ clustering to promote fibrosis via activation of latent TGF-&beta;; and inactivation of WNT5A signaling rebalances TGF-&beta; signaling and ameliorates experimental fibrosis.

## Graphical abstract

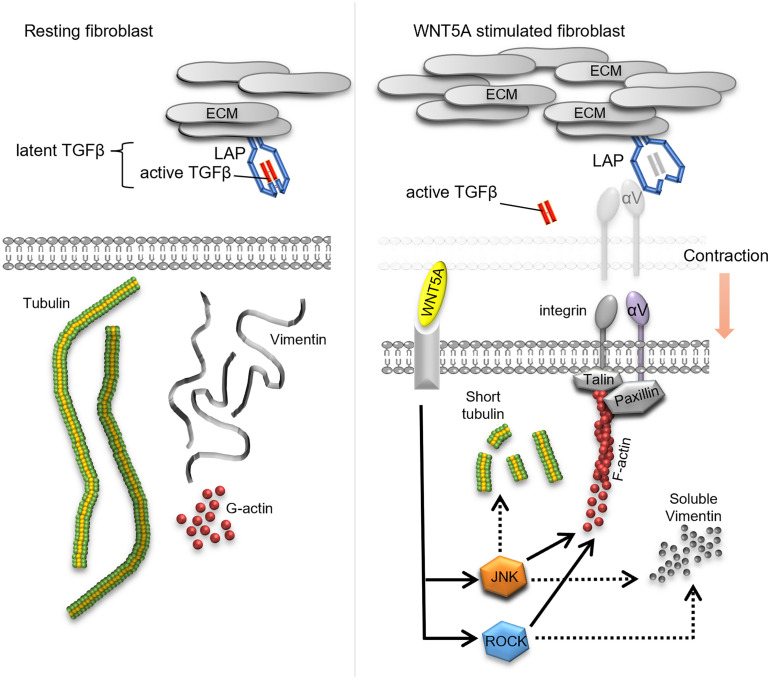

## Introduction

Fibrosis is defined as the excessive deposition of extracellular matrix in the affected tissues. Activated myofibroblasts are the principal source of extracellular matrix production in physiologic tissue repair as well as in fibrotic disease (1). However, while myofibroblast differentiation is tightly controlled and terminated in physiologic repair responses such as wound healing, fibroblasts escape such regulation in fibrotic diseases, remain persistently active, and release abundant amounts of extracellular matrix (2, 3). Fibrotic tissue remodeling imposes a major burden on modern societies; it may contribute to up to 45% of deaths in the developed world and cause socioeconomic costs in the order of tens-of-billions of dollars per year. Nonetheless, there are no effective targeted therapies for most fibrotic diseases (1, 4).

Transforming growth factor-β1 (TGF-β) has been characterized as a central profibrotic mediator (5–7). TGF-β is secreted as an inactive, latent protein complex consisting of TGF-β and noncovalently bound latency associated peptide (LAP) and requires further processing for activation (8). Abundant amounts of latent TGF-β are stored in the extracellular matrix and tight regulation of its activation is critical to avoid uncontrolled TGF-β signaling (9–11). Despite major progress in the characterization of the biochemical mechanisms of TGF-β activation (12–14), the pathomechanisms underlying the sustained activation of latent TGF-β in fibrotic diseases remain incompletely understood. Therefore, identification of key amplification molecules that trigger TGF-β activation and drive the persistent activation of fibroblasts is required to define the most promising candidate molecules for antifibrotic therapies.

Wingless/integrase-1 (WNT) proteins comprise a family of 19 secreted glycoproteins in humans with pleiotropic effects mediated by distinct intracellular signaling cascades (15–17). If the WNT-induced intracellular responses depend on β-catenin, they are referred to as canonical WNT signaling or WNT/β-catenin signaling (15, 18), whereas β-catenin–independent pathways are referred to as noncanonical WNT signaling pathways(19).

Numerous studies have characterized WNT/β-catenin signaling as a core pathway in fibrotic diseases (2, 20–22). The role of β-catenin–independent WNT pathways in the pathogenesis of fibrosis, by contrast, remains incompletely understood. Although first reports point to an upregulation of noncanonical WNT pathways during tissue remodeling, systematic studies analyzing their role in the pathogenesis of fibrotic diseases have not been carried out (23–25).

## Results

### WNT5A is upregulated in fibrotic conditions.

**Figure 1:**
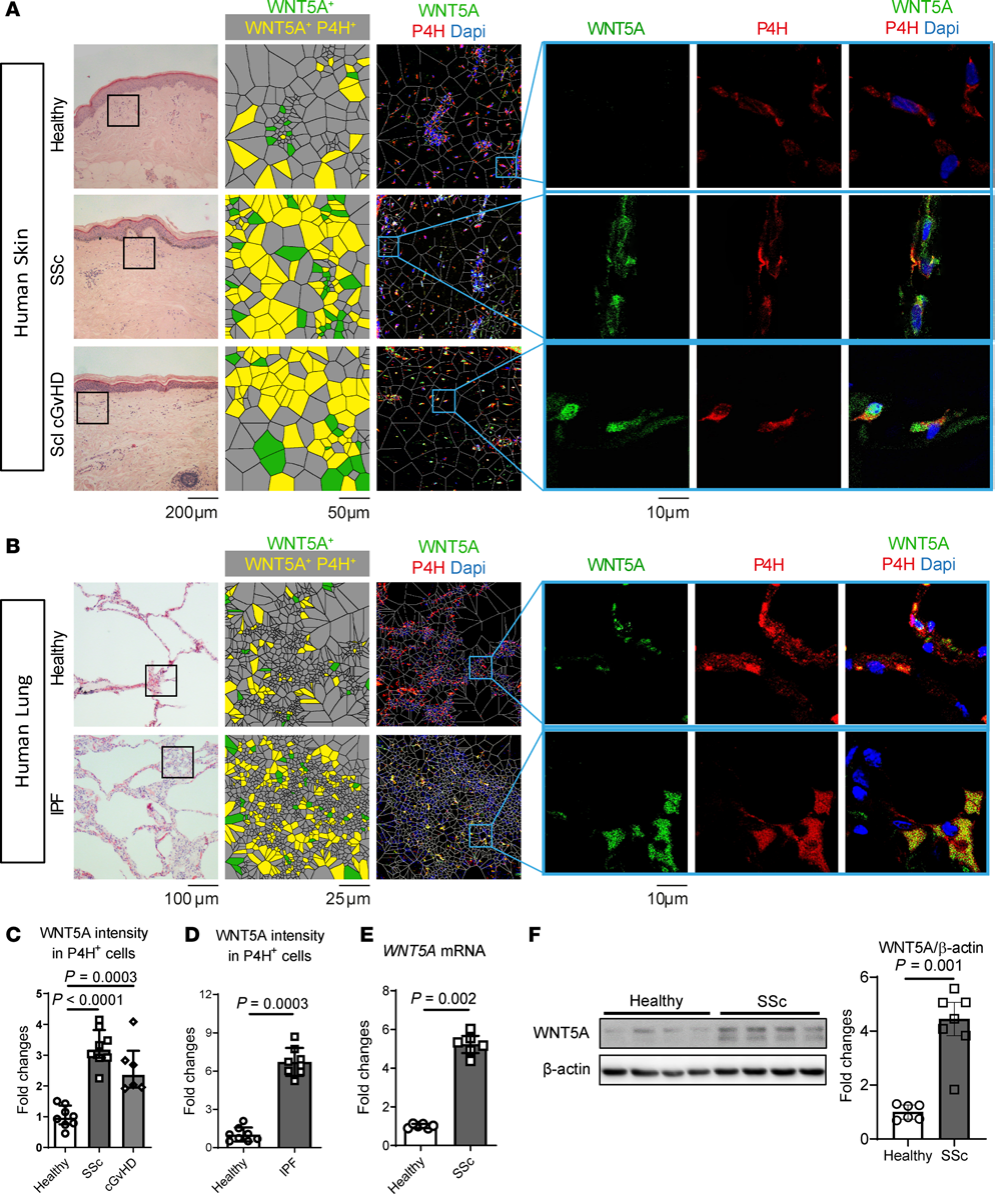
WNT5A is expressed at increased levels in human fibrotic diseases such as SSc, Scl cGvHD, and IPF. (**A**) Representative H&E staining, IF stainings for WNT5A (green) in combination with the human fibroblast marker P4H (red) and DAPI (blue) and results of Voronoi tessellation in skin sections of patients with SSc, in skin sections of patients with sclerodermatous (Scl) cGvHD (*n* = 8 for healthy and SSc patients, *n* = 6 for Scl cGvHD patients) and (**B**) in lung sections of patients with IPF (*n* = 8 for each group), all with control sections from nonfibrotic skin or lungs, respectively. (**C** and **D**) Quantification of the WNT5A staining in each fibrotic disease. (**E**) Fold changes of *WNT5A* mRNA in fibroblasts isolated from SSc skin or from healthy skin (*n* = 6 for each group). (**F**) Protein levels of WNT5A in fibroblasts (average passage 5–7) analyzed by representative Western blots and quantification (*n* = 6 for healthy fibroblasts and *n* = 7 for SSc fibroblasts). Results are shown as median ± IQR with data representing individual data points. The statistical significance was determined by 2-tailed Mann-Whitney *U* test if 2 groups were compared or 1-way ANOVA with Tukey’s multiple comparison test in case of more than 2 comparisons.

We and others have demonstrated that canonical WNT ligands, such as WNT1 and WNT10B, are overexpressed in patients with Systemic Sclerosis (SSc) and in other fibrotic diseases (26, 27). However, the expression patterns of noncanonical WNT proteins have not been systematically studied. Profiling of all known WNT proteins that are capable of activating noncanonical WNT signaling pathways (28) identified WNT5A as the ligand with the highest mRNA levels and the most pronounced upregulation in skin samples of patients with SSc compared with people in a healthy control group (Supplemental Figure 1A; supplemental material available online with this article; https://doi.org/10.1172/JCI159884DS1). The upregulation of WNT5A mRNA in skin as well as lungs of patients with SSc was confirmed in additional published cohorts by data mining (29–32) (Supplemental Figure 1B). Immunofluorescence imaging and costaining with the fibroblast marker Prolyl-4-hydroxylase-β (P4H) demonstrated that WNT5A is expressed at particularly high levels in fibroblasts in the fibrotic skin. The upregulation of WNT5A is not only restricted to SSc, but is also observed in fibroblasts of patients with sclerodermatous chronic graft-versus-host disease (scl cGvHD) and idiopathic pulmonary fibrosis (IPF) (Figure 1, A–D). The overexpression of WNT5A in SSc fibroblasts persisted even after several passages in culture, with increased mRNA and protein levels compared with cultured fibroblasts from individuals who were healthy (Figure 1, E and F). The upregulation of WNT5A in SSc and scl cGvHD was mimicked in murine models of bleomycin-induced dermal fibrosis and the LP/J (H-2^b^) → C57/Bl6 (H-2^b^) model of scl cGvHD (Supplemental Figure 1, C and D).

### WNT5A induces fibroblast-to-myofibroblast transformation and fibrosis.

**Figure 2:**
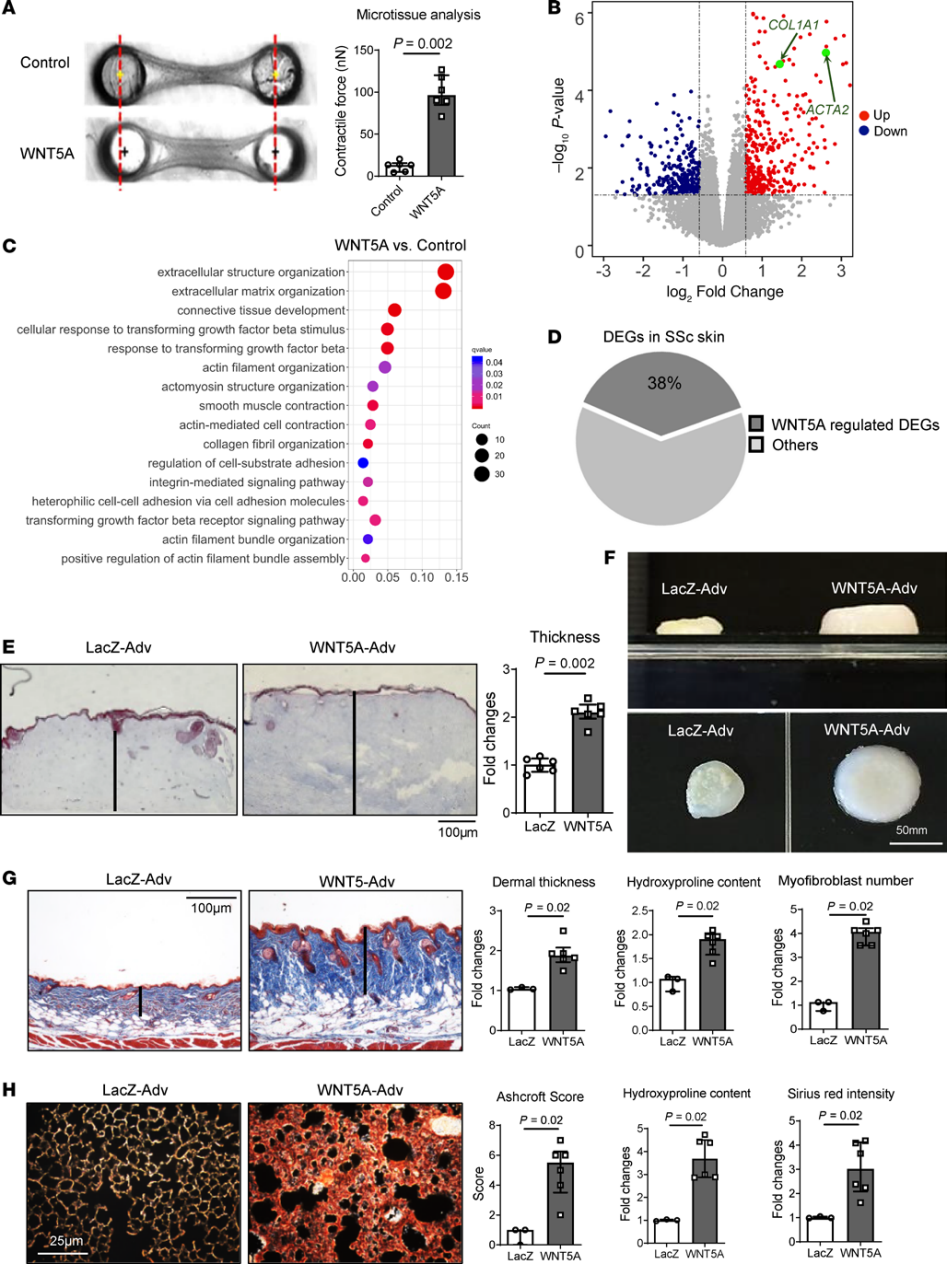
WNT5A promotes fibroblast-to-myofibroblast transition and induces dermal and pulmonary fibrosis. (**A**) Microtissue assay. Representative microtissue images and quantification of the contractile force exerted by fibroblasts (*n* = 6 for each group). (**B** and **C**) RNA-Seq of human dermal fibroblasts stimulated with WNT5A compared with control fibroblasts (*n* = 3 for each group). (**B**) Volcano plot of DEGs. The expression of each gene is plotted as the log-fold change of expression compared with controls; the 1.5-fold change threshold is marked by dotted lines. (**C**) Bubble plots displaying significant enrichment of GO biological processes. The color of the bubble represents the *q* value, and the size of the bubble represents the number of DEGs in the data sets associated with the GO processes. (**D**) Pie chart showing the percentage of DEGs in SSc skin (29) that overlap with WNT5A target genes in fibroblasts. (**E** and **F**) Full-thickness skin organoids. (**E**) Trichrome stainings and quantification, and (**F**) representative microscopic images of dermal thickness in skin organoids (*n* = 6 for each group). (**G**) Forced overexpression of Wnt5a in the skin of mice. Representative Trichrome stainings, quantification of the dermal thickness, the collagen content, and myofibroblast counts (*n* = 3 for LacZ-Adv group and *n* = 6 for WNT5A-Adv group). (**H**) Forced overexpression of Wnt5a in murine lungs. Representative sirius red stainings, ashcroft scores, hydroxyproline content, and quantifications of sirius red staining (*n* = 3 for LacZ-Adv group and *n* = 6 for WNT5A-Adv group). Results are shown as median ± IQR with data representing individual data points. The statistical significance was determined by 2-tailed Mann-Whitney *U* test. Adv, Adenovirus.

Recombinant WNT5A stimulated the expression of myofibroblast markers in resting fibroblasts, as shown by increased expression of α-SMA and induction of stress fibers (Supplemental Figure 2A). Moreover, WNT5A upregulated the mRNA levels of *COL1A1* and increased the secretion of collagen protein in conventional 2D culture conditions (Supplemental Figure 2, B and C). Stimulation with WNT5A also promoted myofibroblast-induced contraction in 3D microtissue assays (Figure 2A).

To further characterize the effects of WNT5A on fibroblasts, we performed RNA-Seq. We identified 629 differentially expressed genes (DEGs) in WNT5A-stimulated human dermal fibroblasts compared with controls (Figure 2B, Supplemental Figure 2D, and Supplemental Table 4). Using gene enrichment analysis for Gene Ontology (GO) biological processes (Figure 2C) and Gene Set Enrichment Analysis (GSEA) (Supplemental Figure 2E), we found substantial enrichment of gene sets relevant for fibroblast-to-myofibroblast differentiation. Comparison of the lists of genes regulated by WNT5A in dermal fibroblasts with genes deregulated in the skin of patients with SSc revealed that 38% of all DEGs in skin biopsies of a cohort of patients with early, diffuse-cutaneous SSc (PRESS cohort) (29) matched the genes regulated by WNT5A in cultured fibroblasts (Figure 2D). To confirm that WNT5A regulates target genes that differ from those modulated by canonical WNT signaling, we performed RNA-Seq of fibroblasts stimulated with WNT3A. The data highlight that WNT3A and WNT5A induce distinct transcriptional changes (Supplemental Figure 3, A–C). The DEGs of fibroblasts stimulated with WNT5A and WNT3A share very limited similarity, with only 19 shared DEGs out of 629 and 736 DEGs (less than 3% overlap), respectively (Supplemental Figure 3D). Although both WNT proteins are capable of activating MAPK/RAC/JNK signaling, we did not observe key functional terms of WNT5A — such as integrin- or focal adhesion-related terms — in WNT3A-stimulated fibroblasts (Supplemental Figure 3E). Moreover, noncanonical WNT ligands such as WNT10B or WNT3A did not induce the expression of WNT5A in cultured human or murine fibroblasts (Supplemental Figure 3, F and G), demonstrating that the upregulation of WNT5A is not a consequence of activated WNT/β-catenin signaling in SSc.

As the spatial organization of fibroblasts in tissues can modulate their response to specific stimuli, we further evaluated the role of WNT5A in a full-thickness model of human skin (33). Similar to recombinant WNT5A, fibroblasts infected with WNT5A-expressing adenovirus (Adv) at a multiplicity of infection (MOI) of 80 exhibited an upregulation of collagen type I and α-SMA (Supplemental Figure 4A). Comparable results were obtained with an alternative WNT5A-expressing Adv that used mCherry as a reporter (data not shown). Skin organoids engineered with fibroblasts overexpressing WNT5A demonstrated increased *ACTA2* mRNA levels, higher myofibroblast counts, higher *COL1A1* mRNA levels, and increased release of human type I collagen, compared with organoids engineered with control fibroblasts expressing LacZ (Supplemental Figure 2, F and G). The accumulation of collagen resulted in thickening of the dermal parts of WNT5A-overexpressing organoids, as in the fibrotic skin (Figure 2, E and F).

Based on the stimulatory effects of WNT5A on fibroblasts in different in vitro conditions, we next investigated whether overexpression of WNT5A was sufficient to induce fibrosis in vivo. Overexpression of WNT5A in the skin of mice by intradermal injection of WNT5A-Adv induced prominently increased WNT5A expression (Supplemental Figure 4B), dermal fibrosis with dermal thickening, accumulation of myofibroblasts, and increased hydroxyproline content compared with control mice infected with LacZ-Adv within 12 weeks (Figure 2G). Prominent fibrosis was also observed in the lungs of mice upon local overexpression of WNT5A. Intratracheal instillation of WNT5A-Adv resulted in increased Ashcroft scores, higher myofibroblast counts, enhanced collagen accumulation, and elevated hydroxyproline levels compared with mice infected with LacZ-Adv (Figure 2H).

### WNT5A-induced fibroblast activation depends on JNK and ROCK signaling.

**Figure 3:**
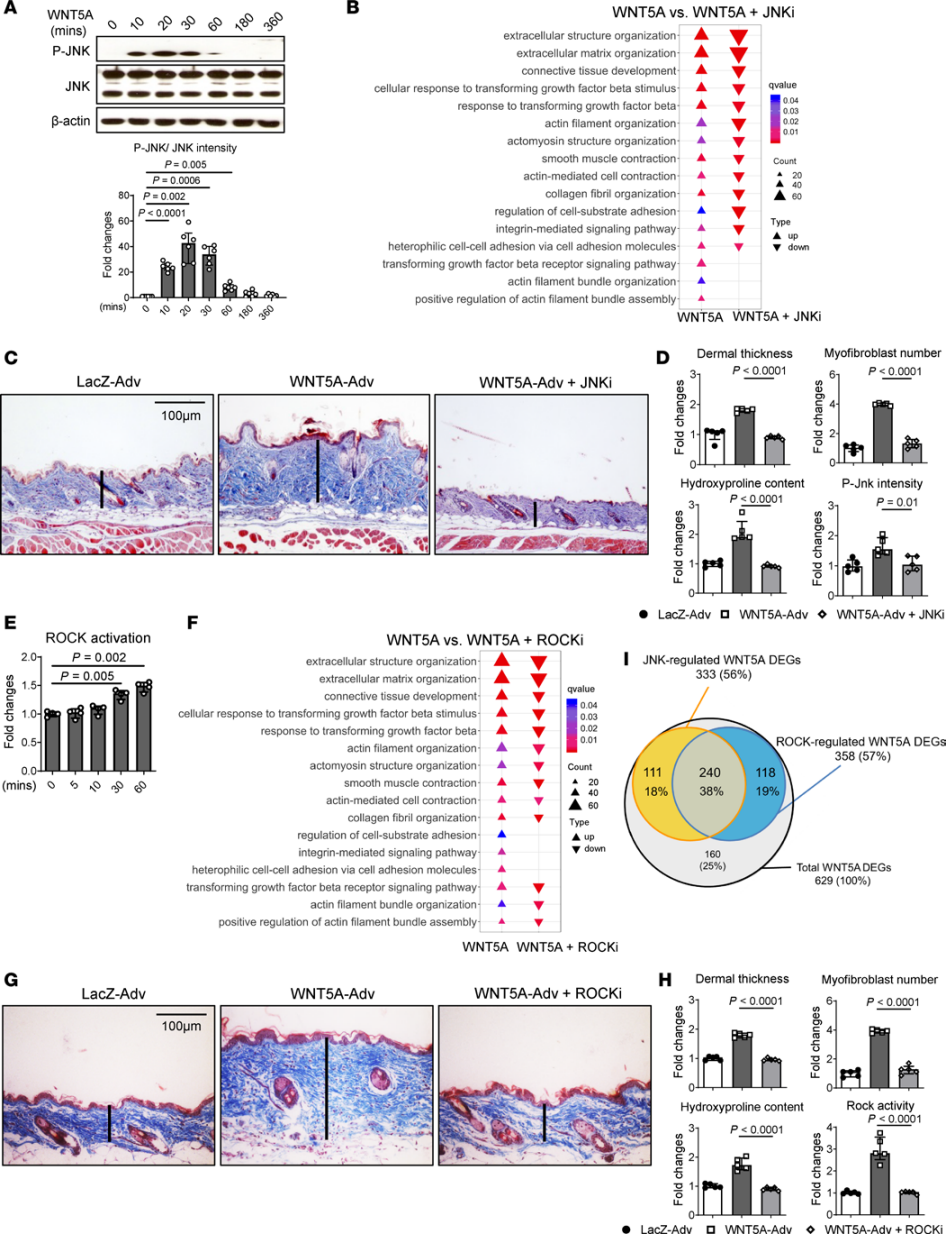
WNT5A-induced fibrosis requires JNK and ROCK. (**A-D**) JNK signaling. (**A**) Representative Western blot and quantification of pJNK in WNT5A stimulated human dermal fibroblasts (*n* = 6 for each group). (**B**) Negative enrichment scores (deenrichment) of GO biological processes related to fibroblast-to-myofibroblast transition and fibrosis in WNT5A-stimulated fibroblasts. (**C** and **D**) Effects of Jnk inhibition on Wnt5a-induced skin fibrosis in mice. (**C**) Representative trichrome stainings. (**D**) Quantification of the dermal thickness, the collagen content, myofibroblast counts, and P-Jnk immunofluorescence staining levels in tissue sections (*n* = 5 for each group). (**E**–**H**) ROCK signaling. (**E**) Quantification of ROCK activity in WNT5A-stimulated human dermal fibroblasts as analyzed by ROCK activity assays (*n* = 4 for each group). (**F**) Negative enrichment scores (deenrichment) of GO biological processes related to fibroblast-to-myofibroblast transition and fibrosis in WNT5A-stimulated fibroblasts treated with ROCKi compared with vehicle-treated WNT5A-stimulated fibroblasts (*n* = 3 for each group). (**G** and **H**) Effects of Rock inhibition on Wnt5a-induced skin fibrosis in mice. (**G**) Representative trichrome stainings. (**H**) Quantification of the dermal thickness, the collagen content, myofibroblast counts, and the Rock activity measured in tissue lysates (*n* = 5 for each group). (**I**) Venn diagram showing the number and percentage of JNK- and ROCK-regulated DEGs among all WNT5A-regulated DEGs. Results are shown as median ± IQR with data representing individual data points. The statistical significance was determined 1-way ANOVA with Tukey’s multiple comparison test. Adv, Adenovirus.

Depending on the cellular context and the target tissue, WNT5A can exert its cellular effects by activation of different intracellular cascades including JNK and ROCK signaling. In addition, WNT5A can also modulate WNT/β-catenin signaling, as well as regulate calcium (Ca^2+^) signaling via activation of the Ca^2+^/calmodulin-dependent protein kinase II (CaMKII) (34–39). Recombinant WNT5A induces phosphorylation of JNK in cultured fibroblasts (Figure 3A) with a concomitant increase in the levels of phosphorylated c-JUN, a JNK substrate (Supplemental Figure 5A). Overexpression of WNT5A in the skin or lungs of mice also induced activation of Jnk signaling in vivo, and fibroblasts expressing WNT5A strongly stained for P-Jnk and P-c-Jun (Supplemental Figure 5, B–E). Preincubation of fibroblasts with the JNK inhibitor SP600125 (JNKi) (Supplemental Figure 6, A and B) or siRNA-mediated knockdown of JNK1/2 (*JNK* siRNA) reduced the stimulatory effects of WNT5A on fibroblasts (Supplemental Figure 6, C and D). RNA-Seq demonstrated significant downregulation of WNT5A-induced profibrotic genes and fibrosis-relevant GO- (Figure 3B and Supplemental Figure 6, E and F) and GSEA-gene sets in fibroblasts incubated with WNT5A plus JNKi compared with WNT5A-stimulated fibroblasts (Supplemental Figure 6G). Moreover, treatment with the selective JNKi CC-930 ameliorated WNT5A-induced skin fibrosis (Figure 3, C and D).

WNT5A also stimulated ROCK kinase activity in cultured fibroblasts (Figure 3E) and in the skin and lung tissue of mice (Supplemental Figure 5, F and G). Incubation with the ROCK inhibitor Y27632 (ROCKi) (Supplemental Figure 7, A and B) or siRNA-mediated knockdown of the ROCK upstream kinase RHOA (*RHOA* siRNA) inhibited WNT5A-induced fibroblast activation (Supplemental Figure 7, C and D). RNA-Seq further confirmed the inhibition of the profibrotic effects of WNT5A by targeting ROCK (Figure 3F and Supplemental Figure 7, E–G). Consistently, treatment of mice with the ROCKi ameliorated WNT5A-induced skin fibrosis (Figure 3, G and H).

Further analyses of the RNA-Seq results of WNT5A-stimulated human dermal fibroblasts incubated with JNKi or ROCKi demonstrated that 75% of all WNT5A-regulated DEGs in dermal fibroblasts were regulated in a JNK- or ROCK-dependent manner. Additionally, 38% of the DEGs were regulated by both kinases, indicating synergistic effects of JNK and ROCK, whereas the remaining 37% were regulated exclusively by either JNK or ROCK (Figure 3I). Altogether, the data confirm that activation of JNK- and ROCK-signaling was critically required for the profibrotic effects of WNT5A in dermal fibroblasts.

In contrast to JNK and ROCK signaling, incubation of human dermal fibroblasts with WNT5A did not induce phosphorylation of CaMKII, a common readout for WNT/Ca^2+^ signaling (Supplemental Figure 8A). Moreover, coincubation with the intracellular calcium chelator BAPTA/AM or the Ca^2+^-ionophore calcimycin did not modulate WNT5A-induced collagen release (Supplemental Figure 8B).

Stimulation of cultured human dermal fibroblasts with WNT5A also did not activate WNT/β-catenin signaling. In contrast to the classical canonical ligand WNT1, profibrotic concentrations of WNT5A did not induce nuclear accumulation of β-catenin, stimulate β-catenin/TCF-dependent reporter activity, or induce the expression of the prototypical WNT/β-catenin target gene *AXIN-2* in fibroblasts (Supplemental Figure 8, C–F). Moreover, overexpression of WNT5A did not significantly alter the levels of nuclear β-catenin in the dermal tissues of mice (Supplemental Figure 8G).

### WNT5A induces activation of latent TGF-β.

The potent profibrotic effects of WNT5A with activation of JNK and ROCK signaling lead us to hypothesize that WNT5A may promote the activation of other key profibrotic pathways. The terms “cellular response to TGF-β”, “response to TGF-β,” and “TGF-β receptor signaling pathway” were among the top regulated GO terms induced by WNT5A in dermal fibroblasts (Figure 2C), indicating that TGF-β might be a potential downstream mediator of WNT5A. Moreover, ingenuity pathway analysis (IPA) predicted activation of TGF-β upon WNT5A stimulation.

To validate these predictions, we first compared the transcriptomes of WNT5A- and TGF-β–stimulated human dermal fibroblasts. RNA-Seq revealed significant overlap in DEGs between WNT5A- and TGF-β–stimulated fibroblasts (Supplemental Figure 9A), with the regulation of the same fibrosis-relevant GO- (Supplemental Figure 9B) and GSEA-gene sets (Supplemental Figure 9C) by TGF-β and WNT5A.

**Figure 4:**
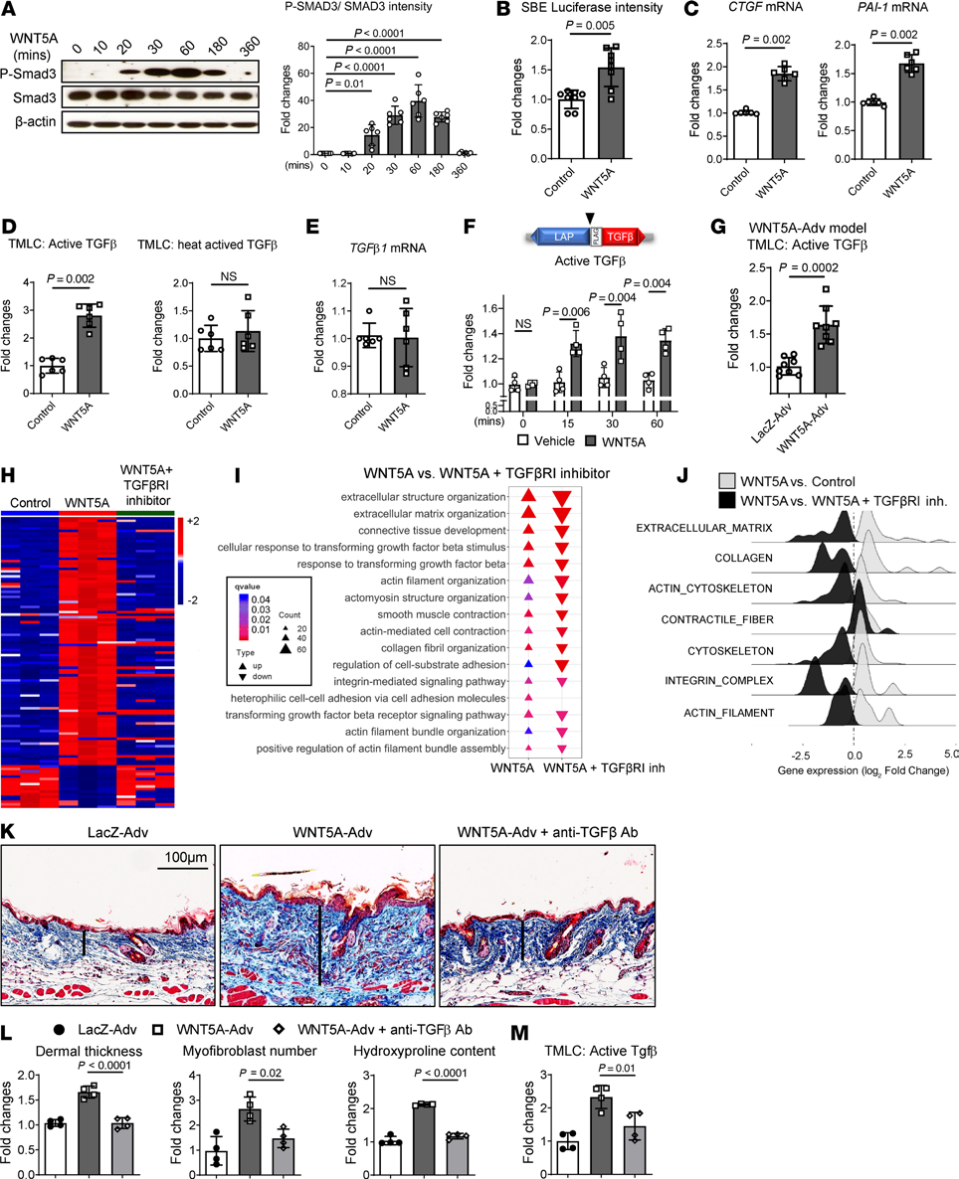
WNT5A induces activation of latent TGF-β in vitro and in vivo. (**A**) Representative Western blots and quantification of the levels of P-SMAD3 in dermal fibroblasts stimulated with WNT5A (*n* = 6 for each group). (**B**) Changes in the activity of a SBE-reporter construct (*n* = 8 for each group). (**C**) mRNA levels of the *CTGF* and *PAI-1* genes (*n* = 6 for each group). (**D**) Levels of active TGF-β and of total, heat-activated TGF-β in the supernatant of WNT5A-stimulated fibroblasts as measured by TMLC assays (*n* = 6 for each group). (**E**) Absence of changes in TGF-β1 mRNA (*n* = 6 for each group). (**F**) Quantification of active TGF-β1 in supernatants from fibroblasts expressing LAP-TGF-β1 with a cleavage exposed FLAG-tag (▼) between the LAP and the TGF-β1 coding region (*n* = 4 for each group). (**G**) Active TGF-β in the skin of mice overexpressing WNT5A (*n* = 8 for each group). (**H**) Heatmap illustration of DEGs in WNT5A-stimulated fibroblasts with or without-βRIi (*n* = 3 for each group). (**I**) Negative enrichment scores for GO biological processes related to fibroblast-to-myofibroblast transition and fibrosis. (**J**) Ridgeline plot highlighting the deenrichment of GSEA-gene sets related to fibroblast-to-myofibroblast transition and tissue fibrosis in WNT5A-stimulated fibroblasts treated with or without TGF-βRIi. (**K**) Representative trichrome stainings and (**L**) quantification of dermal thickness, hydroxyproline content, myofibroblast counts, and (**M**) active TGF-β in skin lysate from mice with WNT5A-induced fibrosis with or without TGF-βRIi (*n* = 4 for each group). Results are shown as median ± IQR. The statistical significance was determined by 2-tailed Mann-Whitney *U*-test if 2 groups were compared or 1-way ANOVA with Tukey’s multiple comparison test in figure **A**, **L**, and **M**, or 2-way ANOVA in figure **F**. Adv, Adenovirus.

Moreover, incubation of fibroblasts with WNT5A induced phosphorylation of SMAD3, a central readout for TGF-β signaling (40) (Figure 4A). WNT5A also stimulated SMAD-dependent reporter activity and upregulated the expression of the prototypical TGF-β/SMAD target genes *PAI-1* and *CTGF* in fibroblasts (Figure 4, B and C).

WNT5A/JNK/ROCK signaling may activate TGF-β/SMAD signaling at different levels: (a) upon binding of WNT5A, the WNT coreceptor RYK may associate with TGF-β receptors to promote their activation (41); (b) JNK is capable of directly phosphorylating SMAD proteins under certain conditions (42), as is ROCK; (c) WNT5A induces the synthesis of TGF-β precursor molecules at the level of transcription or translation (43, 44); (d) WNT5A might stimulate the activation of latent TGF-β. To test the first 3 possibilities, we inhibited TGF-β signaling at different levels: We co-incubated WNT5A-stimulated fibroblasts with the JNKi, the TGF-β receptor I kinase inhibitor SD-208 (TGF-βRIi), and neutralizing antibodies against TGF-β (anti-TGF-β Ab). Incubation with JNKi or siRNA-mediated knockdown of JNK prevented the upregulation of P-SMAD3 as well as the induction of *CTGF*, *PAI-1,* and also of *COL1A1* by WNT5A (Supplemental Figure 9, D–F). ROCK inhibition also blocked the WNT5A-induced accumulation of P-SMAD3 and transcription of its target genes (Supplemental Figure 9, G–I). Pretreatment with the TGF-βRIi abrogated WNT5A-induced phosphorylation of SMAD3, but did not affect the WNT5A-induced activation of JNK (Supplemental Figure 10A), demonstrating that TGF-β receptor I kinase activity is essential for WNT5A-induced phosphorylation of SMAD3 and providing evidence against a JNK-induced cross-phosphorylation of SMAD3. Furthermore, pretreatment with anti-TGF-β Ab blocked the WNT5A-induced phosphorylation of SMAD3 without affecting the WNT5A-induced activation of JNK (Supplemental Figure 10B). Pretreatment with TGF-βRIi or anti-TGF-β Ab also did not affect WNT5A-induced ROCK activation (Supplemental Figure 10C). These findings suggest that WNT5A regulates TGF-β signaling at the level of TGF-β synthesis or activation. To further differentiate between those possibilities, we analyzed changes in the levels of active TGF-β, total TGF-β protein, and *TGF*-*β1* mRNA. Stimulation of resting fibroblasts with WNT5A induced a rapid increase of active TGF-β in the cell culture supernatants, as analyzed by transformed mink lung cell (TMLC) assays (45). Stimulation with WNT5A increased levels of active TGF-β without changing the levels of total TGF-β protein or of TGF-β1 mRNA (Figure 4, D and E). The activation of latent TGF-β by WNT5A in fibroblasts was further confirmed by using activation-specific reporter constructs (46), encoding for a LAP/FLAG-TGF-β1 construct that exposes FLAG only after cleavage (Figure 4F). WNT5A also increased the levels of active TGF-β in the lysate of murine skin with forced overexpression of WNT5A (Figure 4G). Together, these data provide evidence that WNT5A promotes activation of latent TGF-β.

### Inhibition of TGF-β abrogates the profibrotic effects of WNT5A.

To evaluate the functional relevance of TGF-β for WNT5A-induced fibroblast-to-myofibroblast transition, WNT5A-stimulated human dermal fibroblasts were coincubated with TGF-βRIi or anti-TGF-β Ab. TGF-βRIi and anti-TGF-β Ab both prevented WNT5A-induced fibroblast-to-myofibroblast transdifferentiation and collagen release (Supplemental Figure 10, D and E). RNA-Seq further demonstrated that inhibition of TGF-β signaling abrogated WNT5A-induced fibroblast activation (Figure 4H and Supplemental Figure 10F) with deenrichment of WNT5A-regulated fibrosis-relevant GO (Figure 4I) and GSEA gene sets (Figure 4J). Moreover, treatment with anti-TGF-β Ab prevented Wnt5a-induced skin fibrosis (Figure 4, K–M).

### WNT5A induces integrin α_V_ clustering to promote fibrosis.

**Figure 5:**
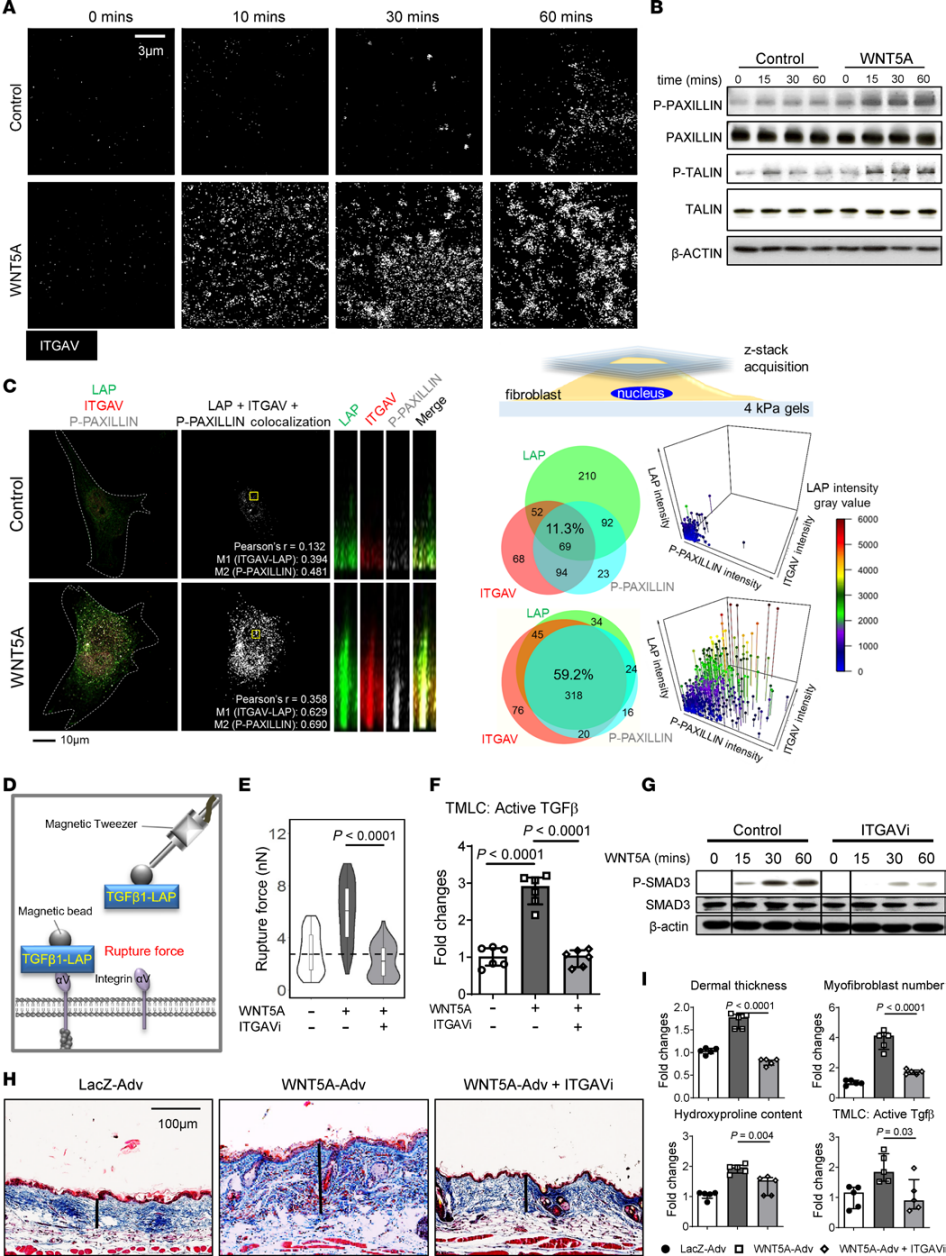
WNT5A -induced activation of latent TGF-β requires Integrin αV. (**A**) Representative confocal IF images of ITGAV clusters in dermal fibroblasts incubated with or without WNT5A (*n* = 4 for each group). (**B**) Representative Western blots of P-PAXILLIN, PAXILLIN, P-TALIN and TALIN (*n* = 3 for each group). (**C**) Representative confocal images, z-stack reconstructions, and Venn diagrams showing colocalization of LAP with ITGAV and P-PAXILLIN, and 3 -dimensional scatter plots showing the distribution of each marker at each localization (*n* ≥ 10 for each group); schematic overview of the experimental conditions and areas of assessment. Pearson’s *r* is the Pearson’s correlation coefficient between LAP-ITGAV and P-PAXILLIN voxel intensities. M1 and M2 representing for Manders’ split coefficients. (**D**) Schematic illustration of the experiment for measuring the rupture force with magnetic tweezers. (**E**) Violin plots showing the force required to rupture magnetic beads coupled with peptides containing the RGD domain of LAP-TGF-β1 (R**RGD**LATISPASSKGGGGSRLLLLLLR) from the cell surface of dermal fibroblasts incubated with WNT5A with or without ITGAV inhibitor (ITGAVi) (*n* ≥ 50 for each group). (**F**) Levels of active TGF-β in the cell culture supernatant of dermal fibroblasts incubated with WNT5A with or without ITGAVi (*n* = 6 for each group). (**G**) Representative Western blots of P-SMAD3 in dermal fibroblasts stimulated by WNT5A with and without the ITGAVi (*n* = 3 for each group). The black thin vertical lines were drawn to separate noncontiguous lanes. (**H**) Representative Trichrome stainings and (**I**) Quantification of the dermal thickness, the hydroxyproline content, myofibroblast counts, and active TGF-β in the skin tissue of WNT5A-induced skin fibrosis mice treated with or without ITGAVi (*n* = 5 for each group). Results are shown as median ± IQR. The statistical significance was determined by 1-way ANOVA with Tukey’s multiple comparison test. Adv, Adenovirus.

The term “integrin mediated signaling pathway” was amongst the GO term upregulated by WNT5A (Figure 2C). IPA analysis also predicted a role of integrin signaling. Multiple lines of evidence demonstrate that integrin α_V_ (ITGAV) can activate latent TGF-β (47–51). This requires the assembly of ITGAV clusters in focal adhesions and the subsequent assembly of large integrin-adhesion complexes (52, 53). Accumulation of phosphorylated PAXILLIN and TALIN (P-PAXILLIN and P-TALIN, respectively) serves as a marker for the maturation of focal adhesions and the formation of integrin-adhesion complexes. Confocal microscopy demonstrated that WNT5A induced the formation of large ITGAV clusters in fibroblasts (Figure 5A and Supplemental Figure 11, A and B). In contrast, WNT5A did not promote clustering of ITGA5 (Supplemental Figure 11C). Increased levels of phosphorylated PAXILLIN and TALIN in WNT5A-stimulated fibroblasts provided further evidence for WNT5A-induced formation of integrin-adhesion complexes (Figure 5B). Confocal imaging with z-stacks showed that WNT5A induced colocalization of the LAP of the TGF-β propeptide with ITGAV and P-PAXILLIN (Figure 5C), demonstrating that WNT5A promotes the interaction of LAP with ITGAV at mature focal adhesions. Consistently, magnetic tweezer experiments showed that stimulation with WNT5A increased the rupture force of LAP-TGF-β1 peptide-coated magnetic beads from fibroblasts (Figure 5, D and E). Incubation with the ITGAV inhibitor CWHM12 (ITGAVi) prevented the WNT5A-induced activation of latent TGF-β (Figure 5F) and the accumulation of the downstream mediator P-SMAD3 (Figure 5G). Inactivation of ITGAV also blocked the upregulation of fibrosis-relevant genes by WNT5A (Supplemental Figure 12, A–D) and inhibited WNT5A-induced fibroblast-to-myofibroblast transition in vitro (Supplemental Figure 12, E–G). Moreover, treatment of Wnt5a-overexpressing mice with ITGAVi abrogated Wnt5a-induced dermal fibrosis and normalized the levels of active TGF-β in fibrotic skin (Figure 5, H and I).

### The profibrotic effects of WNT5A depend on rapid coordinated cytoskeletal rearrangement.

**Figure 6:**
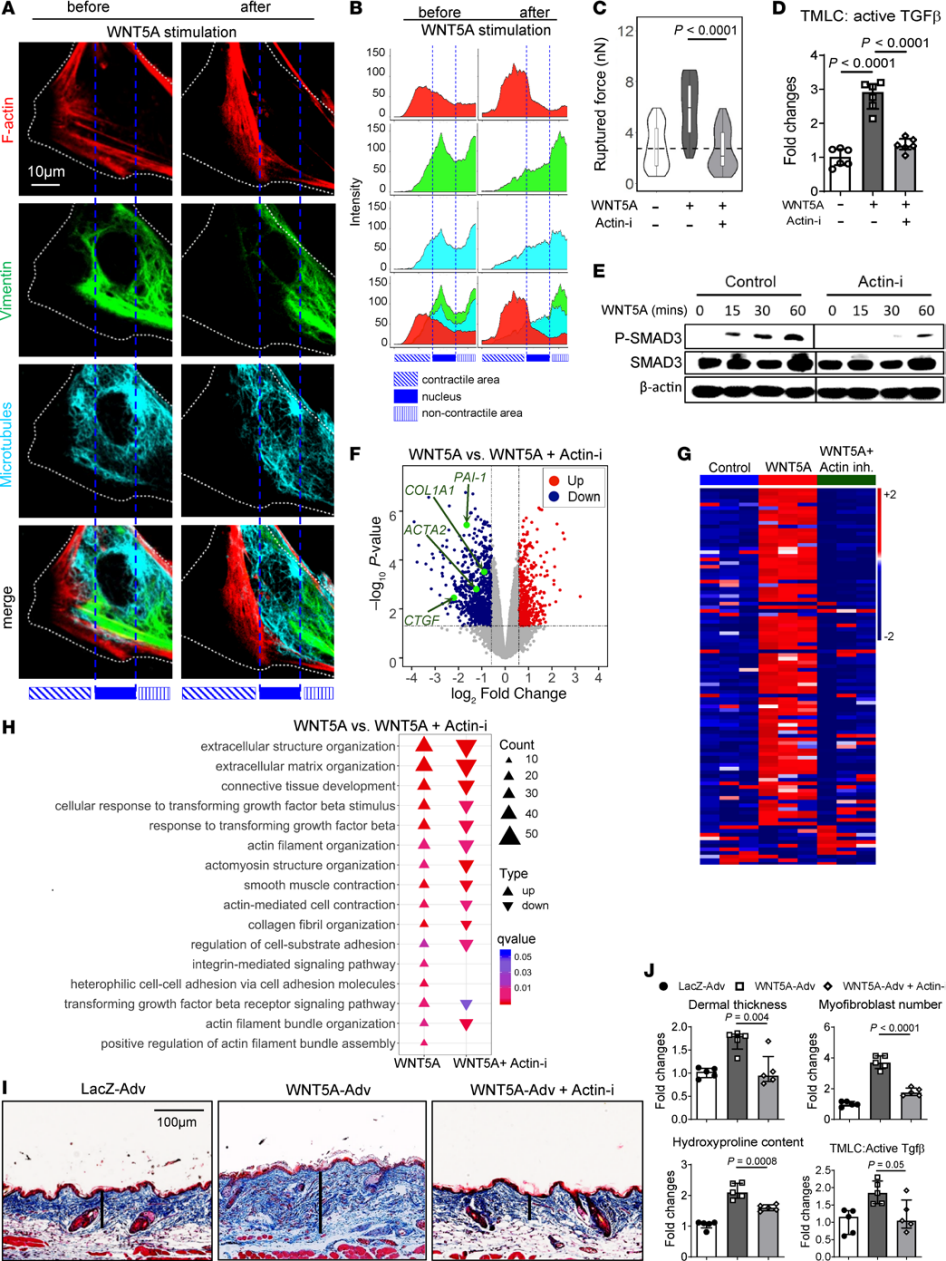
WNT5A induces coordinated cytoskeletal changes to promote activation of latent TGF-β. (**A**) Representative images showing WNT5A-induced changes in F-actin, Vimentin filaments, and microtubules with (**B**) quantification of each cytoskeletal component in relation to its subcellular localization. (**C**) Violin plots showing the force required to rupture magnetic beads coupled with LAP-TGF-β1 peptides from dermal fibroblasts incubated with WNT5A with or without the actin inhibitor cytochalasin D (*n* ≥ 50 for each group). (**D**) Levels of active TGF-β in the supernatant of dermal fibroblast incubated with WNT5A in the presence or absence of cytochalasin D measured by TMLC assays (*n* = 6 for each group). (**E**) Representative Western blots showing the levels of P-SMAD3 in dermal fibroblasts stimulated with WNT5A and cytochalasin D (*n* = 3 for each group). The black thin vertical lines were drawn to separate noncontiguous lanes. (**F**) Volcano plot and (**G**) heatmap illustration of DEGs from RNA-Seq of WNT5A-stimulated human dermal fibroblasts treated with Cytochalasin D or with vehicle. (**H**) Deenrichment of GO biological processes related to fibroblast-to-myofibroblast transition and fibrosis (*n* = 3 for each group). (**I**) Representative Trichrome stainings, and (**J**) quantification of the dermal thickness, hydroxyproline content, myofibroblast counts, and active TGF-β in skin lysates from mice with WNT5A-induced skin fibrosis with or without ITGAV inhibitor (*n* = 5 for each group). Results are shown as median ± IQR with data representing individual data points. The statistical significance was determined by 1-way ANOVA with Tukey’s multiple comparison test. Adv, Adenovirus; Actin-i, Actin inhibitor.

Complex formation between ITGAV-containing integrin heterodimers and the TGF-β propeptide alone is not sufficient for latent TGF-β activation, but requires also tensile force to unfasten the straitjacket elements that surround TGF-β (54, 55). We hypothesized that WNT5A may also induce cytoskeletal reorganization to promote tensile force for latent TGF-β activation. Indeed, RNA-Seq demonstrated that multiple GO- and GSEA-gene sets related to cytoskeletal organization were regulated by WNT5A in dermal fibroblasts (Figure 2C and Supplemental Figure 2E). Moreover, IPA analysis suggested a WNT5A-dependent regulation of the actin cytoskeleton. Given the rapid increases in active TGF-β after WNT5A stimulation, the activation of TGF-β by WNT5A cannot be explained by enhanced contraction due to neoexpression of contractile proteins such as α-SMA during fibroblast-to-myofibroblast differentiation. To study early cytoskeletal changes, we performed live cell imaging in human dermal fibroblasts before and up to one hour after WNT5A stimulation. WNT5A promoted the accumulation of filamentous actin (F-actin) with simultaneous declines in Vimentin-filaments and microtubules at the contracting front of fibroblasts (Figure 6, A and B, Supplemental Figure 13, A and B, and Supplemental Video 1). Consistently with F-actin formation and degradation of Vimentin-filaments (Vimentin) and microtubules (Tubulin), WNT5A increased the ratios of F-actin to globular actin (G-actin), of soluble to insoluble Vimentin, and of short to long Tubulin (Supplemental Figure 14, A–F). WNT5A also induced colocalization of F-actin with ITGAV and P-PAXILLIN in human dermal fibroblasts, consistent with a WNT5A-induced, F-actin–mediated maturation of focal adhesions (Supplemental Figure 13C).

We next evaluated the roles of JNK and ROCK in those WNT5A-induced cytoskeletal rearrangements. Inhibition of JNK or ROCK blocked the WNT5A-induced cytoskeletal changes (Supplemental Videos 2 and 3, and Supplemental Figure 14, A–F) and prevented the accumulation of P-PAXILLIN and of P-TALIN (Supplemental Figure 15, A and B) as well as integrin clustering (Supplemental Figure 15, C and D). Consistently, inhibition of JNK or ROCK abrogated the WNT5A-induced increases in the rupture force of magnetic beads coated with LAP-TGF-β1 peptides from the cell membrane of fibroblasts (Supplemental Figure 15E).

Inhibition of actin polymerization by Cytochalasin D (Actin-i) prevented WNT5A-induced integrin clustering (Supplemental Figure 16A), strengthening of LAP-TGF-β peptides binding to integrins (Figure 6C), TGF-β activation (Figure 6D), P-SMAD3 upregulation (Figure 6E), and mRNA upregulation of the TGF-β target genes *PAI-1* and *CTGF* (Supplemental Figure 16C). Actin inhibition also abrogated the induction of profibrotic genes, GO- and GSEA gene sets in human dermal fibroblasts (Figure 6, F–H and Supplemental Figure 16B), fibroblast-to-myofibroblast transition (Supplemental Figure 16, D and E), and Wnt5a-induced tissue fibrosis (Figure 6, I and J).

Together, these data suggest that WNT5A activated JNK and ROCK to induce coordinated rearrangement of actin fibers, Vimentin-intermediate filaments, and microtubules to promote ITGAV clustering in mature focal adhesions to activate latent TGF-β (Supplemental Figure 17).

### Knockout of Wnt5a prevents aberrant activation of latent TGF-β signaling and ameliorates experimental fibrosis.

**Figure 7:**
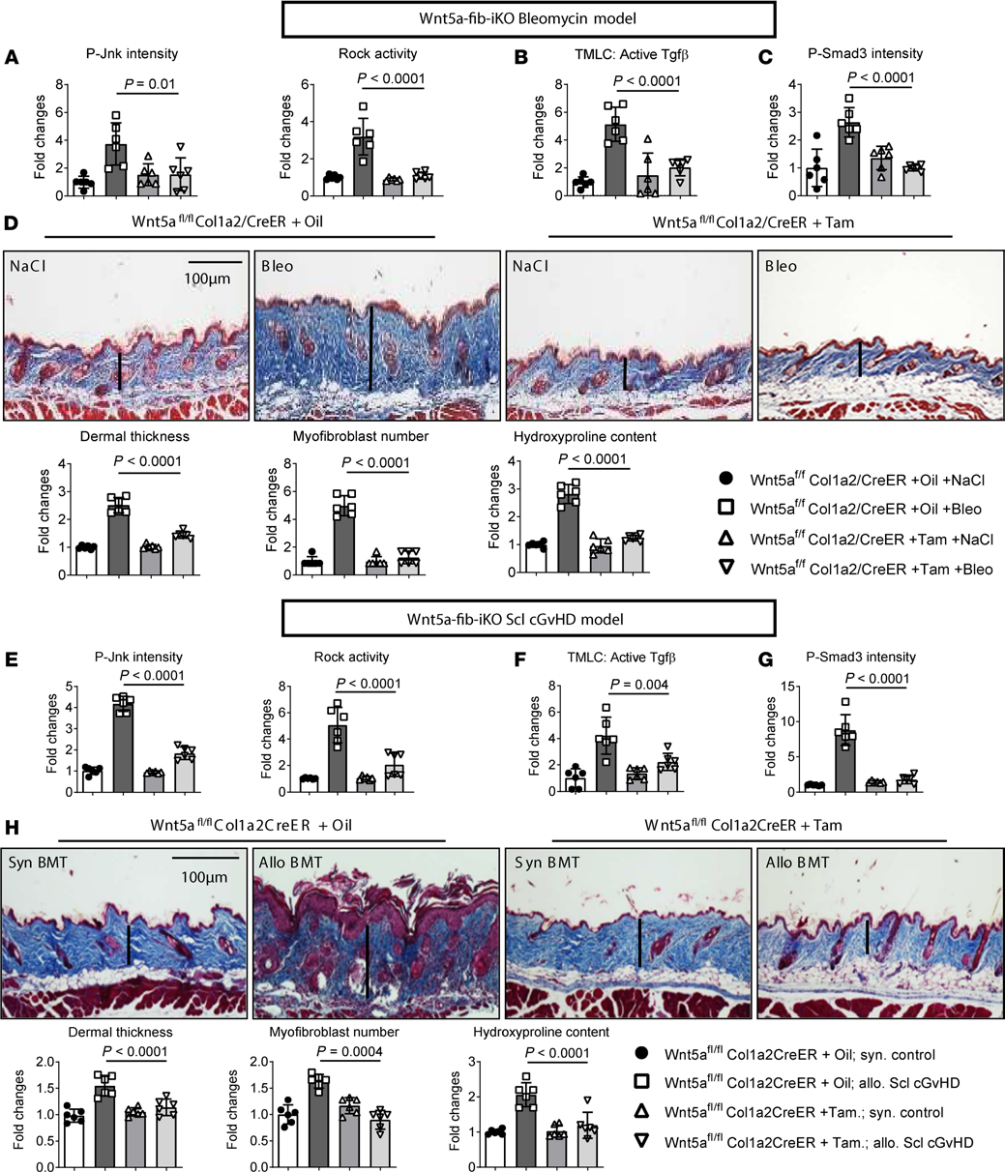
Inactivation of Wnt5a signaling ameliorates experimental skin fibrosis. (**A**–**D**) Fibroblast-specific knockout of Wnt5a in bleomycin-induced skin fibrosis. (**A**) Quantification of the IF staining of P-Jnk and of the Rock activity. (**B**) Levels of active TGF-β measured by TMLC. (**C**) Quantification of the IF staining for P-Smad3. (**D**) Representative Trichrome stainings and quantification of dermal thickness, myofibroblast counts, and hydroxyproline content. (**E**–**H**) Fibroblast-specific knockout of Wnt5a in murine scl cGvHD. (**E**) quantification of the IF staining for P-Jnk and of Rock activity. (**F**) Level of active TGF-β measured by TMLC. (**G**) Quantification of the IF staining for P-Smad3. (**H**) Representative trichrome stainings and quantification of the dermal thickness, myofibroblast counts, and hydroxyproline content (*n* = 6 for each group). Results are shown as median ± IQR with data representing individual data points. The statistical significance was determined by 1-way ANOVA with Tukey’s multiple comparison test. Bleo, Bleomycin; Tam, Tamoxifen; Syn, Syngenic; Allo, Allogenic; BMT, Bone marrow transplantation.

To investigate the therapeutic potential of targeting WNT5A, we generated 2 mouse models with inducible deletion of Wnt5a: Wnt5a^fl/fl^ × Col1a2/CreER mice (56) with fibroblast-specific deletion of Wnt5a upon challenge with tamoxifen (Wnt5a-fib-iKO), and Wnt5a^fl/fl^ × ubc/CreER mice with tamoxifen-inducible ubiquitous deletion of Wnt5a (Wnt5a-ubc-iKO). Knockout of Wnt5a did not induce compensatory changes in other noncanonical WNT ligands. Bleomycin-induced skin fibrosis was ameliorated in Wnt5a-fib-iKO mice. Knockout of Wnt5a prevented the activation of JNK- and ROCK-signaling to levels comparable to that of control littermates (Wnt5a^fl/fl^ × Col1a2/CreER mice injected with corn oil) (Figure 7A). Of note, knockout of Wnt5a completely abrogated the bleomycin-induced activation of latent TGF-β with levels of active TGF-β in Wnt5a-fib-iKO comparable to that of nonfibrotic mice (Figure 7B). Consistently, knockout of Wnt5a prevented the bleomycin-induced accumulation of P-Smad3 (Figure 7C). Inhibition of TGF-β activation translated into protection from experimental fibrosis. Hence, Wnt5a-fib-iKO challenged with bleomycin demonstrated reduced dermal thickening, lower myofibroblast numbers, and decreased hydroxyproline content as compared with controls (Figure 7D). Wnt5a-ubc-iKO mice were protected from bleomycin-induced skin fibrosis to an extent comparable with Wnt5a-fib-iKO mice, demonstrating that fibroblasts are a major source of Wnt5a in fibrotic diseases (Supplemental Figure 18, A–D). We noted that the KO efficiency in mice with fibroblast-specific knockout of Wnt5a (Wnt5a^fl/fl^ × Col1a2/CreER) was around 80% in fibroblasts (defined as Vimentin^+^ cells), with no obvious change of expression in other cells. In mice with ubiquitous knockout of WNT5A (Wnt5a^fl/fl^ × ubc/CreER), the KO efficiency is approximately 85%, both in Vimentin^+^ and Vimentin^–^ cells (Supplemental Figure 19, A and B). To further confirm that Wnt5a expression by fibroblasts was required for experimental fibrosis, we evaluated the outcome of Wnt5a-fib-iKO mice in the LP/J (H-2^b^) → C57/Bl6 (H-2^b^) model of scl cGvHD (57, 58). Wnt5a-fib-iKO showed reduced levels of P-Jnk and impaired Rock activity (Figure 7E). As in bleomycin-induced skin fibrosis, knockout of Wnt5a reduced the increase in the levels of active TGF-β in allogeneically transplanted mice (Figure 7F) and prevented the upregulation of P-Smad3 (Figure 7G). Wnt5a-fib-iKO mice also demonstrated less dermal thickening, reduced myofibroblast counts, and decreased hydroxyproline content (Figure 7H).

## Discussion

We demonstrate that β-catenin–independent, noncanonical WNT5A signaling is active in human fibrotic diseases such as SSc, cGvHD, and IPF. WNT5A did not only show the highest expression levels of all noncanonical WNT ligands, but also the most pronounced differences in expression levels compared with matched nonfibrotic tissues. Expression analyses together with functional data from fibroblast-specific and ubiquitous depletion of Wnt5a characterized fibroblasts as a major source of WNT5A in the skin, suggesting an auto- or paracrine mode of action of WNT5A. Although WNT5A is the dominant noncanonical WNT ligand in SSc, differences in the expression levels of other ligands may further enhance the activation of WNT/β-catenin–independent signaling, and other noncanonical WNT ligands may be dominant in other fibrotic diseases (59–61). So far, only WNT/β-catenin signaling was considered as a core pathway in the pathogenesis of fibrotic diseases (26, 62–64). Evidence for a role of WNT5A was scarce; Li et al. demonstrated that nonconditional inactivation of Wnt5a interrupted alveologenesis by reducing fibroblast-to-myofibroblast transition (65). Our data now highlight that noncanonical WNT signaling is sufficient and required for fibroblast-to-myofibroblast transition and the development of fibrosis.

The profibrotic effects of WNT5A were mediated by activation of JNK and ROCK. Consistent with a central role of JNK and ROCK signaling in the pathogenesis of fibrosis, increased activation of JNK and ROCK signaling have been reported in human fibrotic diseases such as SSc (34, 66–68), but this activation has not been associated with noncanonical WNT signaling before.

Depending on the cellular context and the target tissue, noncanonical WNT signaling cascades may affect WNT/β-catenin signaling (38, 39, 61, 69, 70). However, our study provides strong evidence that the profibrotic effect of WNT5A signaling does not require WNT/β-catenin signaling. WNT5A does not induce nuclear accumulation of β-catenin or promote β-catenin/TCF-dependent transcription in cultured fibroblasts or in fibrotic skin. In contrast, targeted inhibition of JNK and ROCK downstream of WNT5A is sufficient to abrogate the profibrotic effects of WNT5A. WNT/β-catenin- and WNT5A/JNK/ROCK signaling may thus represent independent targets for antifibrotic therapies, and combined inhibition of canonical as well as noncanonical WNT signaling may exert additive effects. Combined inhibition of both signaling cascades could be achieved by porcupine inhibitors (64, 71), which are currently tested in clinical trials for the treatment of various cancers (72).

A central finding of our study is that WNT5A is a critical molecular upstream regulator of the aberrant activation of latent TGF-β in fibrotic diseases. WNT5A activates JNK and ROCK to induce coordinated rearrangement of all 3 main components of the cytoskeleton, microtubules, vimentin-containing intermediate filaments, and actin filaments, to promote contraction-induced tensile forces. These forces are transmitted via focal adhesions and ITGAV to the propeptide of TGF-β to promote its release as an active growth factor (14, 73). Interferences at any stage of this process, either by knockout of WNT5A or inhibition of JNK, ROCK, ITGAV, or actin-polymerization, abrogates the increase of active TGF-β in cultured fibroblasts and in murine models of skin fibrosis, highlighting that this pathway is essential for the increased activation of latent TGF-β. We provide several lines of evidence that WNT5A promotes TGF-β signaling at the level of activation of latent TGF-β. Neutralizing antibodies against TGF-β are equally effective as inhibition of TGF-βRI kinase activity. These findings argue against major effects of other potential signal transduction pathways such as transphosphorylation of TGF-βRI or TGF-βRII by active WNT receptors or of direct phosphorylation of SMAD by JNK in fibrosis (41, 42). In addition, the upregulation of active TGF-β and activation of intracellular targets such as SMAD3 occurs rapidly within a few minutes after WNT5A stimulation, and mRNA and total protein levels of TGF-β1 did not change. Further, inhibition of ITGAV abrogated the stimulatory effects of WNT5A on TGF-β signaling. Impaired migration and proliferation in response to WNT5A have been described upon inhibition of ITGAV (74). Finally, the release of FLAG-tagged TGF-β1 from fibroblasts transfected with LAP/FLAG-TGF-β1 upon WNT5A stimulation further supports this mode of action.

The stimulatory effects of WNT5A on TGF-β activation indicate that WNT5A may account for the persistent activation of TGF-β signaling in fibrotic diseases. Indeed, stimulation with WNT5A induces a typical TGF-β biased gene signature in resting fibroblasts that is characteristic for fibroblasts isolated from fibrotic tissues (75). Overexpression of WNT5A in skin or lung is sufficient to activate TGF-β signaling as in fibrotic tissues. Selective knockout of WNT5A or inhibition of its downstream mediators JNK, ROCK, or ITGAV, or of actin-polymerization prevents the increase in active TGF-β and the aberrant transcription of TGF-β target genes in experimental fibrosis. These findings provide evidence that WNT5A is a central upstream regulator of TGF-β activity in fibrotic disease.

Our study focuses specifically on the role of WNT5A in fibroblasts and does not analyze its role in other cells relevant for fibrotic tissue remodeling. However, for a potential therapeutic approach with systemic inhibition of WNT5A signaling in fibrotic diseases, one needs to consider other relevant cell types in addition to fibroblasts. Indeed, WNT5A has recently been shown to induce alternative activation of macrophages, which is thought to be a central pathological mechanism in fibrotic diseases (76). This highlights that potential therapeutic inhibition of WNT5A may target different cellular players and different pathological mechanism of fibrosis. However, further studies are required to explore the therapeutic potential of targeting WNT5A in SSc and other fibrotic diseases. Further studies are also required to determine how stable WNT5A-induced fibrosis is and whether it might be regressive in the absence of hyperactive WNT-signaling. If so, a short but intense inhibition of WNT5A signaling might be sufficient to break the vicious cycle of WNT5A overexpression, activation of latent TGF-β and fibrosis.

In summary, we demonstrate that WNT5A activates JNK- and ROCK-signaling to trigger coordinated cytoskeletal reorganization, which generates F-actin-mediated tensile forces that induce ITGAV-dependent activation of latent TGF-β in fibrotic diseases (Supplemental Figure 17). Overexpression of WNT5A is sufficient and required to induce aberrant activation of latent TGF-β and to promote tissue fibrosis. Targeted inhibition of this pathway prevents aberrant TGF-β signaling in fibrotic conditions and exerts potent antifibrotic effects. These findings may have translational implications as pharmacologic approaches to interfere with this pathway at different levels are available for clinical programs.

## Methods

### Sex as a biological variant.

In this study, sex was not considered as a biological variable.

### Statistics.

All in vitro and in vivo data are presented as median with interquartile range (IQR) with data representing individual data points. The statistical significance was determined by 2-tailed Mann-Whitney *U* test if two groups were compared, a 1-way ANOVA with Tukey’s multiple comparison test in case of more than 2 comparisons, or 2-way ANOVA with Bonferroni’s multiple comparison test in case of multiple groups comparisons. In a subset of experiments, the mean values of the control groups were set to 1. All other values were expressed as fold changes compared with the respective controls used as ‘comparison mean values’. *P* values less than 0.05 were considered significant.

### Study approval.

The study was approved by the ethical committee of the University Hospital Erlangen. All participants gave their written informed consent. Mouse studies were approved by the government of lower, middle, and upper Franconia according to the EU animal welfare standard.

### Data availability.

All data generated during this study are included in this published article (and its supplementary information files). All the RNA-Seq data were deposited in NCBI/GEO with the study number GSE222916. Additional detailed information is available from the corresponding author on request.

Detailed information on material and methods is provided as supplementary information due to restrictions in word count.

## Author contributions

TTM, CWC and JHWD designed the research. TTM, CWC, CTM, YNL, HZ, XZ, YZ, DC, SR, CD, NYL, DK, RG, CB, CR, FGB, BF, AR, and OD performed, analyzed and interpreted the research. DK, RG, and BF designed experiments involving magnetic tweezers and microtissues. AK and BE provided essential material. TTM, CTM, and JHWD designed, performed, and analyzed the experiments for revision. TTM, CWC, AS, GS and JHWD wrote the manuscript. TTM and CWC contributed equally to this work.

## Supplementary Material

Supplemental data

Unedited blot and gel images

Supplemental video 1

Supplemental video 2

Supplemental video 3

Supporting data values

## Version 1 05/15/2024

Electronic publication
