# Supplementary material for: Noncanonical WNT5A controls the activation of latent TGF-β to drive fibroblast activation and tissue fibrosis
Source: J Clin Invest. 2024 Mar 26;134(10):e159884. doi: 10.1172/JCI159884 (PMC11093613; doi:10.1172/JCI159884)
Supplement: Supplemental data [file jci-134-159884-s036.pdf]

Supplementary Material

Non-canonical WNT5A controls the activation of latent TGFβ to drive fibroblast activation and tissue fibrosis

Thuong Trinh-Minh[1,2†], Chih-Wei Chen[3,4†], Cuong Tran Manh[1,2],Yi-Nan Li[1,2], Honglin Zhu[3,4,5], Xiang Zhou[1,2], Debomita Chakraborty[3,4], Yun Zhang[1,2], Simon Rauber[3,4], Clara Dees[3,4], Neng-Yu Lin[3,4,6], Delf Kah[7], Richard Gerum[7], Christina Bergmann[3,4], Alexander Kreuter[8], Christiane Reuter[9], Florian Groeber-Becker[9], Beate Eckes[10,11], Oliver Distler[12], Ben Fabry[7], Andreas Ramming[3,4], Alexandra Schambony[13], Georg Schett[3,4], Jörg H.W. Distler[1,2*]

MATERIAL AND METHODS

Human skin biopsies

Skin biopsies were obtained from a total of 23 patients with SSc (sixteen females and seven males; median age: 44 years (range: 19 - 61 years), median disease duration: 4 years (range: 0.5 - 8 years)), 7 patients with scl cGvHD (four females and three males; median age: 49 years (range: 26 - 71 years), median disease duration: ten months (range: 0.4 - 3 years)) and 21 healthy volunteers matched for sex and age. Lung tissues were obtained from a total of seven patients with IPF (five males and two females; median age 57 years (range 49 - 78 years), median disease duration: 2 months (range: 1 - 4 years)) and seven matched non-fibrotic controls. All patients and control individuals gave informed consent as approved by the local institutional review board.

Cell culture

Human dermal fibroblasts were isolated from a total of 12 SSc patients (eight females and four males; median age: 43 years (range: 19 - 61 years), median disease duration: four years (range: 0.5 - 8 years)), and 28 age- and sex-matched healthy volunteers. All patients fulfilled the ACR/EULAR criteria 2013 for SSc (1). Healthy human epidermal keratinocytes were isolated from foreskin tissues which were obtained from medical circumcisions. Fibroblasts and keratinocytes were isolated and cultured according to standard protocols (2, 3). Fibroblasts from passages 4-8 and keratinocytes from passages 2-3 were used for the experiments. Individual sample numbers of each experiment are provided in the respective figure legends.

In selected experiments, cells were incubated with recombinant WNT5A protein (100 ng/ml) (R&D Systems, Wiesbaden-Nordenstadt, Germany), recombinant WNT3A protein (100 ng/ml) (R&D Systems, Wiesbaden-Nordenstadt, Germany), recombinant TGFβ (10 ng/ml) (PeproTech, Hamburg, Germany), the JNK inhibitor SP600125 (125 mM, pre-treatment time is 4 hours) (Sigma-Aldrich, Steinheim, Germany), the ROCK inhibitor Y27632 (1 µM, pre-

treatment time is 1 hour) (Sigma-Aldrich), anti-TGFβ neutralizing antibodies 1D11 (100 ng/ml, pre-treatment time is 4 hours) (R&D Systems), the TGFβRI kinase inhibitor SD-208 (3 µM, pre-treatment time is 4 hours) (Tocris, Nordenstadt, Germany), alpha V-containing integrins inhibitor CWHM12 (1 µM, pre-treatment time is 1 hour) (BOC Sciences, Shirley, NY, USA), or actin polymerization inhibitor Cytochalasin D (10 µM, pre-treatment time is 1 hour) (Abcr, Karlsruhe, Germany).

Full-thickness skin grafts were generated according to established protocols (4). Briefly, a dermal skin layer composing of 1 x 10^5 fibroblasts embedded in a 3D matrix of rat type I collagen was overlayed by 5 x 10^5 keratinocytes on the apical surface. The grafts were cultured in transwell assays. Polarization and differentiation of keratinocytes with formation of a functional basal membrane was initiated by airlift and addition of 1.44 mM $CaCl_2$, 0.125 mM L-ascorbic acid 2-phosphate and 10 ng/mL keratinocytes growth factor to the Epilife medium as maintenance medium. The dermal compartment was maintained in the same medium. The outcome was evaluated after 13 days.

Quantification of cellular contractile forces

Three dimensional contractile forces were quantified in micro-tissue assays (5, 6). Arrays of 18 tissue chambers, each containing a pair of cylindrical pillars with a diameter of 500 µm, were cast using polydimethylsiloxane (PDMS) (Dow Chemical, Michigan, USA) at a ratio of 1:22 (w/w; curing agent to base). For the generation of micro-tissues, 6 µl of a Collagen R and Collagen G mixture (0.6 mg/ml, Matrix BioScience, Mörlenbach, Germany) were added into the chambers. After polymerization, 10,000 human dermal fibroblasts were resuspended in another 6 µl of Collagen R / Collagen G solution and added on the top of the first collagen layer. To determine changes of the deflection and the height of the micro-tissues upon addition of WNT5A, bright field images were taken before and after lysis by 0.1% Triton X-100. The contractile force was calculated by Hooke's law $F = k \times \delta_{tissue}$, where k is the pillars' spring

constant at the height of the tissue, and δ_{tissue} is the deflection of the pillars caused by tissue contraction. All images were captured on a Leica DMI 6000 CS microscope (Leica, Wetzlar, Germany), and image analysis was performed using ClickPoints, a software framework for image based tracking (7).

Transfection

Primary human fibroblasts were transfected using the 4D-Nucleofector (Lonza, Cologne, Germany). Plasmids and prevalidated siRNAs used in the study are listed (Supplementary table 1). For plasmids, 3 μg were used and the transfection efficiency was determined by co-transfection with pSv-β-galactosidase vectors (Promega, Mannheim, Germany). For siRNA mediated knockdown of target genes, fibroblasts were transfected with 3 μg of prevalidated siRNAs. Non-targeting siRNAs served as controls.

Quantitative real time-PCR

Gene expression was quantified by SYBR Green real-time PCR using the StepOne Plus System (Thermo Fisher Scientific, Waltham, MA, USA). Primers are listed in Supplementary table 2. Samples without enzyme in the reverse transcription reaction (Non-RT controls) were used as negative controls. Unspecific signals caused by primer dimers were excluded by non-template controls and by dissociation curve analysis. The gene expression level was calculated with the threshold cycle (Ct) and the comparative Ct method (referred to as the $2^{-\Delta\Delta CT}$ method) for relative quantification. Beta-actin was used to normalize the amounts of cDNA within each sample.

Western blot analysis

Proteins were separated by SDS-PAGE and transferred to a polyvinylidene difluoride (PVDF) membrane. The membrane was incubated with the appropriate primary antibodies (listed in

Supplementary table 3) and HRP-conjugated secondary antibodies (Dako Cytomation, now Agilent Technologies, Glostrup, Denmark). Signals were visualized by ECL. Beta-actin and Lamin A / C were used as loading controls. Western Blots were quantified using the ImageJ Software (version 1.41).

Quantification of collagen protein

The amount of soluble collagen in cell culture supernatants was quantified using the SirCol collagen assay (Biocolor, Belfast, Northern Ireland) (8). The total collagen content of tissue samples was determined by hydroxyproline assays (9).

Immunohistochemistry, immunofluorescence and immunocytochemistry

Formalin-fixed, paraffin-embedded skin sections or 4 % PFA-fixed, 0.25 % Triton X100-permeabilized cells were stained with appropriate primary antibodies (Supplementary table 3). HRP-conjugated or Alexa Fluor antibodies (Thermo Fisher Scientific) were used as secondary antibodies. Isotype control antibodies served as controls. Stress fibers were visualized with rhodamine-conjugated phalloidin (Sigma-Aldrich). Nuclei were stained using DAPI (Santa Cruz Biotechnology, Heidelberg, Germany). Myofibroblasts were identified as single cells double-positive for αSMA along with either vimentin (mice) or P4H. Stainings were visualized using a Nikon Eclipse 80i microscope (Nikon, Badhoevedorp, Netherlands), a Zeiss LSM700 confocal microscope (Carl Zeiss, Jena, Germany) or Leica SP5 II Fast Resonant Scanner (Leica).

Quantification of myofibroblast markers

Human dermal fibroblasts were fixed with 4% paraformaldehyde and the cell membranes were labeled with Alexa Fluor 647-conjugated wheat germ agglutinin (WGA) (Thermo Fisher Scientific). Fibroblasts were further permeabilized with 0.2% Triton X-100 and stained with

antibodies against α-SMA (Sigma-Aldrich) or with rhodamine phalloidin (Thermo Fisher Scientific). Imaging and analysis were performed by CellInsight CX5 High Content Screening Platform (Thermo Fisher Scientific) with Cellomics® Compartmental Analysis V4 BioApplication to quantify the cellular fluorescence intensity.

RNA sequencing library construction and bioinformatic analysis

Total RNA from healthy dermal fibroblasts stimulated with or without recombinant WNT5A protein or recombinant TGFβ and treated with or without the JNK inhibitor SP600125, the ROCK inhibitor Y27632, the TGFβRI kinase inhibitor SD-208, ITGAV inhibitor CWHM12, or Cytochalasin D was extracted as described previously (10). RNAseq was performed by Novogene Co., Ltd. (Cambridge, United Kingdom) on an Illumina NovaSeq platform using a paired-end 150 bp sequencing strategy. The mRNA was reverse transcribed into cDNA using random hexamers. The bioconductor package edgeR was used for differential expression analyses of the RNAseq read counts. The TMM method was used for normalization. Quasi-likelihood F-tests were used for calculating the statistically significant differentially expressed genes (DEGs) by an adjusted p-value threshold of < 0.05 and fold change > 1.5. The results are presented as Volcano plots by using the R package EnhancedVolcano (Github) according to the Log_2FC and $–Log_{10}p$-value of DEGs in different groups. Hierarchical cluster analyses were performed using the R package pheatmap. GO pathway enrichment analyses were performed using the R package clusterProfiler (11) and a q-value < 0.05. Ridgeline plots were generated by the R package ggplot2 and ggridges.

ROCK activity assay

The ROCK kinase activity was measured by using a commercially available ROCK activity assay (Millipore Corporation, Billerica, MA, USA). Briefly, lysates of human dermal fibroblasts were incubated in cell culture plates pre-coated with recombinant MYPT1. ROCK

induced phosphorylation of MYPT1 was detected with anti-phospho-MYPT1 (Thr696) antibodies and HRP-conjugated secondary antibodies. The absorbance was measured at 450nm using a GloMax Discover microplate reader (Promega, Walldorf, Germany).

Reporter assays

TOP/FOP reporter assays: Human dermal fibroblasts were transfected with TOP Flash plasmids (wild-type) and FOP Flash plasmids (Millipore Corporation) containing three copies of normal or mutated Tcf consensus binding motifs upstream of a firefly luciferase DNA sequence. A β-galactosidase reporter vector was used as a transfection control (12).

SMAD binding element (SBE) reporter assays: Human dermal fibroblasts were infected with 1,000 IFUs of CAGA-Luc AAV encoding the luciferase enzyme under a CAGA promoter (13) or respective control AAVs.

Luciferase and β-galactosidase activities were determined using a GloMax Discover microplate reader (Promega).

Live-cell imaging for cytoskeleton rearrangement

To monitor the dynamic change of cytoskeleton compartments, we transfected human dermal fibroblasts with pLifeAct_mScarlet_N1 (14) (kindly provided by Philipp Tripal from the Optical Imaging Centre Erlangen) and Vimentin-Chromobody TagGFP plasmids (ChromoTek, Planegg-Martinsried, Germany). Microtubuli were stained with 1 µM SiR-tubulin (Spirochrome, Stein am Rhein, Switzerland) one hour before imaging. Images were captured every 3 mins for a period of 30 mins before and 90 mins after WNT5A stimulation under 37°C and 5% CO_2 by using the Zeiss Spinning Disc Axio Observer Z1 microscope (Carl Zeiss) equipped with Evolve ® 512 EMCCD Camera (Teledyne Photometrics, Tucson, AZ, USA). JNK and ROCK inhibitors were added four hours and one hour before imaging, respectively. Videos were generated using ImageJ.

Quantification of active TGFβ by transformed mink lung cell (TMLC) assay

The level of active TGFβ was evaluated by mink lung cells transfected with a fused sequence of firefly luciferase and PAI-1 promoter (kindly provided by Daniel Rifkin, New York, NY, USA) (15). TMLC assays were performed as previously described (16). In brief, fibroblast-conditioned medium and murine skin lysates were added to the medium of pre-plated TMLC. After 24 hours of incubation, luciferase reporter activity was determined by using a GloMax Discover microplate reader (Promega). The luminescence intensity was converted into concentrations by using a standard curve with a serial dilution of recombinant TGFβ.

LAP-TGFβ fusion constructs

Human dermal fibroblasts were transfected with a FLAG-tagged TGFβ-LAP construct with a cleavage exposed FLAG-tag, placed in between the coding regions of LAP and TGFβ, which remains with the active TGFβ moiety after cleavage (17) (schematic presentation in Figure 4F). After stimulation with recombinant WNT5A, the cell culture supernatants were collected and incubated with A/G agarose beads (Santa Cruz Biotechnology) conjugated with LAP antibodies (R&D Systems) to precipitate and remove any latent TGFβ. After purification, the supernatants were then transferred to ELISA plates coated with anti-FLAG antibodies (Thermo Fisher Scientific) and then incubated with biotinylated anti-TGFβ1 antibodies (Thermo Fisher Scientific). In addition, the specificity of those measurements was further confirmed by quantification of any remaining LAP (including any potential latent TGFβ) using biotinylated anti-LAP antibodies (R&D Systems). Signals were detected using a HRP-Streptavidin (R&D Systems) and TMB ELISA substrate (Biolegend, San Diego, CA, USA) and evaluated at 450 nm and 550 nm using a GloMax Discover microplate reader (Promega).

Quantification of ITGAV clustering

Quantification of integrin clustering was performed as described previously (18). First, acrylamide gels were generated and coated with type I collagen following established protocols (19). The gels had a stiffness of 4 kPa, which is reminiscent of the stiffness of human skin (20). Human dermal fibroblasts were cultured on these gels and stimulated with recombinant WNT5A (100 ng/ml) (R&D Systems). The cells were stained using anti-ITGAV antibodies conjugated with Alexa Fluor 594 (Abcam, Cambridge, UK) and the membrane dye WGA conjugated with Alexa Fluor 647 (Thermo Fisher Scientific). Single-cell images were captured on an inverted confocal microscope Leica SP5 II Fast Resonant Scanner**.** The intensity and the area of the ITGAV staining associated with the cell membrane in mature clusters (defined by a size $\geq 1\mu m^2$ and diameter > 1µm as published previously (21)) were assessed using the Fiji software (22).

Multicolor single-molecule colocalization analysis

Human dermal fibroblasts were fixed with 4% PFA/ 0.1% Triton X-100 and stained for latent TGFβ1 (LAP), ITGAV, phospho-PAXILLIN and F-actin and DAPI as described above. 16-bit single-cell images were captured on an inverted confocal microscope Leica SP5 II Fast Resonant Scanner with z-stack settings of 40 nm per stack. The multichannel images were processed using the Fiji software (22) and analyzed by the Coloc 2 plugin for Fiji to achieve Pearson’s R value, Manders’ values and Coastes p-values. The images were further analyzed using the Colocalization threshold plugin and Comdet plugin (Github, San Francisco, CA, USA) with default setting to obtain scatter plots and to quantify colocalization and intensity across channels. Data from the Comdet analysis were visualized as 3D scatter plots using the R software (version 3.1.2) and the scatterplot3d package (version 0.35).

Fractionation of cytoskeletal components

Fractionation of detergent-soluble and detergent-insoluble vimentin, of globular (G)- and filamentous (F)-actin and of microtubules and soluble tubulin was performed as described (23). For vimentin, human fibroblasts were lysed upon stimulation with WNT5A on ice in low-detergent buffer (containing 10mM phosphate buffer, 50mM 3-(N-morpholino) propanesulfonic acid (MOPS), 10mM $MgCl_2$, 1mM ethylene glycol tetra-acetic acid (EGTA), 1mM phenylmethanesulfonylfluoride (PMSF), 50mM NaF, 1mM Na_3VO_4, and 0.2% Triton-X100) for 5 mins. The detergent-soluble fraction was collected by centrifugation at 3000 xg for 5 mins. The pellet containing the detergent-insoluble fraction was resuspended in the equal volume of 1x boiling Laemmli buffer.

For the assessment of G- and F-actin fractions, cells were lysed in an actin-filament stabilization buffer (Cytoskeleton Inc., Denver, CO, USA). G- and F-actin fractions were separated by ultracentrifugation at 150,000 xg for 1.5 hours.

For quantification of soluble and insoluble tubulin fractions, fibroblasts were incubated with lysis buffer and microtubule stabilization buffer (Cytoskeleton Inc). The lysate was centrifuged at 1,000 xg for 5 mins to separate soluble tubulin in the supernatant and microtubules associated with the nuclei or the Golgi apparatus in the pellet. The supernatant was further separated by ultracentrifugation at 200,000 xg for 1 hour into the soluble tubulin fraction and short microtubules.

For all fractionation experiments, the different fractions were adjusted to equal volumes and the amount of the respective cytoskeletal proteins in the different fractions was then quantified by Western blot.

Measurement of receptor-ligand binding strength with magnetic tweezers

To monitor the binding strength between LAP of the latent TGFβ complex and ITGAV, magnetic tweezers experiments were performed using established protocols with minor modifications (24). Briefly, 5×10^7 epoxylated beads with a diameter of 4.5 µm (Dynabeads

M-450, Thermo Fisher Scientific) were coupled with 10 µg of a synthetic LAP peptide containing its RGD site and neighboring sequences (Ac-R**RGD**LATISPASSKGGGGSRLLLLLLR-NH_2) in 1 ml of sterile carbonate buffer pH 9.5 (Biolegend) for 16 hours at 4 ^oC. Following 24 hours of serum starvation, 3 x 10^4 primary human fibroblasts were incubated with 3 x 10^5 RGD-coated beads for 20 mins and then washed extensively with FBS-free DMEM/F-12 medium to remove unbound beads. The force required to detach a bead from the cell surface (rupture force) was determined by linear increase of the magnetic force on the individual beads from 0 to 10 nN within 3 seconds.

Overexpression of WNT5A in human dermal fibroblasts

To confirm the effects of recombinant WNT5A stimulation on fibroblasts, various multiplicity of infections (MOI) of adenovirus overexpressing WNT5A (AdWNT5A, developed in-house) were used to introduce fibroblasts at 40, 80, and 120. Adenoviruses encoding for LacZ (AdLacZ) served as a controls. To quantify collagen production, αSMA expression, pSMAD3, and pJNK activation via Western blot, the fibroblasts infected with adenovirus were incubated at 37°C and 5% CO_2 for 48 hours. Integrin alpha V clustering or cytoskeleton rearrangement were assessed after 6-12h using confocal microscopy. The results obtained with in-house developed AdWNT5A virus was validated using an additional adenovirus (Vector Biosystems, ADV-227832) that expressed WNT5A together with mCherry as a reporter. Infection with the empty viral vector at the same MOIs served as control.

Overexpression of Wnt5a in the skin and lungs of mice

Wnt5a was overexpressed locally in the skin or lungs of mice using adenovirus (Adv) encoding for Wnt5a (in-house). Adv encoding for LacZ served as controls. Sex was not considered as a biological variable.

Overexpression in the skin was obtained by intracutaneous injections of 1 x 10^8 ifus once per month into male DBA/2 mice (Janvier, Le Genest-Saint-Isle, France) starting at the age of four weeks. In a subset of experiments, mice were treated with CC930 (25) (Celgene, Summit, NJ, USA) (150µg/kg by oral gavage bid) or with Y27632 (26) (100 µg/kg by intraperitoneal injection), with the neutralizing anti-TGFβ antibody 1D11 (1.5 mg/kg by intraperitoneal injection) (27), with Cytochalasin D (1 mg/kg/day by continuous infusion) (28) or with CWHM12 (100 mg/kg/day continuous infusion) (29). Mice receiving the respective solvents of the inhibitors or antibodies served as controls. The outcomes were analyzed after three months. Overexpression of Wnt5a in the lungs was induced by single intratracheal instillation of Advs (1 x 10^8 ifus) in six week old C57BL/6 mice (Janvier) using a high-pressure syringe with a nebulizer (Penn-Century, Wyndmoor, PA, USA). The mice were sacrificed for further analysis after four weeks.

Inducible fibroblast-specific and ubiquitous knockout of Wnt5a

Mice carrying two conditional alleles of Wnt5a (*Wnt5a$^{fl/fl}$* mice, kindly provided by Takahashi N., Nagano, Japan) (30) were crossbred with either *Col1a2/CreER* mice (31) or ubiquitin C (*Ubc*)-*CreER*-transgenic mice (The Jackson Lab, Bar Harbor, ME, USA) (32) to generate *Col1a2/CreER; Wnt5a$^{fl/fl}$* mice with inducible fibroblast-specific Cre activity (Wnt5a-fib-iKO) or *Ubc/Cre-ERT2; Wnt5a$^{fl/fl}$* with inducible Cre activity in all cell types (Wnt5a-ubc-iKO) (33, 34). Cre-mediated recombination was induced by repeated i.p. injections of tamoxifen (2 mg/ml) over five consecutive days. Control groups were injected with corn oil.

Mouse models of experimental fibrosis

Bleomycin-induced skin fibrosis was induced by local injections of bleomycin (2.5 mg/kg) every other day (3). Subcutaneous injections of 0.9 % NaCl served as control. In experiments with Wnt5a-fib-iKO mice or Wnt5a-ubc-iKO mice, a preventive setting was chosen with

induction of recombination before the first injection of bleomycin and the outcome was evaluated after four weeks.

Murine scl cGvHD was induced using the LP/J ($H\text{-}2^b$) C57/Bl6 ($H\text{-}2^b$) minor HLA mismatch model (35). *Col1a2/CreER; Wnt5a$^{fl/fl}$* C57BL/6 mice were transplanted after sublethal irradiation with 700 cGy with 5×10^7 splenocytes and 5×10^6 bone marrow cells from LP/J donor mice (The Jackson Laboratory). Control mice consisted of mice with transplantation of syngeneic splenocytes. Mice were monitored daily for changes in mobility, activity, consistency of the stool and changes of weight as clinical signs of cGvHD. The severity of cutaneous cGvHD was scored as described (36, 37). Mice were sacrificed after 42 days.

Histological analysis

Formalin-fixed, paraffin-embedded sections were stained with hematoxylin and eosin (HE). For direct visualization of collagen fibers, sections were stained with Sirius Red or Trichrome (Sigma-Aldrich). All histological images were captured using a Nikon Eclipse 80i microscope (Nikon). The dermal thickness was analyzed on HE-stained skin sections at four different sites in each mouse in a blinded manner (38). The collagen covered area in lung sections was determined by ImageJ. The severity of the histological changes of the lungs was analyzed using the Ashcroft score as described (39, 40).

Statistical Analysis

All in vitro and in vivo data are presented as median with interquartile range (IQR) with data representing individual data points. The statistical significance was determined by two-tailed Mann-Whitney *U*-test if two groups were compared, or one-way ANOVA with Tukey's multiple comparison test in case of more than two comparisons or two-way ANOVA with Bonferroni's multiple comparison test in case of multiple groups comparisons. In a subset of experiments, the mean values of the control groups were set to 1. All other values were

expressed as fold changes compared with the respective controls used as ‘comparison mean values’. P-values less than 0.05 were considered as significant differences.

Supplementary references

1. van den Hoogen F, Khanna D, Fransen J, Johnson SR, Baron M, Tyndall A, et al. 2013 classification criteria for systemic sclerosis: an American college of rheumatology/European league against rheumatism collaborative initiative. *Annals of the rheumatic diseases.* 2013;72(11):1747-55.
2. Jungel A, Distler JH, Kurowska-Stolarska M, Seemayer CA, Seibl R, Forster A, et al. Expression of interleukin-21 receptor, but not interleukin-21, in synovial fibroblasts and synovial macrophages of patients with rheumatoid arthritis. *Arthritis and rheumatism.* 2004;50(5):1468-76.
3. Dees C, Akhmetshina A, Zerr P, Reich N, Palumbo K, Horn A, et al. Platelet-derived serotonin links vascular disease and tissue fibrosis. *J Exp Med.* 2011;208(5):961-72.
4. Rossi A, Appelt-Menzel A, Kurdyn S, Walles H, and Groeber F. Generation of a three-dimensional full thickness skin equivalent and automated wounding. *J Vis Exp.* 2015(96).
5. Kah D, Durrbeck C, Schneider W, Fabry B, and Gerum RC. High-Force Magnetic Tweezers with Hysteresis-Free Force Feedback. *Biophys J.* 2020;119(1):15-23.
6. Sporrer M, Kah D, Gerum RC, Reischl B, Huraskin D, Dessalles CA, et al. The desmin mutation R349P increases contractility and fragility of stem cell-generated muscle micro-tissues. *Neuropathol Appl Neurobiol.* 2021.
7. Gerum RC, Richter S, Fabry B, and Zitterbart DP. ClickPoints: an expandable toolbox for scientific image annotation and analysis. *Methods Ecol Evol.* 2017;8(6):750-6.
8. Huang J, Maier C, Zhang Y, Soare A, Dees C, Beyer C, et al. Nintedanib macrophage activation and ameliorates vascular and fibrotic manifestations in the Fra2 mouse model of systemic sclerosis. *Annals of the rheumatic diseases.* 2017.
9. Chen CW, Beyer C, Liu J, Maier C, Li C, Trinh-Minh T, et al. Pharmacological inhibition of porcupine induces regression of experimental skin fibrosis by targeting Wnt signalling. *Ann Rheum Dis.* 2017;76(4):773-8.
10. Dees C, Potter S, Zhang Y, Bergmann C, Zhou X, Luber M, et al. TGFbeta-induced epigenetic deregulation of SOCS3 facilitates STAT3-signaling to promote fibrosis. *J Clin Invest.* 2020.
11. Yu G, Wang LG, Han Y, and He QY. clusterProfiler: an R package for comparing biological themes among gene clusters. *OMICS.* 2012;16(5):284-7.
12. Akhmetshina A, Palumbo K, Dees C, Bergmann C, Venalis P, Zerr P, et al. Activation of canonical Wnt signalling is required for TGF-beta-mediated fibrosis. *Nat Commun.* 2012;3:735.
13. Zerr P, Vollath S, Palumbo-Zerr K, Tomcik M, Huang J, Distler A, et al. Vitamin D receptor regulates TGF-beta signalling in systemic sclerosis. *Annals of the rheumatic diseases.* 2015;74(3):e20.
14. Bindels DS, Haarbosch L, van Weeren L, Postma M, Wiese KE, Mastop M, et al. mScarlet: a bright monomeric red fluorescent protein for cellular imaging. *Nat Methods.* 2017;14(1):53-6.
15. Abe M, Harpel JG, Metz CN, Nunes I, Loskutoff DJ, and Rifkin DB. An assay for transforming growth factor-beta using cells transfected with a plasminogen activator inhibitor-1 promoter-luciferase construct. *Analytical biochemistry.* 1994;216(2):276-84.
16. Khan SA, Joyce J, and Tsuda T. Quantification of active and total transforming growth factor-beta levels in serum and solid organ tissues by bioassay. *BMC research notes.* 2012;5:636.

17. Nuchel J, Ghatak S, Zuk AV, Illerhaus A, Morgelin M, Schonborn K, et al. TGFB1 is secreted through an unconventional pathway dependent on the autophagic machinery and cytoskeletal regulators. *Autophagy.* 2018;14(3):465-86.
18. Cluzel C, Saltel F, Lussi J, Paulhe F, Imhof BA, and Wehrle-Haller B. The mechanisms and dynamics of (alpha)v(beta)3 integrin clustering in living cells. *J Cell Biol.* 2005;171(2):383-92.
19. Branis J, Pataki C, Sporrer M, Gerum RC, Mainka A, Cermak V, et al. The role of focal adhesion anchoring domains of CAS in mechanotransduction. *Sci Rep.* 2017;7:46233.
20. Lagares D, Santos A, Grasberger PE, Liu F, Probst CK, Rahimi RA, et al. Targeted apoptosis of myofibroblasts with the BH3 mimetic ABT-263 reverses established fibrosis. *Sci Transl Med.* 2017;9(420).
21. Balaban NQ, Schwarz US, Riveline D, Goichberg P, Tzur G, Sabanay I, et al. Force and focal adhesion assembly: a close relationship studied using elastic micropatterned substrates. *Nat Cell Biol.* 2001;3(5):466-72.
22. Schindelin J, Arganda-Carreras I, Frise E, Kaynig V, Longair M, Pietzsch T, et al. Fiji: an open-source platform for biological-image analysis. *Nat Methods.* 2012;9(7):676-82.
23. Wang R, Li QF, Anfinogenova Y, and Tang DD. Dissociation of Crk-associated substrate from the vimentin network is regulated by p21-activated kinase on ACh activation of airway smooth muscle. *Am J Physiol Lung Cell Mol Physiol.* 2007;292(1):L240-8.
24. Bonakdar N, Gerum R, Kuhn M, Sporrer M, Lippert A, Schneider W, et al. Mechanical plasticity of cells. *Nat Mater.* 2016;15(10):1090-4.
25. Reich N, Tomcik M, Zerr P, Lang V, Dees C, Avouac J, et al. Jun N-terminal kinase as a potential molecular target for prevention and treatment of dermal fibrosis. *Annals of the rheumatic diseases.* 2012;71(5):737-45.
26. Shimizu Y, Dobashi K, Iizuka K, Horie T, Suzuki K, Tukagoshi H, et al. Contribution of small GTPase Rho and its target protein rock in a murine model of lung fibrosis. *Am J Respir Crit Care Med.* 2001;163(1):210-7.
27. Yadav H, Devalaraja S, Chung ST, and Rane SG. TGF-beta1/Smad3 Pathway Targets PP2A-AMPK-FoxO1 Signaling to Regulate Hepatic Gluconeogenesis. *J Biol Chem.* 2017;292(8):3420-32.
28. Bruijns RH, and Bult H. Effects of local cytochalasin D delivery on smooth muscle cell migration and on collar-induced intimal hyperplasia in the rabbit carotid artery. *Br J Pharmacol.* 2001;134(3):473-83.
29. Ulmasov B, Neuschwander-Tetri BA, Lai J, Monastyrskiy V, Bhat T, Yates MP, et al. Inhibitors of Arg-Gly-Asp-Binding Integrins Reduce Development of Pancreatic Fibrosis in Mice. *Cell Mol Gastroenterol Hepatol.* 2016;2(4):499-518.
30. Maeda K, Kobayashi Y, Udagawa N, Uehara S, Ishihara A, Mizoguchi T, et al. Wnt5a-Ror2 signaling between osteoblast-lineage cells and osteoclast precursors enhances osteoclastogenesis. *Nature medicine.* 2012;18(3):405-12.
31. Beyer C, Schramm A, Akhmetshina A, Dees C, Kireva T, Gelse K, et al. beta-catenin is a central mediator of pro-fibrotic Wnt signaling in systemic sclerosis. *Ann Rheum Dis.* 2012;71(5):761-7.
32. Ruzankina Y, Pinzon-Guzman C, Asare A, Ong T, Pontano L, Cotsarelis G, et al. Deletion of the developmentally essential gene ATR in adult mice leads to age-related phenotypes and stem cell loss. *Cell stem cell.* 2007;1(1):113-26.
33. Wendling O, Bornert JM, Chambon P, and Metzger D. Efficient temporally-controlled targeted mutagenesis in smooth muscle cells of the adult mouse. *Genesis (New York, NY : 2000).* 2009;47(1):14-8.

34. Sonnylal S, Denton CP, Zheng B, Keene DR, He R, Adams HP, et al. Postnatal induction of transforming growth factor beta signaling in fibroblasts of mice recapitulates clinical, histologic, and biochemical features of scleroderma. *Arthritis and rheumatism.* 2007;56(1):334-44.
35. Schultz KR, Bader S, Paquet J, and Li W. Chloroquine treatment affects T-cell priming to minor histocompatibility antigens and graft-versus-host disease. *Blood.* 1995;86(11):4344-52.
36. Huu DL, Matsushita T, Jin G, Hamaguchi Y, Hasegawa M, Takehara K, et al. FTY720 ameliorates murine sclerodermatous chronic graft-versus-host disease by promoting expansion of splenic regulatory cells and inhibiting immune cell infiltration into skin. *Arthritis and rheumatism.* 2013;65(6):1624-35.
37. Soare A, Ramming A, Avouac J, and Distler JH. Updates on animal models of systemic sclerosis. *Jsrd.* 2016;1(3):266-76.
38. Palumbo-Zerr K, Zerr P, Distler A, Fliehr J, Mancuso R, Huang J, et al. Orphan nuclear receptor NR4A1 regulates transforming growth factor-beta signaling and fibrosis. *Nat Med.* 2015;21(2):150-8.
39. Ashcroft T, Simpson JM, and Timbrell V. Simple method of estimating severity of pulmonary fibrosis on a numerical scale. *Journal of clinical pathology.* 1988;41(4):467-70.
40. Hubner RH, Gitter W, El Mokhtari NE, Mathiak M, Both M, Bolte H, et al. Standardized quantification of pulmonary fibrosis in histological samples. *BioTechniques.* 2008;44(4):507-11, 14-7.
41. Christmann RB, Wooten A, Sampaio-Barros P, Borges CL, Carvalho CR, Kairalla RA, et al. miR-155 in the progression of lung fibrosis in systemic sclerosis. *Arthritis Res Ther.* 2016;18(1):155.
42. Renaud L, da Silveira WA, Takamura N, Hardiman G, and Feghali-Bostwick C. Prominence of IL6, IGF, TLR, and Bioenergetics Pathway Perturbation in Lung Tissues of Scleroderma Patients With Pulmonary Fibrosis. *Front Immunol.* 2020;11:383.
43. Taroni JN, Greene CS, Martyanov V, Wood TA, Christmann RB, Farber HW, et al. A novel multi-network approach reveals tissue-specific cellular modulators of fibrosis in systemic sclerosis. *Genome Med.* 2017;9(1):27.
44. Zhang Y, Shen L, Dreissigacker K, Zhu H, Trinh-Minh T, Meng X, et al. Targeting of canonical WNT signaling ameliorates experimental sclerodermatous chronic graft-versus-host disease. *Blood.* 2021;137(17):2403-16.

Supplementary Table 1: Constructs for gene regulation

Plasmid / siRNA	Source
SignalSilence© SAPK/JNK siRNA	Cell signaling Technology
Ras homolog family member A siRNA (RHOA, s759)	Thermo Fisher scientific
TOP Flash	Millipore Corporation
FOP Flash	Millipore Corporation
β-galactosidase reporter vector	Sigma-Aldrich
SMAD binding element (SBE) AAV.	Thermo Fisher scientific
LPA/FLAG-TGFβ	Dr, Markus Plomann & Dr. Beate Eckes (17)
Integrin alpha V siRNA (ITGAV, s7547)	Thermo Fisher scientific
pLifeAct_mScarlet_N1	Optical Imaging Centre Erlangen
Vimentin-Chromobody TagGFP	ChromoTek
Non-targeting siRNAs	Life Technologies
pcDNA3.1	Thermo Fisher Scientific

Supplementary Table 2: Real-time PCR primers

Primer	Sequence
β-actin forward	5’-GACACCTCGCGGGCTCTGC-3’
β-actin reverse	5’-CGCCAGGCCTCCTGGAAACG - 3’
WNT4 forward	5’-CAGAGGCAGGTGCAGATGT-3’
WNT4 reverse	5’-ACCGAGTCCATGACTTCCAG - 3’
WNT5A forward	5’- CCTTCGCCCAGGTTGTAAT-3’
WNT5A reverse	5’- CATACCTAGCGACCACCAAGA-3’

WNT5B forward	5’-CTGTGCCAATTGTACCAGGA-3’
WNT5B reverse	5’-TTGGCTCCCTCCCTTATGTA - 3’
WNT11 forward	5’-TTTTCCGATGCTCCTATGAAG-3’
WNT11 reverse	5’-TTATTGGCTTGGGATCCTGT - 3’
WNT16 forward	5’-TGAAAGCATGACTGATGTCCA-3’
WNT16 reverse	5’-AGGCTGGATGGAGTGGTTACT - 3’
COL1A1 forward	5’-ACGAAGACATCCCACCAATC-3’
COL1A1 reverse	5’-ATGGTACCTGAGGCCGTTC-3’
ACTA2 forward	5’-AAGAGGAATCCTGACCCTGAA-3’
ACTA2 reverse	5’-TGGTGATGATGCCATGTTCT-3’
AXIN2 forward	5’- CATGACGGACAGCAGTGTAGA-3’
AXIN2 reverse	5’- CATGACGGACAGCAGTGTAGA-3’
PAI-1 forward	5’-TCATTGCTGCCCCTTATGA-3’
PAI-1 reverse	5’-GTTGGTGAGGGCAGAGAGAG-3’
CTGF forward	5’-AACTCACACAACAACTCTTCCCCGC3’
CTGF reverse	5’-GAGTCGCACTGGCTGTCTCCTCT-3’
TGFb1 forward	5’-AGGGGAAATTGAGGGCTTT -3’
TGFb1 reverse	5’-TGCTGTCACAGGAGCAGTG -3’
RHOA forward	5’-TGGGGTGGGCAGTTTTGAA -3’
RHOA reverse	5’-ATGAACTTGGGCTTTTCTGGTTG -3’
ITGAV forward	5’-TTGTGGGGTCCTTTGGTGTG -3’
ITGAV reverse	5’-TTTTCCAGAAGCATTGAGGCAG -3’

Supplementary Table 3: Antibody list

**Antibody**	**Source**

Polyclonal rabbit anti-Wnt5a (ab72583)	Abcam
WNT5 (C27E8) Rabbit mAb (#2530)	Cell signaling Technology
Monoclonal mouse anti-Prolyl 4-hydroxylase subunit beta (P4H)	Origene, Rockville, MD, USA
Monoclonal mouse anti-Vimentin (ab92547)	Abcam
DAPI (sc-3598)	Santa Cruz Biotechnology
Monoclonal mouse anti-actin, α-Smooth Muscle (A5224)	Sigma-Aldrich
Rhodamine phalloidin (R415)	Thermo Fisher Scientific
Monoclonal rabbit anti-Collagen I (EPR7785) (ab138492)	Abcam
Polyclonal rabbit anti-JNK1+JNK2 (phospho T183 + Y185) (ab4821)	Abcam
Polyclonal rabbit anti-SAPK/JNK (#9525)	Cell signaling Technology
Monoclonal mouse anti-beta catenin (A5441)	Sigma-Aldrich
Monoclonal mouse anti-p-c-Jun (KM-1) (sc-822)	Santa Cruz Biotechnology
Monoclonal rabbit anti-c-Jun (E254) (ab32137)	Abcam
Monoclonal rabbit anti-p-CaMKII (Thr286) (D21E4)	Cell signaling Technology
Monoclonal mouse anti-CaM Kinase II (611292)	BD Biosciences, San Jose, CA, USA
Polyclonal rabbit anti-β-catenin (ab2365)	Abcam
Polyclonal rabbit anti-Lamin A/C (#2032)	Cell signaling Technology
Polyclonal rabbit anti-Vimentin (R28) (#3932)	Cell signaling Technology
Monoclonal rabbit anti-Smad3 (phospho S423 + S425) (ab52903)	Abcam
Monoclonal rabbit anti-Smad3(C67H9)	Cell signaling Technology
Monoclonal mouse anti-TGFbeta 1,2,3 (1D11)	R&D Systems

Human LAP (TGF-beta1) (MAB2461)	R&D Systems
Monoclonal anti-FLAG M2 antibody (F3165)	Sigma-Aldrich
TGF beta-1,2,3 Monoclonal Antibody (eBioTB2F)	Thermo Fisher Scientific
Recombinant Anti-Integrin alpha V antibody [EPR16800] (Alexa Fluor® 594) (ab207286)	Abcam
P-PAXILLIN (S178) (ab193677)	Abcam
PAXILLIN monoclonal antibody (5H11)	Millipore Corporation
TALIN (Ser-425), phosphor specific (TP-4171)	ECM Bioscience, Versailles, KY, USA
Anti-TALIN1 antibody (ab137843)	Abcam
Alpha Tubulin monoclonal antibody (14-4502-82)	Thermo Fisher Scientific
SiR-tubulin	Spirochrome
Anti-Pan Actin Rabbit polyclonal (#AAN01-A)	Cytoskeleton Inc.
Alexa Fluor 488 goat anti-mouse IgG	Thermo Fisher Scientific
Alexa Fluor 594 goat anti-rabbit IgG	Thermo Fisher Scientific
Alexa Fluor™ 647 conjugated WGA	Thermo Fisher Scientific
Rabbit IgG	Santa Cruz

Supplementary Table 4: WNT5A DEGs

Gene symbol	logFC	PValue
CCN2	1.6756	4.97E-09
HAPLN1	3.0437	1.39E-08
ASPN	4.0423	2.38E-08
TPM1	1.0329	1.59E-07
KLK4	4.8826	2.66E-07
NIPAL4	3.6786	5.84E-07
COL10A1	4.2091	6.15E-07
CLTCL1	0.7714	1.06E-06
TP53I3	0.7756	1.12E-06
UCN2	1.5422	1.22E-06

Gene symbol	logFC	PValue
FSTL3	0.8607	1.35E-06
SDC1	1.0342	1.39E-06
ID1	2.6243	1.70E-06
ALDH1B1	0.9346	3.05E-06
FN1	1.0722	3.10E-06
KCNH1	2.2039	3.60E-06
DACT1	3.0632	3.92E-06
FNDC1	1.9706	4.30E-06
LUZP2	3.3761	4.54E-06
PCDH19	2.8494	4.66E-06

Gene symbol	logFC	PValue
SPECC1	0.9023	5.93E-06
XYLT1	0.7975	6.50E-06
KANK4	6.8830	6.76E-06
COL11A1	2.6199	7.45E-06
EDIL3	1.8086	8.38E-06
ACTA2	2.6100	1.08E-05
SMCO4	0.9404	1.34E-05
AMIGO2	1.6843	1.63E-05
PMEPA1	1.5323	1.77E-05
BGN	1.0839	1.97E-05
WFDC1	3.1221	1.98E-05
COL1A1	1.4451	2.12E-05
ELN	3.0375	2.14E-05
PPP1R14A	1.7874	2.17E-05
COL5A1	1.3288	2.50E-05
COL7A1	1.1347	2.72E-05
MICAL2	0.7425	3.18E-05
IGFBPL1	2.3640	4.08E-05
PPP1R13L	0.7206	4.80E-05
COL8A2	0.8268	5.29E-05
TSPAN2	1.5894	5.66E-05
PXDN	0.7564	5.98E-05
POSTN	2.4736	6.10E-05
KRT81	3.2180	7.42E-05
GPC4	0.6146	7.57E-05
PDLIM7	0.8159	8.06E-05
MFAP2	0.6679	9.64E-05
HHAT	1.0734	1.06E-04
STXBP6	-0.7946	1.08E-04
PALLD	0.7969	1.11E-04
MEP1B	-4.2565	1.25E-04
SORBS2	2.0947	1.26E-04
BCL11B	1.6793	1.31E-04
SRGN	-1.2215	1.33E-04
CCDC144NL-AS1	2.0341	1.34E-04
MYH9	0.5980	1.36E-04
EXTL1	1.3025	1.37E-04
LRRC15	1.8814	1.42E-04
GRK5	-0.5855	1.49E-04

Gene symbol	logFC	PValue
KCNK6	0.6736	1.69E-04
NUAK1	0.9019	1.85E-04
IGFBP3	2.2061	1.90E-04
SAMD11	1.2854	1.91E-04
MEX3B	0.8021	1.97E-04
DOK6	0.6541	2.17E-04
DIRC1	-1.6585	2.22E-04
LTBP2	1.0319	2.27E-04
SPARC	0.8823	2.37E-04
CORIN	1.8863	2.41E-04
CNN1	1.3917	2.45E-04
C5orf46	4.3925	2.64E-04
COMP	1.8369	3.03E-04
LDLRAD4	1.9724	3.06E-04
GADD45B	0.8980	3.27E-04
BTC	-2.8296	3.46E-04
DACT3	0.8581	3.51E-04
RFLNB	1.0048	3.60E-04
SNORD62B	2.0454	3.71E-04
PLPPR3	1.6296	3.77E-04
MFAP3L	0.9579	3.79E-04
KCNQ3	-1.2340	3.80E-04
EFHD1	1.5556	3.91E-04
PLA2G4A	-1.1114	4.36E-04
PRAG1	1.0209	4.47E-04
ITGA11	1.9214	4.71E-04
TPM2	0.6276	4.78E-04
BDKRB2	-0.8304	5.22E-04
INHBE	2.3585	5.27E-04
SHISA9	2.2649	5.49E-04
IQCA1	1.6220	5.49E-04
LINC01521	0.6685	5.73E-04
RALGAPA1P1	-1.0373	6.21E-04
NT5E	-0.5892	6.27E-04
TAS2R20	-2.3432	6.29E-04
COL4A2	0.8323	6.32E-04
NDUFA4L2	1.0710	6.46E-04
MEGF6	0.9310	6.66E-04
MINCR	1.1533	6.90E-04

Gene symbol	logFC	PValue
NKD2	2.1879	7.26E-04
ITGBL1	0.9326	7.44E-04
LINC00685	0.9427	8.08E-04
KPRP	-5.1341	8.15E-04
ADGRG1	-1.1286	8.17E-04
ADAM19	0.6673	8.22E-04
CNN2	0.6784	8.49E-04
PKP2	2.5177	8.53E-04
IRAK2	-0.6119	8.54E-04
CCL7	-1.0640	1.01E-03
VCAN	1.1650	1.02E-03
TP53TG1	-1.4331	1.03E-03
KAZALD1	0.7063	1.04E-03
ALDH1L2	0.6026	1.04E-03
GLI2	0.7586	1.05E-03
ID3	1.7260	1.06E-03
PCK2	0.6990	1.08E-03
KCNE4	0.7359	1.13E-03
LINC01503	0.8223	1.19E-03
TAGLN	1.4677	1.28E-03
BMF	1.1851	1.31E-03
CDH2	0.7161	1.37E-03
CBWD3	0.9766	1.39E-03
SERTAD4-AS1	1.5905	1.41E-03
FLNA	0.6214	1.42E-03
MYOCD	2.1920	1.42E-03
ENPP2	-1.0139	1.44E-03
IDO1	-2.3655	1.47E-03
MRVI1	0.8525	1.48E-03
CDH4	0.8489	1.49E-03
PODNL1	0.6134	1.51E-03
SNORD6	-2.9472	1.54E-03
LINC01293	-2.3888	1.55E-03
IL1RL1	-2.0629	1.59E-03
PIK3IP1	0.6662	1.63E-03
MIR503HG	0.8780	1.64E-03
MIR155HG	-1.1822	1.69E-03
COL4A1	1.0426	1.72E-03
FAM21EP	-1.1545	1.76E-03

Gene symbol	logFC	PValue
NRG1	0.7104	1.76E-03
FGF14	1.5234	1.77E-03
PLPP4	1.7696	1.82E-03
HMCN1	1.2257	1.84E-03
LINC01614	2.5753	1.92E-03
GJA5	1.6433	1.94E-03
ZSWIM4	0.6597	2.01E-03
ICAM5	1.3290	2.01E-03
PSMB10	-0.7662	2.16E-03
CASP17P	-1.3920	2.16E-03
BTBD11	1.5567	2.21E-03
TSPAN18	0.7785	2.22E-03
ANO1	2.3014	2.28E-03
LINC00271	-1.8357	2.30E-03
ACSL5	-1.1625	2.32E-03
MYO7B	1.2818	2.33E-03
TTC9	1.0997	2.35E-03
WNT7B	2.8371	2.43E-03
SOX9	1.0950	2.44E-03
NIM1K	-0.7886	2.44E-03
LIMS2	0.8379	2.48E-03
GLI1	1.9079	2.50E-03
LMOD1	0.9397	2.56E-03
KRT7	1.0105	2.61E-03
LINC01013	1.4255	2.61E-03
STARD4-AS1	0.6003	2.63E-03
SNRPGP10	-1.7904	2.64E-03
RHBDL1	0.7524	2.66E-03
SFMBT2	-2.5287	2.66E-03
NOTCH4	-0.8923	2.66E-03
STAC3	0.9881	2.68E-03
HPSE	-1.5682	2.83E-03
BHLHE40	0.8067	2.83E-03
ELOVL7	-1.8011	2.86E-03
LIPC	-0.7957	2.88E-03
PSAT1	1.1943	2.88E-03
PDE11A	1.4002	2.89E-03
TSPAN13	1.0203	2.94E-03
PDZD7	1.2622	2.95E-03

Gene symbol	logFC	PValue
KLHL30	2.3901	2.96E-03
ZGLP1	1.4100	2.99E-03
MAGED4	2.5872	3.04E-03
CSMD1	2.1847	3.14E-03
ZNF815P	0.8662	3.21E-03
EBF1	-0.6928	3.27E-03
MEDAG	-0.8530	3.28E-03
TRIB2	1.1583	3.29E-03
SLC7A5	1.2112	3.29E-03
TLE2	0.5846	3.37E-03
CCZ1	0.9933	3.40E-03
PPME1	1.2833	3.42E-03
CXCL6	-1.2840	3.48E-03
UCP2	1.2678	3.52E-03
KIF21A	0.6569	3.61E-03
GPR17	0.8733	3.66E-03
MT1G	-3.8089	3.74E-03
TAPBPL	-0.7461	3.77E-03
RIPOR2	-0.9699	3.83E-03
MYL9	0.7596	3.88E-03
AARD	2.0985	3.89E-03
MATN2	-0.7734	4.04E-03
ASPRV1	1.1413	4.06E-03
TNIP3	-0.9822	4.17E-03
CD36	-1.7989	4.18E-03
NBEAP1	2.2663	4.29E-03
EMC3-AS1	-0.8254	4.32E-03
OPCML	1.8204	4.74E-03
ZMYND15	-1.7035	4.81E-03
SLC1A4	0.6102	4.86E-03
APBA2	-0.8005	4.94E-03
TMEM171	-0.7600	5.02E-03
SDK2	0.8410	5.05E-03
ZC2HC1C	-1.0951	5.17E-03
ADIRF-AS1	0.6032	5.19E-03
DLGAP1	-1.3592	5.22E-03
AMZ1	0.8606	5.30E-03
PATL2	-0.9100	5.41E-03
JPH2	0.6356	5.55E-03

Gene symbol	logFC	PValue
NREP	0.6221	5.66E-03
COL24A1	0.6912	5.69E-03
PCDHGB3	0.6739	5.79E-03
CAPN6	2.0111	5.94E-03
LINC02593	0.6371	6.01E-03
ACTN2	-1.1112	6.04E-03
CCDC183-AS1	0.8690	6.18E-03
MAP4K1	2.0061	6.18E-03
SDCBPP3	-2.1411	6.20E-03
RDH5	-0.7374	6.29E-03
CENPV	0.6202	6.31E-03
DDIT4L	-1.0710	6.32E-03
MACROD2	0.9054	6.45E-03
LINC01094	-0.7878	6.62E-03
C7orf57	4.8429	6.74E-03
BDNF	0.6481	6.75E-03
APLP1	0.5996	6.76E-03
ACTA2-AS1	1.4978	6.81E-03
LINC01444	1.2290	6.98E-03
SYNDIG1	1.2250	7.08E-03
PLEKHA4	-0.7747	7.12E-03
HLA-E	-0.5887	7.29E-03
ZNF730	2.6497	7.31E-03
OPN3	0.6134	7.38E-03
TMEM217	-0.6919	7.39E-03
PINK1-AS	0.6807	7.40E-03
CCN3	-0.6812	7.41E-03
SORT1	0.6257	7.46E-03
TNFAIP6	-1.5128	7.51E-03
TYSND1	-0.6305	7.53E-03
DEGS2	1.7226	7.67E-03
FBXO32	0.5970	7.79E-03
PTX3	-1.3491	7.87E-03
CLIC4P3	-2.0660	7.90E-03
PTGFR	-0.5871	7.97E-03
PCAT6	0.7546	8.10E-03
TEX41	-1.0582	8.25E-03
BCL2L2-PABPN1	1.8125	8.34E-03

Gene symbol	logFC	PValue
IGFBP6	-0.8430	8.57E-03
TNFRSF1B	-0.6001	8.64E-03
TTYH3	0.6037	8.64E-03
LAP3	-1.2112	8.71E-03
RAET1E	-1.2412	8.77E-03
UNC5B	0.7450	8.78E-03
BATF2	-1.1110	9.02E-03
CLEC4A	-1.4313	9.06E-03
PADI1	2.2092	9.20E-03
RASD2	1.6736	9.22E-03
MIR503	1.3142	9.27E-03
KRT80	0.9670	9.45E-03
PCED1B	0.7926	9.54E-03
CLCA2	-1.1447	9.60E-03
TTC3P1	0.6731	9.64E-03
ASNS	0.9227	9.64E-03
PHGDH	0.7901	9.81E-03
HSP90B2P	-1.3352	9.81E-03
TNFSF18	-2.0835	9.83E-03
PAPPA2	2.2198	9.89E-03
SQOR	-0.5862	9.89E-03
RND3	-0.6658	9.93E-03
NKILA	0.7569	9.97E-03
ADH1B	-1.3265	1.01E-02
DRD1	2.2957	1.01E-02
MACC1	-1.9085	1.01E-02
GUCY1A2	-0.7457	1.03E-02
KCNMB2	1.5951	1.04E-02
C9orf170	-1.2978	1.04E-02
CADM1	0.6508	1.04E-02
NOX4	1.1666	1.05E-02
STON2	-1.0575	1.05E-02
TMEM100	-2.0726	1.05E-02
CSRP2	0.9032	1.06E-02
EGR2	0.6406	1.07E-02
HLA-DPA1	-0.9978	1.07E-02
HECW2	-0.7194	1.10E-02
PSG1	-1.4683	1.10E-02
NCAM1	0.7328	1.11E-02
EIF4EBP1	0.6293	1.12E-02
PRR15	1.7551	1.13E-02
SOX4	0.6102	1.14E-02
MPZ	1.2835	1.15E-02
GIMAP2	-0.6233	1.15E-02
ZFPM2	-1.8998	1.15E-02
RXFP1	-1.0182	1.15E-02
BHLHE22	-0.8617	1.17E-02
SPINT2	0.9199	1.18E-02
SERTAD4	1.6190	1.19E-02
HIST2H2BE	-0.8292	1.19E-02
KLHL35	0.9613	1.22E-02
CBSL	1.1640	1.22E-02
CDKN2B	-0.5994	1.25E-02
SPOCK1	0.6774	1.25E-02
LINC00323	1.4122	1.25E-02
CRISPLD2	0.8131	1.25E-02
PSG11	-1.9000	1.30E-02
CELSR1	1.2177	1.31E-02
GJA3	4.0386	1.31E-02
PYCR1	0.7590	1.32E-02
LOXL2	0.6531	1.32E-02
HLA-H	-0.7711	1.32E-02
TCN2	-0.7207	1.33E-02
ADCY2	1.7483	1.33E-02
ID4	0.8992	1.34E-02
RPL26P30	1.3073	1.35E-02
SEC22B2	-0.9538	1.37E-02
PRLR	-1.2300	1.38E-02
ENPP1	0.9639	1.39E-02
CDKL1	-0.6045	1.40E-02
DHRS11	0.7690	1.41E-02
ITGB8	-0.8336	1.45E-02
TOMM5	1.1709	1.45E-02
IL10RB-DT	-1.1564	1.47E-02
ADAM12	0.5962	1.47E-02
PTPRN2	-0.9597	1.48E-02
PCSK2	-1.2165	1.49E-02
SAMD9	-0.8681	1.49E-02

Gene symbol	logFC	PValue
BATF3	-1.0183	1.50E-02
P4HA3	0.7736	1.52E-02
ANXA2R	0.9055	1.52E-02
LY6K	-0.9298	1.52E-02
CTHRC1	0.9482	1.53E-02
GBP3	-0.7613	1.53E-02
MIR4653	-1.8723	1.55E-02
OXTR	1.1977	1.55E-02
AGPAT4-IT1	1.0245	1.56E-02
APOD	-0.6469	1.57E-02
NCOA7	-0.6540	1.57E-02
PHKG1	0.9071	1.57E-02
HECW1	0.6641	1.59E-02
PGF	-1.3788	1.66E-02
ATP2B1	-0.6377	1.68E-02
FAM53A	-0.6303	1.70E-02
SLC47A1	-0.6382	1.72E-02
BDKRB1	-1.1085	1.72E-02
KIF26B	0.6329	1.73E-02
SGCG	1.0554	1.73E-02
LINC02432	-1.4192	1.74E-02
AKR1C1	-0.8935	1.74E-02
ETV2	0.8331	1.75E-02
LINC01273	-1.5885	1.79E-02
SLC4A3	0.6514	1.80E-02
TPD52L1	1.2223	1.82E-02
NLGN3	-1.0603	1.82E-02
ZNF85	0.6478	1.82E-02
DIRAS3	-0.9452	1.83E-02
APOL3	-0.9743	1.84E-02
HR	0.8985	1.85E-02
FIBCD1	1.0020	1.87E-02
TMEM155	0.7897	1.87E-02
SMIM25	-0.7531	1.88E-02
SMURF2P1	0.5879	1.89E-02
ASB3	0.8442	1.89E-02
HERC5	-1.2703	1.89E-02
ATRNL1	1.0005	1.91E-02
THEMIS2	-0.7806	1.91E-02

Gene symbol	logFC	PValue
SLC2A5	-1.3002	1.92E-02
DCN	-0.6682	1.93E-02
MT1E	-0.9843	1.94E-02
KIF5C	-1.5026	1.95E-02
MIR25	1.0734	1.95E-02
CXCL11	-2.2813	1.96E-02
NALCN	0.9931	1.96E-02
CPN2	1.5461	1.96E-02
CLMN	0.9318	1.97E-02
MT1M	-1.0677	1.99E-02
TMEM204	0.6443	2.00E-02
ANKRD20A5P	0.8163	2.00E-02
DTNA	-1.0957	2.01E-02
ABCA6	-0.6013	2.03E-02
GAB3	-0.9242	2.05E-02
CHI3L2	-0.9983	2.07E-02
KCNAB3	0.7586	2.08E-02
GPD2	-0.8689	2.10E-02
TMC4	0.8233	2.10E-02
CLEC3B	-1.3878	2.11E-02
FYB1	-1.4663	2.11E-02
PRKCZ	1.1573	2.12E-02
RASGRP2	0.8520	2.13E-02
MT1F	-1.6012	2.14E-02
DPT	1.3059	2.14E-02
DUSP6	-0.5939	2.15E-02
MKX-AS1	0.9257	2.16E-02
CXCL1	-1.0569	2.16E-02
MBLAC1	0.5849	2.17E-02
ACTBP2	0.9246	2.17E-02
RAMP1	1.2087	2.18E-02
FTH1P20	-0.7699	2.21E-02
ULBP1	0.6256	2.22E-02
LRRC69	-0.9476	2.24E-02
SYBU	0.9344	2.26E-02
CTSS	-1.2069	2.26E-02
RNU6-30P	1.7045	2.28E-02
DNAAF1	1.5722	2.28E-02
TENM4	0.6642	2.29E-02

Gene symbol	logFC	PValue
NR0B1	-1.2524	2.30E-02
COL9A2	0.8659	2.31E-02
DNER	-2.5227	2.31E-02
HCK	-1.4906	2.32E-02
IFI27	-0.7623	2.32E-02
ANKRD55	-1.6478	2.32E-02
DLEU1	0.8269	2.33E-02
SECTM1	-0.7268	2.33E-02
CARMN	0.6835	2.33E-02
ANKRD39	0.6481	2.35E-02
HELLPAR	1.3937	2.35E-02
TLR3	-1.2258	2.36E-02
LINC02454	-0.9352	2.39E-02
LYPD5	-1.0313	2.41E-02
RPL5P34	0.5866	2.41E-02
NOTUM	1.1784	2.43E-02
GCH1	-1.3575	2.43E-02
MME	-0.6137	2.45E-02
DOCK2	0.5896	2.46E-02
MYO5B	3.6064	2.46E-02
CHEK2	-0.5971	2.46E-02
NDUFA6-DT	0.6745	2.47E-02
TNXB	-1.2218	2.50E-02
VLDLR-AS1	1.6091	2.51E-02
IFITM1	-0.8777	2.52E-02
CLDN1	-1.1771	2.54E-02
UBE2Q2P2	0.8602	2.55E-02
KLHL3	0.7504	2.55E-02
LINC01711	1.6430	2.57E-02
CCDC65	0.7226	2.57E-02
LBX2	0.6111	2.58E-02
MANSC1	-0.6524	2.61E-02
POLR2I	0.8258	2.61E-02
CELSR2	0.7049	2.62E-02
MT2A	-0.7608	2.63E-02
CXCL16	-0.9051	2.64E-02
ACTC1	1.7296	2.66E-02
COL6A3	0.6824	2.66E-02
CXCL9	-1.6088	2.67E-02

Gene symbol	logFC	PValue
HILS1	2.0650	2.68E-02
IGF2	0.7736	2.69E-02
HELZ2	-0.6143	2.70E-02
LINC01705	1.9209	2.70E-02
CES1	0.9345	2.71E-02
COX20P1	1.0437	2.71E-02
CEP170P1	0.9716	2.72E-02
OASL	-1.7304	2.73E-02
DNMT3B	0.6678	2.74E-02
NEURL3	-1.9225	2.75E-02
FGF13	-0.7585	2.75E-02
SNHG9	1.0235	2.75E-02
INHBA	1.2276	2.75E-02
MRPL23-AS1	0.8319	2.76E-02
IL15RA	-0.9049	2.76E-02
IL18BP	-1.5637	2.76E-02
SLC38A4	1.2293	2.77E-02
RBM5-AS1	-1.5130	2.78E-02
FAM72A	-0.9638	2.79E-02
SRPK3	0.7107	2.79E-02
C2	-0.8414	2.81E-02
CTSK	-0.5982	2.83E-02
NKX3-1	-0.7941	2.84E-02
SSPO	0.9718	2.85E-02
CCL20	-2.1011	2.86E-02
CXCL5	-1.2841	2.88E-02
LINC01638	2.3135	2.88E-02
SERPINB2	-0.8607	2.88E-02
EXOC3L1	-1.0116	2.90E-02
GVINP1	-1.1418	2.90E-02
NAMPT	-0.7654	2.90E-02
NADK2-AS1	1.2837	2.92E-02
SBSN	-0.7578	2.93E-02
CYP4F35P	1.1231	2.94E-02
TNFAIP8L3	-0.8035	2.95E-02
HERC6	-0.9128	2.96E-02
MT2P1	-0.9815	2.97E-02
CPM	0.7249	2.99E-02
03-Mar	-0.6074	2.99E-02

Gene symbol	logFC	PValue
CD38	-2.2581	3.00E-02
DHDH	-4.5414	3.00E-02
CD37	0.7079	3.01E-02
APOL1	-0.9332	3.02E-02
TMED6	-1.3399	3.03E-02
GRID1	0.9629	3.03E-02
RASAL2-AS1	-0.6119	3.04E-02
CHRNA9	1.8233	3.05E-02
SULT1A3	1.4940	3.05E-02
RSPH4A	-1.0464	3.11E-02
MDFI	0.6306	3.11E-02
IKBKGP1	1.4551	3.11E-02
PNPT1	-0.7041	3.13E-02
ITIH4	-2.1015	3.17E-02
RPS2P55	1.1047	3.17E-02
UNC93B1	-0.5941	3.18E-02
RAB20	-0.6775	3.22E-02
YTHDF3-AS1	0.9285	3.23E-02
PTGS2	-0.9793	3.24E-02
DAAM2	0.5971	3.24E-02
ADM2	1.0052	3.24E-02
GBP5	-1.2060	3.24E-02
LINC01347	-1.3704	3.26E-02
SYCP3	-1.2455	3.28E-02
DBP	0.7349	3.30E-02
IQCN	0.6018	3.30E-02
DGKI	1.0223	3.31E-02
PRRG3	0.7873	3.32E-02
DIO2	0.9352	3.32E-02
GPR160	-1.0739	3.32E-02
GCA	-1.0998	3.34E-02
WASH4P	-0.6439	3.35E-02
ISG20	-1.8468	3.37E-02
PLCH1	1.8010	3.37E-02
DGCR11	-0.6290	3.39E-02
FGF14-AS2	-1.3642	3.41E-02
CDC42BPG	-0.7707	3.44E-02
MPP4	0.8287	3.47E-02
FBXO39	-1.0275	3.48E-02

Gene symbol	logFC	PValue
HUNK	0.9227	3.52E-02
MYPN	-0.9854	3.54E-02
PSG9	-0.8330	3.54E-02
FER1L6	-0.8943	3.54E-02
RIMS1	1.0276	3.56E-02
MLKL	-0.6643	3.57E-02
AFF3	0.9086	3.57E-02
ANGPTL1	-1.3456	3.58E-02
ELF3-AS1	-2.1556	3.60E-02
PDGFRL	-0.8558	3.61E-02
AOX1	-0.7300	3.62E-02
FEM1AP2	1.1678	3.62E-02
LRATD2	0.6416	3.66E-02
PSMB9	-0.6851	3.67E-02
CXCL2	-0.9446	3.69E-02
PALM2	0.6155	3.69E-02
TNFSF13B	-1.8887	3.72E-02
EXOC3L4	-1.8662	3.73E-02
SPDL1	0.6874	3.74E-02
OGFR-AS1	-0.6783	3.76E-02
CES1P1	1.7854	3.78E-02
DCST1-AS1	1.3319	3.78E-02
SRRM2-AS1	-0.9323	3.80E-02
CXCL10	-2.2845	3.80E-02
NCKAP5	1.0514	3.83E-02
TMEM140	-0.5869	3.85E-02
FBXO6	-0.6220	3.86E-02
WDR86	-1.3335	3.86E-02
SYT2	1.3145	3.87E-02
GBP1P1	-1.4375	3.88E-02
MTSS2	0.6125	3.88E-02
RAPGEF5	-0.7433	3.90E-02
HSPB7	0.9283	3.91E-02
RGS9	1.0386	3.94E-02
CFB	-1.0419	3.94E-02
IL12RB1	-1.2864	3.94E-02
SLC9A7P1	0.9188	3.95E-02
LAMC2	-0.7879	3.97E-02
CCDC194	-0.9982	3.98E-02

Gene symbol	logFC	PValue
CALCRL	1.4374	3.99E-02
IFIT1	-1.0854	4.01E-02
ZNF732	1.6643	4.02E-02
ANKRD24	0.9714	4.04E-02
APELA	1.5243	4.05E-02
CHADL	0.9303	4.06E-02
TGFBI	0.7460	4.06E-02
SLC7A9	-1.4448	4.08E-02
IRF1	-0.6954	4.09E-02
HIST1H4H	-1.0353	4.09E-02
NPIPA2	-2.6620	4.10E-02
IFIT3	-1.0701	4.15E-02
TF	-1.4815	4.19E-02
CYP11A1	-1.2602	4.21E-02
GPAM	0.8250	4.25E-02
BST2	-0.6716	4.26E-02
EEF1A2	0.6971	4.30E-02
HOXC-AS3	0.6313	4.31E-02
GALNT16	-0.7181	4.31E-02
NR4A1	0.7435	4.32E-02
TCTE1	2.5775	4.32E-02
EFEMP1	-0.6781	4.34E-02
IFIT2	-1.8473	4.34E-02
KDR	-0.8985	4.37E-02
GVQW3	0.5958	4.40E-02
COL4A4	0.9003	4.42E-02
ELFN2	1.3019	4.42E-02
CYP3A5	-1.1290	4.42E-02
UBE2FP3	-1.0502	4.43E-02
WNT10B	1.2699	4.43E-02
CBX2	0.7706	4.43E-02
RSPO3	-0.8945	4.45E-02
ABI3BP	-1.1381	4.45E-02
PLAAT4	-1.0979	4.46E-02
MX2	-0.9142	4.47E-02
PLXDC2	0.9937	4.50E-02

Gene symbol	logFC	PValue
RN7SL124P	-2.2181	4.51E-02
LINC01937	-1.1761	4.52E-02
TP63	-1.6908	4.54E-02
FCRLA	-1.4468	4.57E-02
CLIC2	-0.6019	4.58E-02
PTCHD4	-0.6924	4.59E-02
NOD2	-1.1101	4.59E-02
ADAMDEC1	-1.0345	4.61E-02
HIST2H2BA	-0.8565	4.63E-02
IL22RA1	-0.9877	4.65E-02
FAM20A	-0.8371	4.65E-02
SAMD9L	-0.6906	4.67E-02
U2AF1	-1.8340	4.67E-02
NT5DC2	0.6412	4.70E-02
DDX60L	-0.6519	4.70E-02
DRICH1	-1.3465	4.70E-02
SLC47A2	-1.1259	4.70E-02
NMNAT2	-1.2686	4.72E-02
PTGER3	0.9536	4.75E-02
LMF1-AS1	0.8878	4.75E-02
LCE2A	-2.4666	4.81E-02
C2CD3	0.6589	4.82E-02
AQP1	1.0353	4.83E-02
PPP1R9A	-1.1186	4.84E-02
SHROOM2	1.0130	4.88E-02
PCDH7	0.8524	4.88E-02
DDX58	-0.8879	4.91E-02
ABCG4	0.6881	4.92E-02
CYP46A1	0.6489	4.94E-02
LRRC17	1.2789	4.95E-02
HOXD3	-1.1170	4.97E-02
MUSK	-1.4137	4.98E-02
IL33	-2.1699	4.99E-02
NINJ2	-0.6261	5.00E-02

SUPPLEMENTARY FIGURES AND VIDEOS

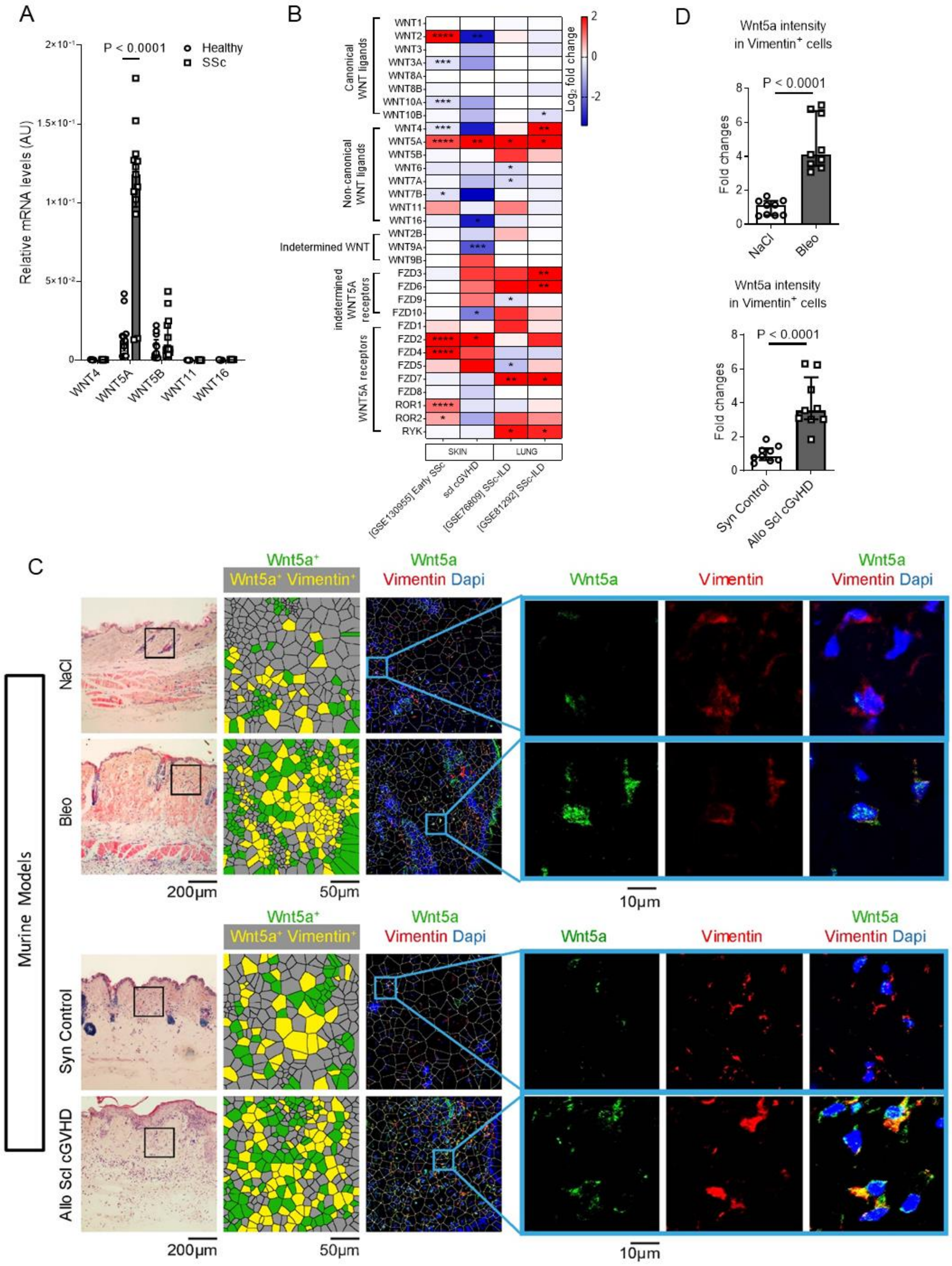

A
B
C
D
P < 0.0001
Healthy
SSc
Relative mRNA levels (AU)
WNT4
WNT5A
WNT5B
WNT11
WNT16
Canonical WNT ligands
Non-canonical WNT ligands
Indetermined WNT
indetermined WNT5A receptors
WNT5A receptors
Log2 fold change
SKIN
LUNG
[GSE130955] Early SSc
scl cGVHD
[GSE76809] SSc-ILD
[GSE81292] SSc-ILD
Wnt5a intensity in Vimentin+ cells
Fold changes
NaCl
Bleo
Syn Control
Allo Scl cGVHD
Murine Models
Wnt5a+
Wnt5a+ Vimentin+
Wnt5a
Vimentin
Dapi
200µm
50µm
10µm

Supplementary Figure 1: WNT5A is expressed at increased levels in human fibrotic diseases such as early SSc, SSc-ILD and Scl cGvHD, and murine models of fibrosis.

(**A**) Absolute mRNA levels of all non-canonical WNT proteins in the skin of SSc patients and matched healthy individuals (n = 13 each group). (**B**) Screening of WNT ligands and their receptors in different cohorts of fibrotic diseases (41-43). Data on the Scl cGVHD cohort (44)were provided by Dr. Yun Zhang . (**C**) Representative IF stainings for Wnt5a (green), Vimentin (red) and Dapi (blue) in skin of mice with bleomycin-induced skin fibrosis (n = 9) and Scl cGvHD (n = 9) as compared to the respective non-fibrotic controls (n = 9) along with Voronoi tessellation and (**D**) quantification of the Wnt5a staining. Results are shown as median ± IQR with data representing individual data points. The statistical significance was determined by two-tailed Mann-Whitney *U*-test, if two groups were compared, or one-way ANOVA with Tukey's multiple comparison test in case of more than two comparisons. In figure B, * indicates significant differences of fibrotic group compared to their control. *: FDR < 0.05; **: FDR < 0.01; ***: FDR < 0.001; and ****: FDR < 0.0001. FDR: False-Discovery-Rate. Scl: Sclerodermatous. cGVHD: chronic Graft-versus-Host-Disease. SSc-ILD indicates SSc-associated interstitial lung disease.

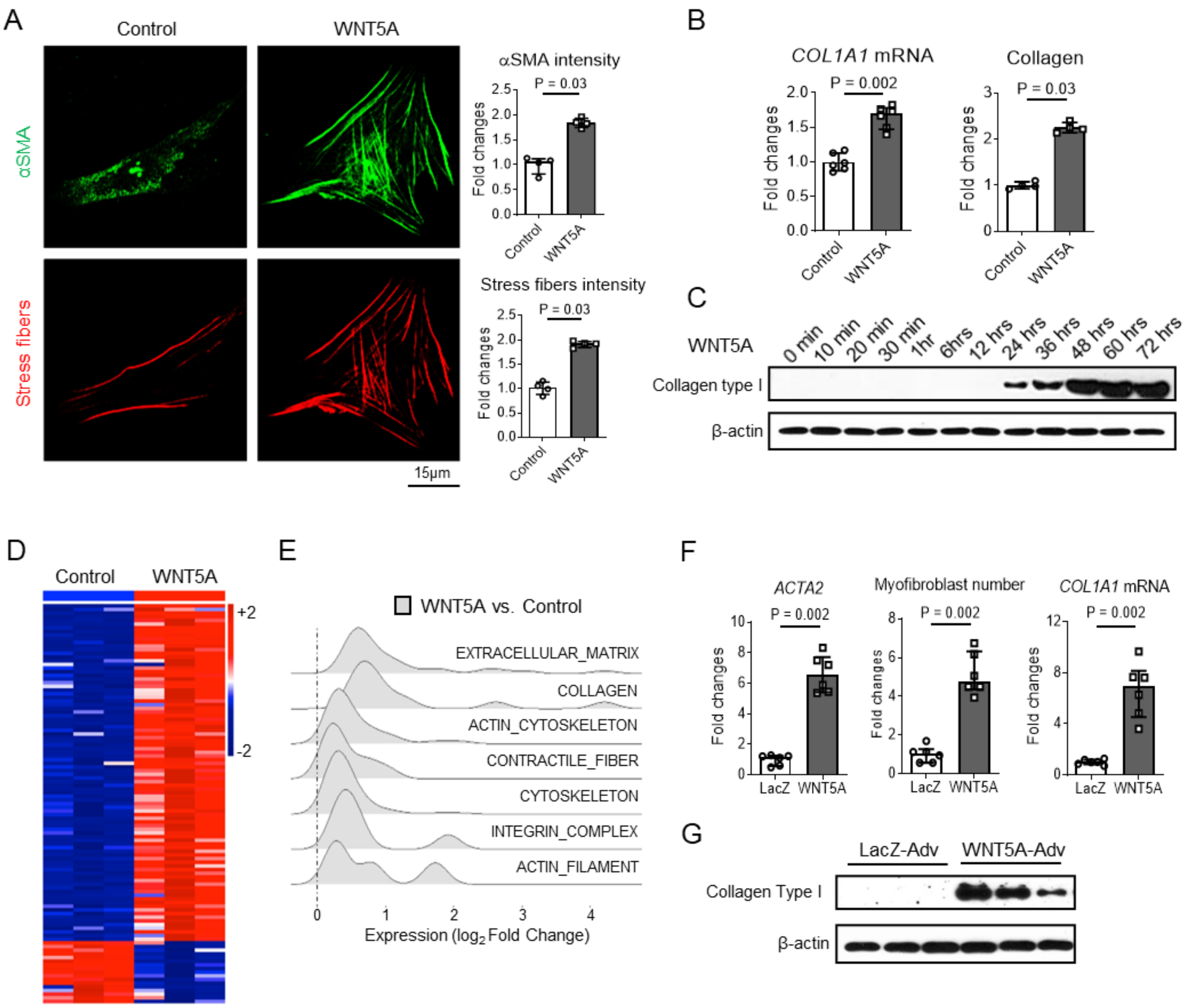

Supplementary Figure 2: WNT5A promotes fibroblast-to-myofibroblast transition.
(**A-C**) Conventional two-dimensional fibroblast cultures: (**A**) Representative IF images and quantifications of αSMA expression (green) and stress fiber formation (red) in human dermal fibroblasts stimulated with recombinant WNT5A (n = 4 each group). (**B**) mRNA levels of *COL1A1* as analyzed by real-time qPCR (n = 6 each group) and release of collagen protein as analyzed by SirCol assays (n = 4 each group). (**C**) Representative Western blot for type I collagen in WNT5A stimulated healthy fibroblasts (n = 3 each group). (**D-E**) RNAseq of human dermal fibroblasts stimulated with WNT5A compared to control fibroblasts (n = 3 each group): (**D**) Heatmap illustration of differentially expressed genes (DEGs). (**E**) Ridgeline plots highlighting the enrichment of GSEA-gene sets related to fibroblast-to-myofibroblast transition and tissue fibrosis. (**F-G**) Full-thickness skin model: (**F**) mRNA levels of *ACTA2*, myofibroblast counts and mRNA level of *COL1A1* in the presence or absence of recombinant WNT5A (n = 6 each group). (**G**) Representative Western blot for human type I collagen (n = 3 each group). Results are shown as median ± IQR with data representing individual data points. The statistical significance was determined by two-tailed Mann-Whitney *U*-test. Adv: Adenovirus.

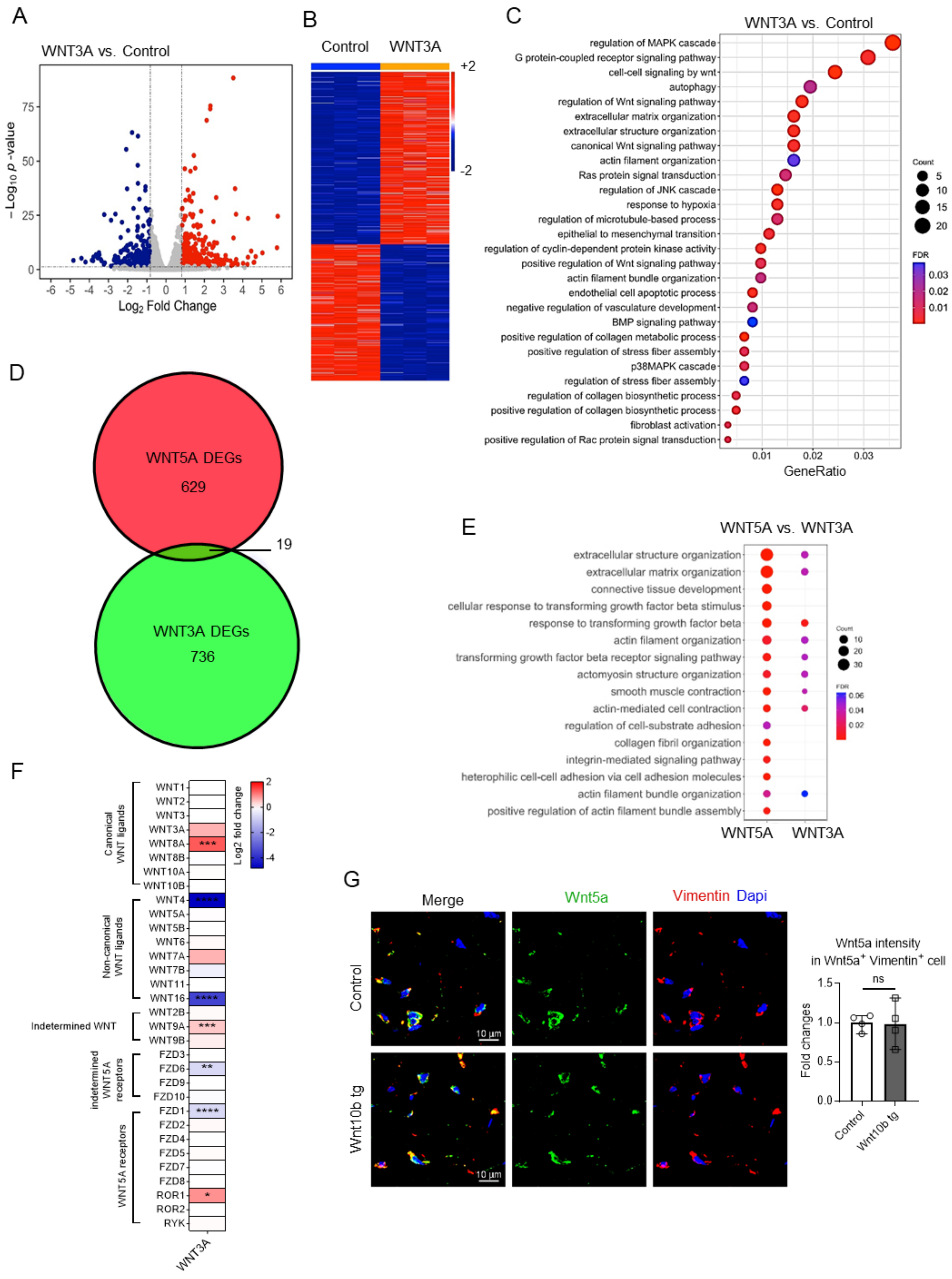

A
WNT3A vs. Control
−Log10 p-value
Log2 Fold Change
B
Control
WNT3A
+2
-2
C
WNT3A vs. Control
regulation of MAPK cascade
G protein-coupled receptor signaling pathway
cell-cell signaling by wnt
autophagy
regulation of Wnt signaling pathway
extracellular matrix organization
extracellular structure organization
canonical Wnt signaling pathway
actin filament organization
Ras protein signal transduction
regulation of JNK cascade
response to hypoxia
regulation of microtubule-based process
epithelial to mesenchymal transition
regulation of cyclin-dependent protein kinase activity
positive regulation of Wnt signaling pathway
actin filament bundle organization
endothelial cell apoptotic process
negative regulation of vasculature development
BMP signaling pathway
positive regulation of collagen metabolic process
positive regulation of stress fiber assembly
p38MAPK cascade
regulation of stress fiber assembly
regulation of collagen biosynthetic process
positive regulation of collagen biosynthetic process
fibroblast activation
positive regulation of Rac protein signal transduction
GeneRatio
Count
FDR
D
WNT5A DEGs
629
19
WNT3A DEGs
736
E
WNT5A vs. WNT3A
extracellular structure organization
extracellular matrix organization
connective tissue development
cellular response to transforming growth factor beta stimulus
response to transforming growth factor beta
actin filament organization
transforming growth factor beta receptor signaling pathway
actomyosin structure organization
smooth muscle contraction
actin-mediated cell contraction
regulation of cell-substrate adhesion
collagen fibril organization
integrin-mediated signaling pathway
heterophilic cell-cell adhesion via cell adhesion molecules
actin filament bundle organization
positive regulation of actin filament bundle assembly
WNT5A
WNT3A
F
Canonical WNT ligands
Non-canonical WNT ligands
Indetermined WNT
indetermined WNT5A receptors
WNT5A receptors
WNT1
WNT2
WNT3
WNT3A
WNT8A ***
WNT8B
WNT10A
WNT10B
WNT4 ****
WNT5A
WNT5B
WNT6
WNT7A
WNT7B
WNT11
WNT16 ****
WNT2B
WNT9A ***
WNT9B
FZD3
FZD6 **
FZD9
FZD10
FZD1 ****
FZD2
FZD4
FZD5
FZD7
FZD8
ROR1 *
ROR2
RYK
WNT3A
Log2 fold change
G
Merge
Wnt5a
Vimentin Dapi
Control
Wnt10b tg
10 µm
Wnt5a intensity
in Wnt5a+ Vimentin+ cell
ns
Fold changes
Control
Wnt10b tg

Supplementary Figure 3: Canonical and non-canonical WNT signaling induce different gene sets in human dermal fibroblasts.

(**A**) Volcano plot of Differentially Expressed Genes (DEGs) of RNA sequencing data from human dermal fibroblasts stimulated with WNT3A compared to stimulation with the solvent DPBS (WNT3A DEGs). (**B**) Heatmap illustration of WNT3A DEGs. (**C**) De-enrichment of Gene Ontology (GO) biological processes related to fibroblast-to-myofibroblast transition and fibrosis (n = 3 each group). (**D**) Overlap of WNT3A DEGs and WNT5A DEGs. (**E**) Comparison of negative enrichment scores for GO biological processes related to fibroblast-to-myofibroblast transition and fibrosis between WNT5A- and WNT3A -RNA sequencing data. (**F**) Screening of fibroblasts stimulated with WNT3A for changes in the mRNA level of WNT ligands and WNT receptors. * indicates differences of WNT3A-stimulated fibroblasts compared to DPBS stimulated fibroblasts. *: FDR < 0.05; **: FDR < 0.01; ***: FDR < 0.001; and ****: FDR < 0.0001. (**G**) Representative confocal images of Wnt5a expression in the skin of Wnt10b transgenic mice and control littermates (n = 4 each group). Results are shown as median ± IQR with data representing individual data points. The statistical significance was determined by two-tailed Mann-Whitney U-test. FDR means False-Discovery-Rate.

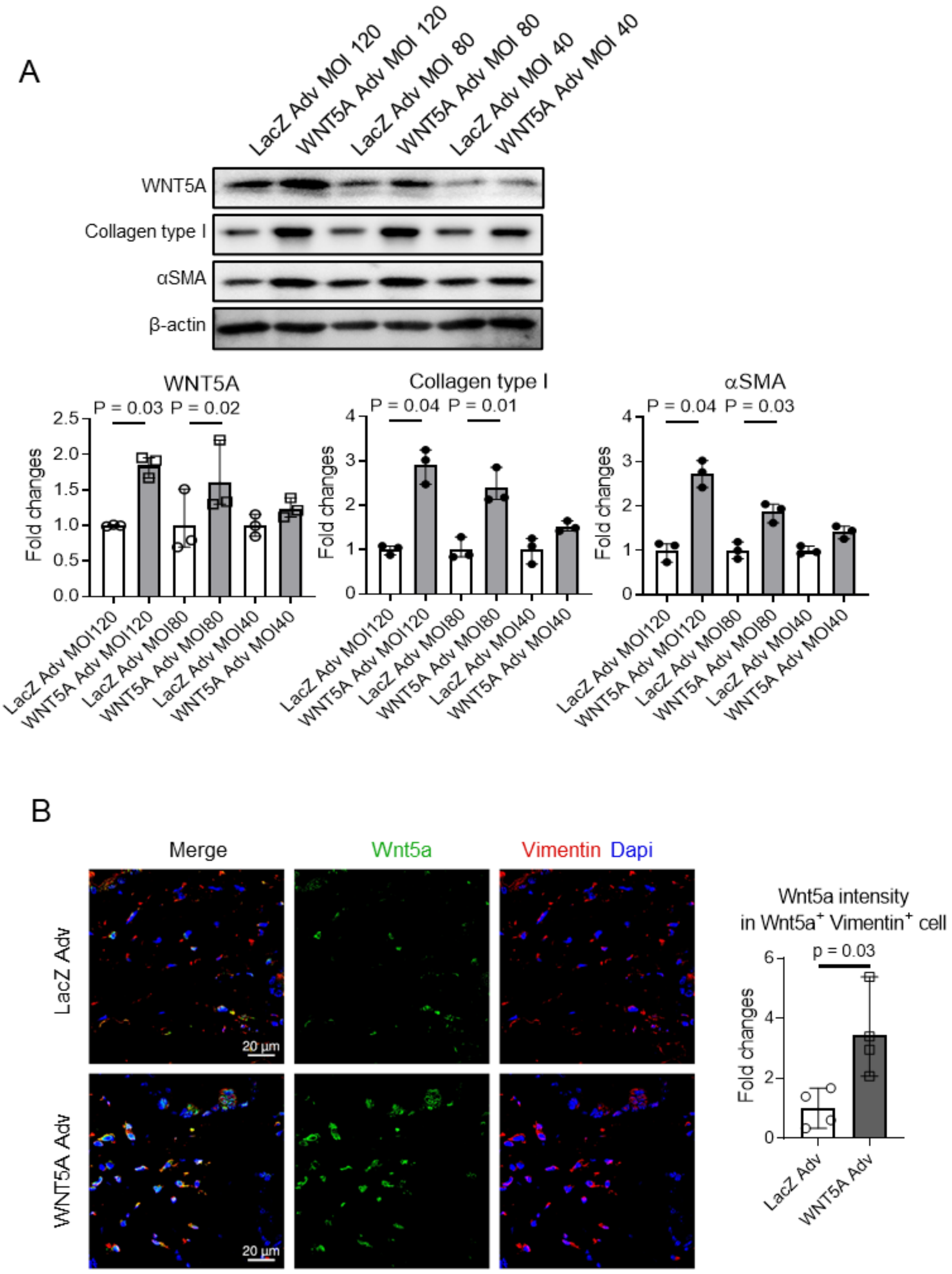

Supplementary Figure 4: Adenoviral overexpression of WNT5A enhances collagen production and alpha smooth muscle actin expression similar to recombinant WNT5A. (**A**) Representative images of Western blots (Collagen type I, WNT5A, αSMA and β-actin) of lysates from fibroblasts infected with LacZ or WNT5A adenoviruses at different multiplicity of infection (MOI) (n = 3 each group). (**B**) Representative immunofluorescence staining images and quantification of the increase in the levels of Wnt5a upon adenoviral overexpression in murine skin (n = 4 each group). Results are shown as mean ± IQR with data representing individual data points. The statistical significance was determined by two-tailed Mann-Whitney *U*-test, if two groups were compared, or one-way ANOVA with Sidak's multiple comparison test in case of more than two comparisons. Adv: Adenovirus.

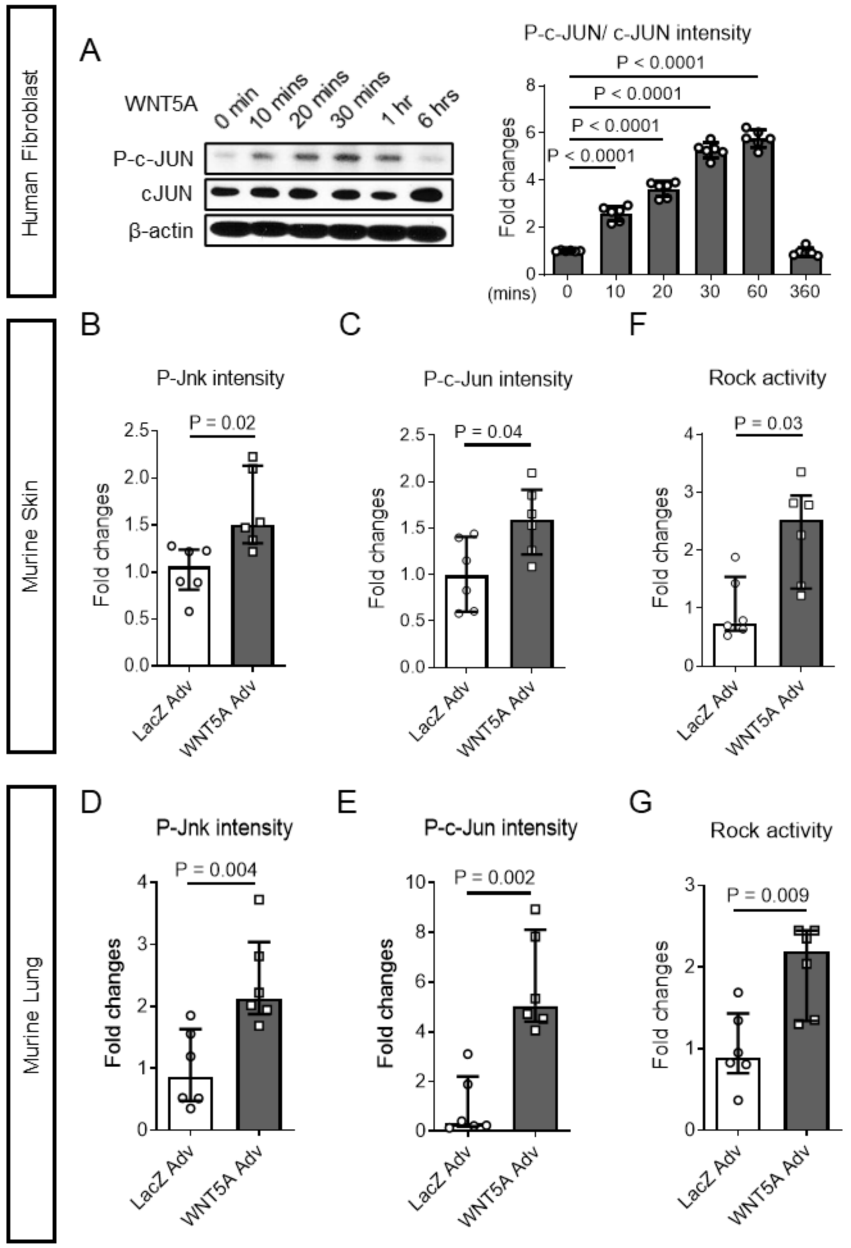

Supplementary Figure 5: Adenoviral overexpression of WNT5A promotes accumulation of P-Jnk, P-c-Jun and Rock activity in murine skin and lung.

(**A-E**) JNK signaling: (**A**) Representative Western blot and quantification of P-c-JUN protein in WNT5A stimulated human dermal fibroblasts (n = 6 each group). (**B-E**) Quantifications of P-Jnk and P-c-Jun in (**B-C**) murine skin and (**D-E**) lung (n = 6 each group). (**F-G**) Rock signaling: Rock activity measured in (**F**) murine skin and (**G**) lungs (n = 6 each group). Results are shown as median ± IQR with data representing individual data points. The statistical significance was determined by two-tailed Mann-Whitney *U*-test, if two groups were compared, or one-way ANOVA with Tukey's multiple comparison test in case of more than two comparisons. Adv: Adenovirus.

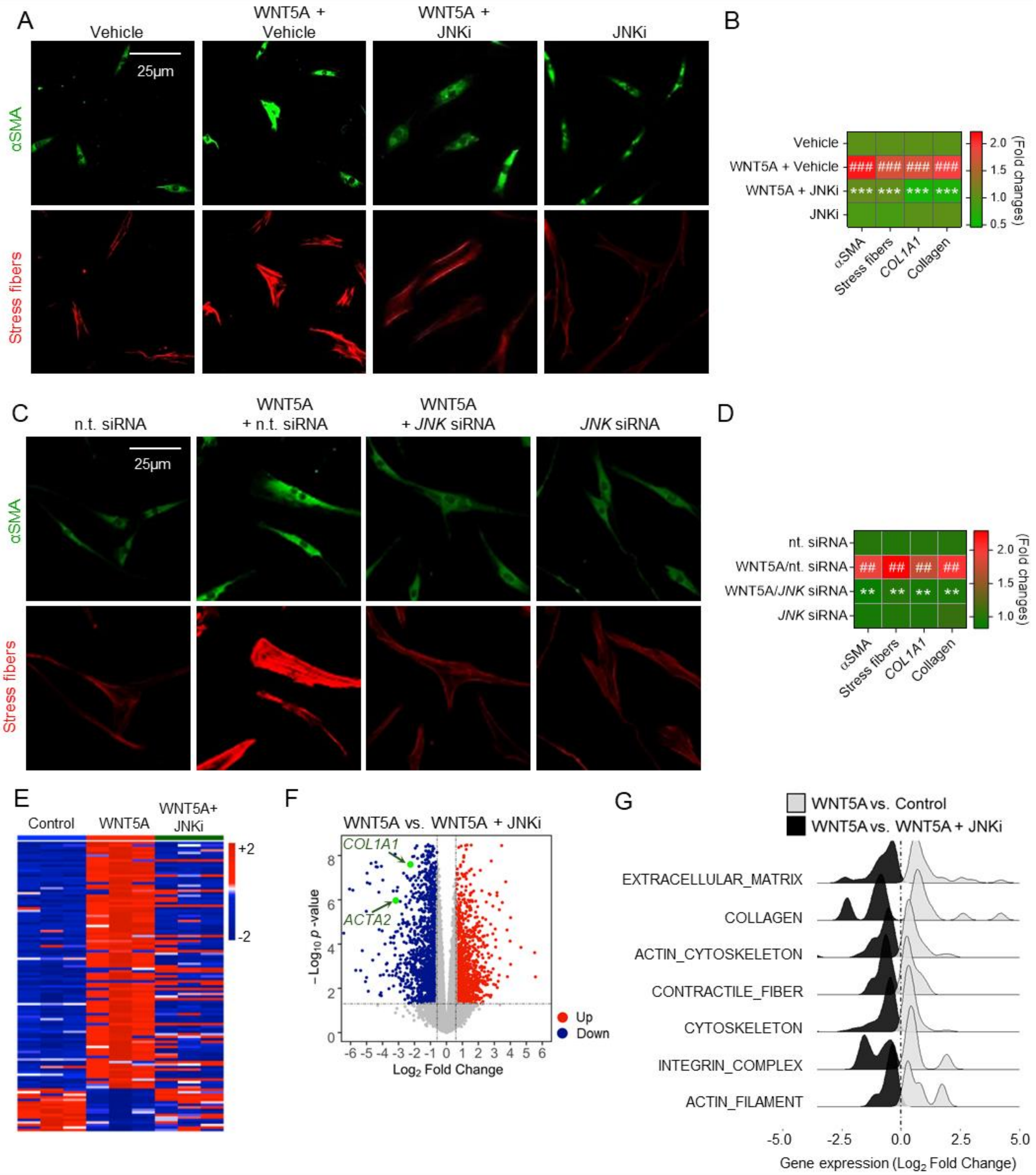

Supplementary Figure 6: WNT5A-induced fibroblast activation is prevented by inactivation of JNK.

(**A**) Representative IF images of αSMA and stress fibers in WNT5A-stimulated fibroblasts incubated with or without SP600125 (JNK inhibitor, JNKi). (**B**) Changes in WNT5A-induced collagen release, αSMA expression and stress fiber formation upon JNK inhibition shown as heatmap (n = 3 each group). (**C**) Representative IF images of αSMA and stress fibers in WNT5A-stimulated fibroblasts transfected with *JNK* siRNA or non-targeting siRNA (n.t. siRNA). (**D**) Changes in WNT5A-induced collagen release, αSMA expression and stress fiber formation upon JNK-knockdown shown as heatmap (n = 3 each group). (**E**) Heatmap

illustration and (**F**) Volcano plot of DEGs in WNT5A-stimulated human dermal fibroblasts with or without JNK inhibitor. (**G**) Ridgeline plot highlighting the de-enrichment of GSEA-gene sets related to fibroblast-to-myofibroblast transition and tissue fibrosis in fibroblasts incubated with WNT5A plus JNKi as compared to WNT5A-stimulated fibroblasts. The statistical significance was determined one-way ANOVA with Tukey's multiple comparison test. # indicates significant differences of WNT5A stimulated fibroblasts compared to controls. * indicates significant differences of WNT5A-stimulated fibroblasts treated with JNK inhibitor compared to WNT5A stimulated fibroblasts incubated with vehicle. # and *: $P < 0.05$; ## and **: $P < 0.01$; ### and ***: $P < 0.001$.

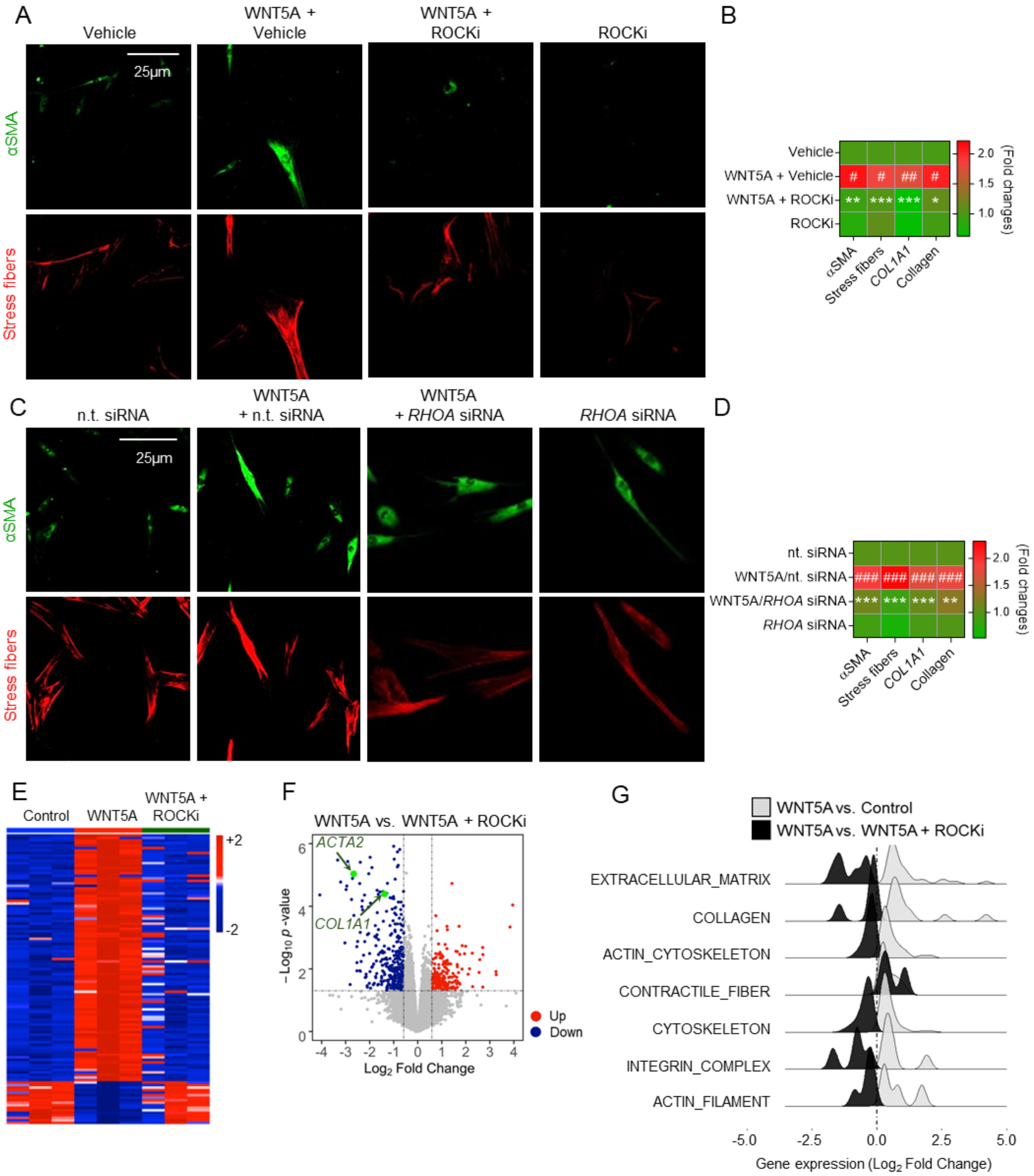

Supplementary Figure 7: WNT5A-induced fibroblast activation is prevented by inactivation of ROCK.

(**A**) Representative IF images of αSMA and stress fibers in WNT5A-stimulated fibroblasts incubated with or without Y27632 (ROCK inhibitor, ROCKi). (**B**) Changes in WNT5A-induced collagen release, αSMA expression and stress fiber formation upon ROCK inhibition shown as heatmap (n = 3 each group). (**C**) Representative IF images of αSMA and stress fibers in WNT5A-stimulated fibroblasts transfected with *RHOA* siRNA or non-targeting siRNA (n.t. siRNA). (**D**) Changes in WNT5A-induced collagen release, αSMA expression and stress fiber

formation by *RHOA*-knockdown shown as heatmap (n = 3 each group). (**E**) Heatmap illustration and (**F**) Volcano plot of DEGs in WNT5A-stimulated human dermal fibroblasts with or without ROCKi. (**G**) Ridgeline plot highlighting the de-enrichment (negative enrichment scores) of WNT5A-induced GSEA-gene sets related to fibroblast-to-myofibroblast transition and tissue fibrosis by ROCKi. The statistical significance was determined one-way ANOVA with Tukey's multiple comparison test. # indicates differences of WNT5A stimulated fibroblasts compared to controls. * indicates differences of WNT5A-stimulated fibroblasts treated with ROCK inhibitor compared to WNT5A stimulated fibroblasts incubated with vehicle. # and *: P < 0.05; ## and **: P < 0.01; ### and ***: P < 0.001.

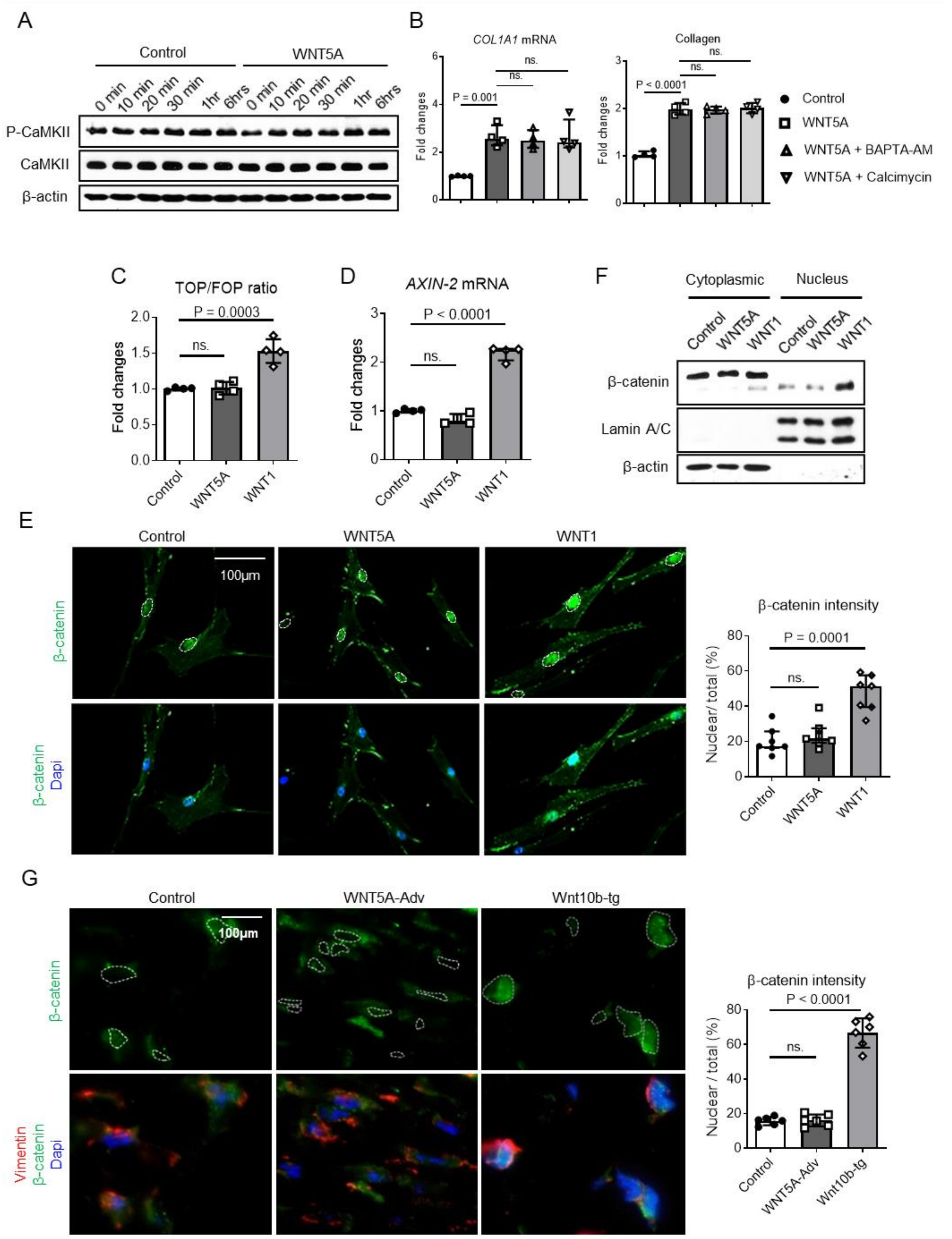

Supplementary Figure 8: WNT5A does not activate fibroblasts via WNT/β-catenin signaling or WNT/calcium signaling.

(**A-B**) WNT/calcium signaling: (**A**) Representative Western Blots of phosphorylated and total CAMK2 in fibroblasts stimulated with WNT5A for different time points (n = 3 each group). (**B**) Levels of *COL1A1* mRNA and soluble collagen protein in WNT5A-stimulated fibroblasts upon incubation with BAPTA-AM or Calcimycin (n = 4 each group).

(**C-G**) Canonical WNT signaling: (**C**) TOP/FOP luciferase assay showing changes of TCF-dependent transcription after stimulation of fibroblasts with WNT1 or WNT5A (n = 4 each group). (**D**) mRNA levels of the β-catenin/TCF target gene *AXIN-2* (n = 4 each group). (**E**) Representative confocal images of the expression levels and the subcellular localization of β-catenin in fibroblasts stimulated with WNT1 or WNT5A (left, dotted lines indicate the nucleus) with quantification of the intensity of nuclear β-catenin (right) (n = 7 each group). (**F**) Representative Western blots showing the levels of β-catenin in nuclear and cytoplasmic subcellular fractions with Lamin A/C and β-actin loading controls (n = 3 each group). (**G**) Representative IF images and quantification of the nuclear/total levels of β-catenin in mice overexpressing WNT5A and in Wnt10b transgenic mice (n = 6 each group). Results are shown as median ± IQR with data representing individual data points. The statistical significance was determined one-way ANOVA with Tukey's multiple comparison test. ns. indicates no significant differences. tg: transgenic mice. Adv: Adenovirus.

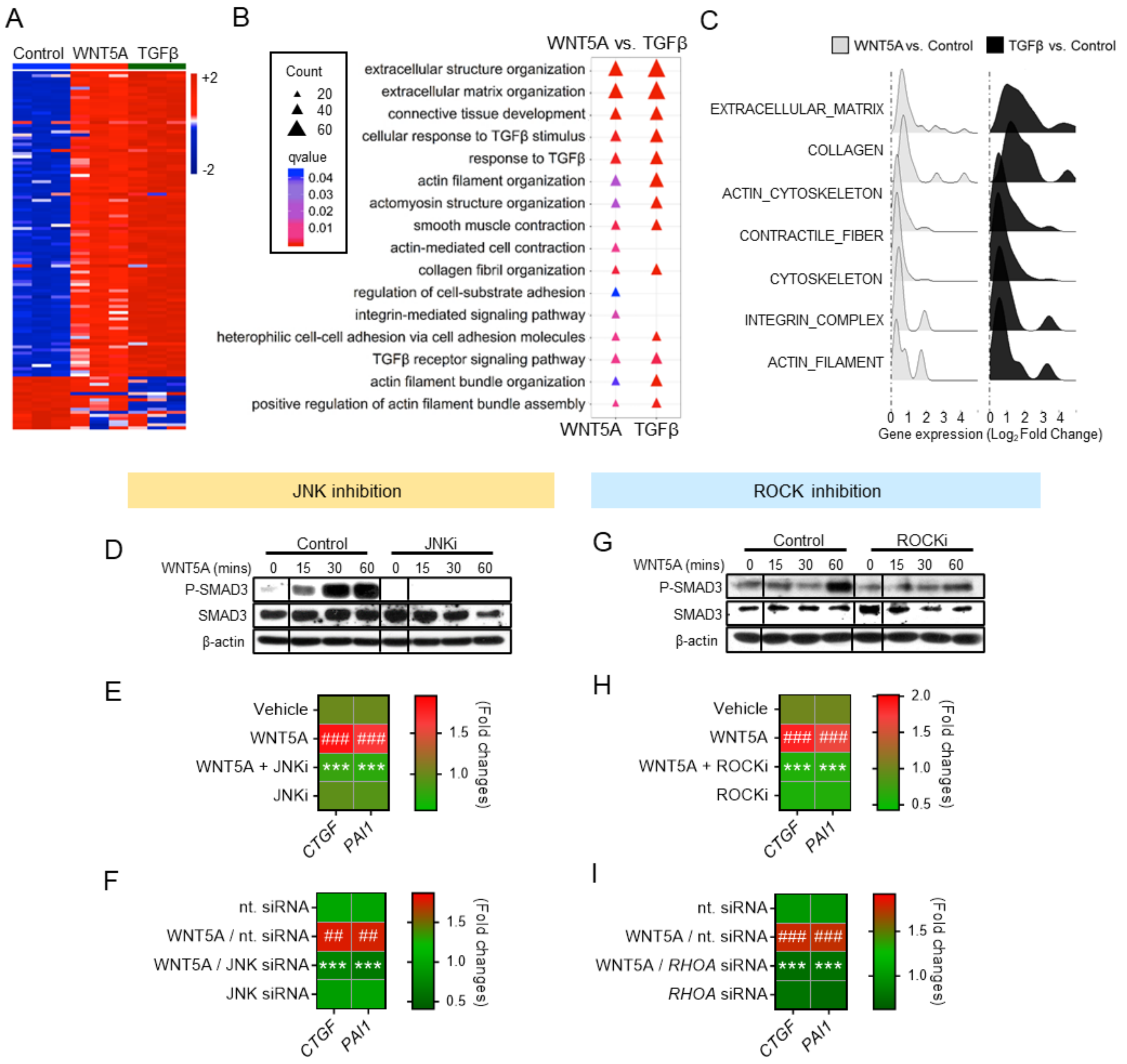

Supplementary Figure 9: Selective Inhibition of JNK or ROCK abolishes WNT5A induced TGFβ/SMAD3 signaling.

(**A-C**) Comparison of WNT5A- and TGFβ-induced transcriptomes in human dermal fibroblasts: (**A**) Heatmap illustration comparing the DEGs in WNT5A-stimulated fibroblasts and TGFβ-stimulated fibroblasts. (**B**) Enrichment of GO biological processes related to fibroblast-to-myofibroblast transition and fibrosis in WNT5A-stimulated and TGFβ-stimulated human dermal fibroblasts. (**C)** Ridgeline plot showing similar enrichment of GSEA-gene sets related to fibroblast-to-myofibroblast transition and tissue fibrosis in fibroblasts incubated with WNT5A and in TGFβ-stimulated fibroblasts (n = 3 each group). (**D-F**) Effects of JNK inhibition on WNT5A induced TGFβ/SMAD3 signaling: (**D**) Representative Western blot for P-SMAD3 and total SMAD3 (n = 3 each group). The black thin vertical lines were drawn to separate non-contiguous lanes, which are on the same gel. Changes in the levels of the prototypical TGFβ/SMAD3 target genes *CTGF* and *PAI-1* in fibroblasts incubated with (**E**) JNKi or with (**F**) siRNA-mediated knockdown of JNK (n = 3 each group). (**G-I**) Effects of ROCK inhibition on WNT5A induced TGFβ/SMAD3 signaling: (**G**) Representative Western blot for P-SMAD3 and total SMAD3 (n = 3 each group). The black thin vertical lines were drawn to separate noncontiguous lanes, which are on the same gel. Changes in the levels of the

prototypical TGFβ/SMAD3 target genes *CTGF* and *PAI-1* in fibroblasts incubated with (**H**) ROCKi or with (**I**) siRNA-mediated knockdown of RHOA (n = 3 each group). The statistical significance was determined one-way ANOVA with Tukey's multiple comparison test. # indicates differences of WNT5A stimulated fibroblasts compared to controls. * indicates differences of WNT5A-stimulated fibroblasts treated with inhibitor compared to WNT5A stimulated fibroblasts incubated with vehicle. # and *: $P < 0.05$; ## and **: $P < 0.01$; ### and ***: $P < 0.001$. JNKi: JNK inhibitor. ROCKi: ROCK inhibitor.

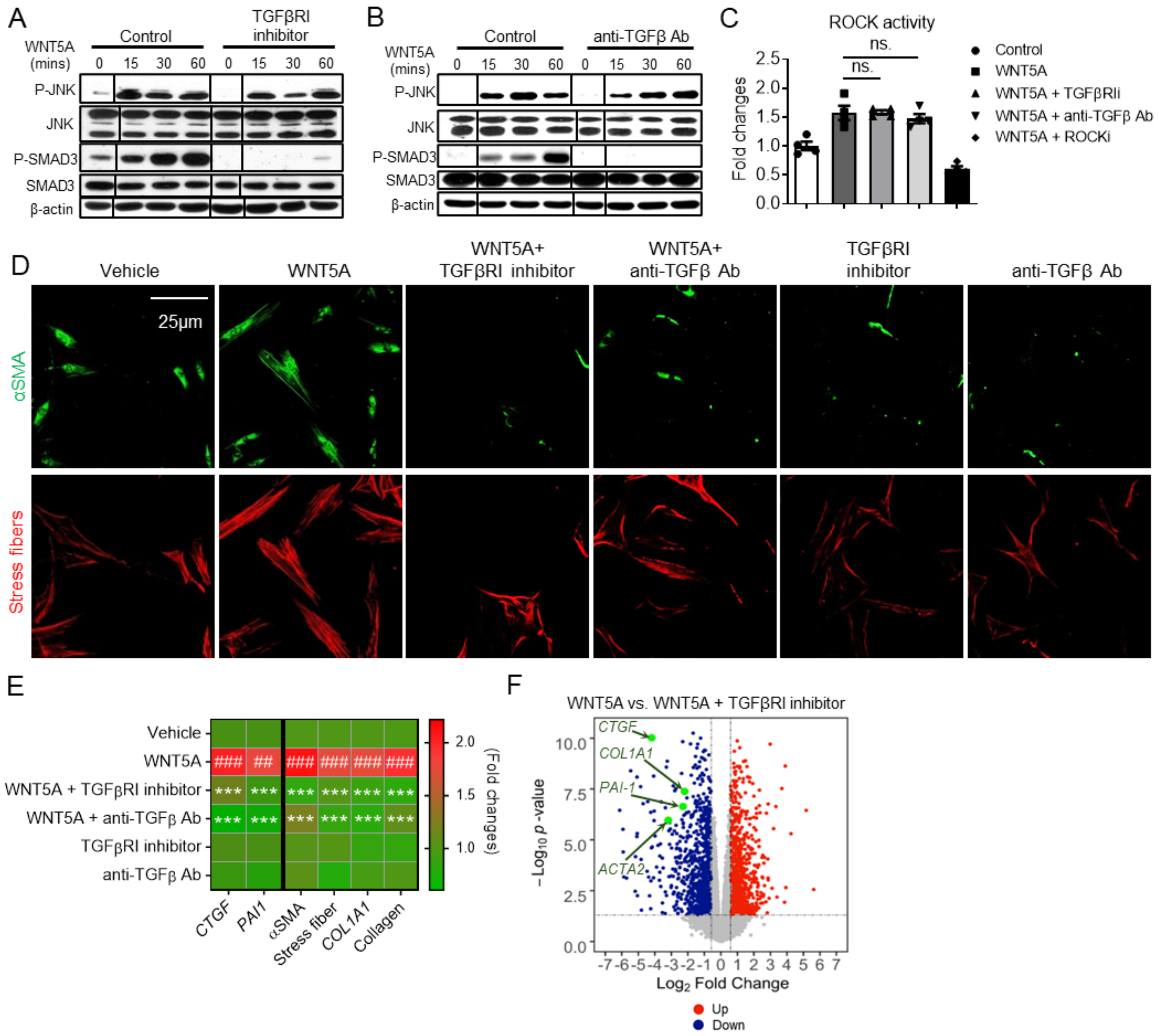

Supplementary Figure 10: inhibition of TGFβ signaling blocks WNT5A-induced fibroblast-to-myofibroblast transition.

(**A**) Representative Western blot for P-JNK, total JNK, P-SMAD3 and total SMAD3 in fibroblasts incubated with the TGFβ receptor I inhibitor SD208 (TGFβRIi) or (**B**) with neutralizing anti-TGFβ antibodies (anti-TGFβ Ab) (n = 3 each group). The lanes were run on the same gel, but were non-contiguous. The black thin vertical lines were drawn to separate non-contiguous lanes. (**C**) ROCK activity in WNT5A-stimulated fibroblasts incubated with TGFβRIi, anti-TGFβ Ab or ROCKi (n = 4 each group). (**D**) Representative IF images of αSMA expression and stress fiber formation in WNT5A-stimulated fibroblasts with and without TGFβR1i or TGFβ Ab (n = 3 each group). (**E**) Changes in the TGFβ downstream target genes *CTGF* and *PAI-1*, and in the levels of αSMA, of stress fibers, of *COL1A1* mRNA and of soluble collagen protein as fibrotic readouts in WNT5A-stimulated fibroblasts upon incubation with TGFβRIi or TGFβ Ab shown as heatmaps (n = 3 each group). (**F**) Volcano plot of DEGs in WNT5A-stimulated human dermal fibroblasts with or without TGFβRIi (n = 3 each group). The statistical significance was determined one-way ANOVA with Tukey's multiple comparison test. # indicates differences of WNT5A stimulated fibroblasts compared to controls. * indicates differences of WNT5A-stimulated fibroblasts treated with inhibitor compared to

WNT5A stimulated fibroblasts incubated with vehicle. # and *: P < 0.05; ## and **: P < 0.01; ### and ***: P < 0.001; ns. indicates no significant differences.

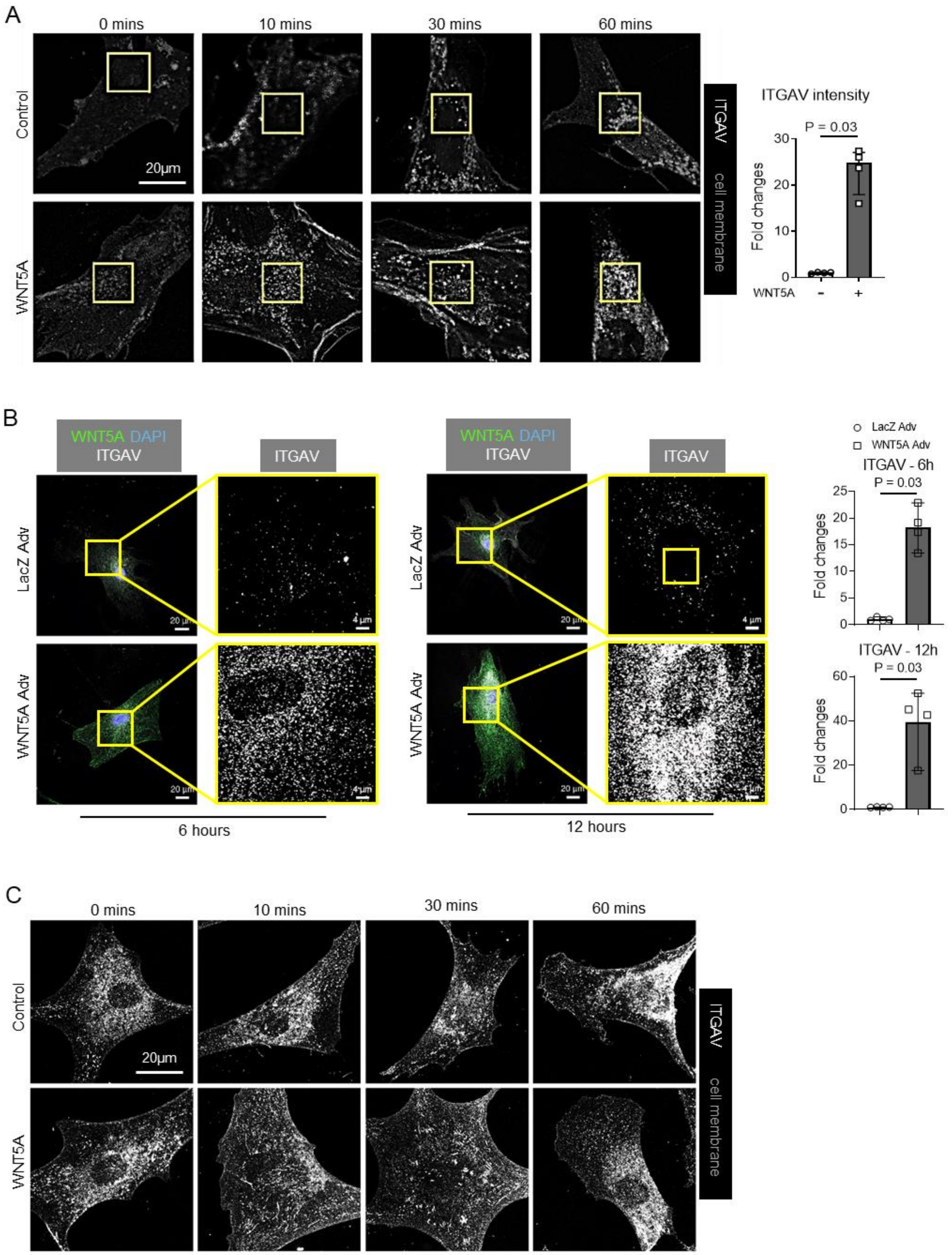

Supplementary Figure 11: WNT5A induces integrin alpha V, not integrin alpha 5, clustering in fibroblasts.

(**A**) Representative confocal IF images (lower magnification) and quantification of WNT5A-induced clustering of ITGAV in cultured dermal fibroblasts (n = 4 each group). (**B**) Representative confocal images and quantifications of ITGAV clustering and WNT5A expression in fibroblasts infected with LacZ or WNT5A adenoviruses for 6 hours or 12 hours (n = 4 each group). (**C**) Representative confocal IF images of ITGA5 clustering in cultured dermal fibroblasts exposed to WNT5A (n = 3 each group). Results are shown as median ± IQR with data representing individual data points. The statistical significance was determined by two-tailed Mann-Whitney *U*-test. Adv: Adenovirus.

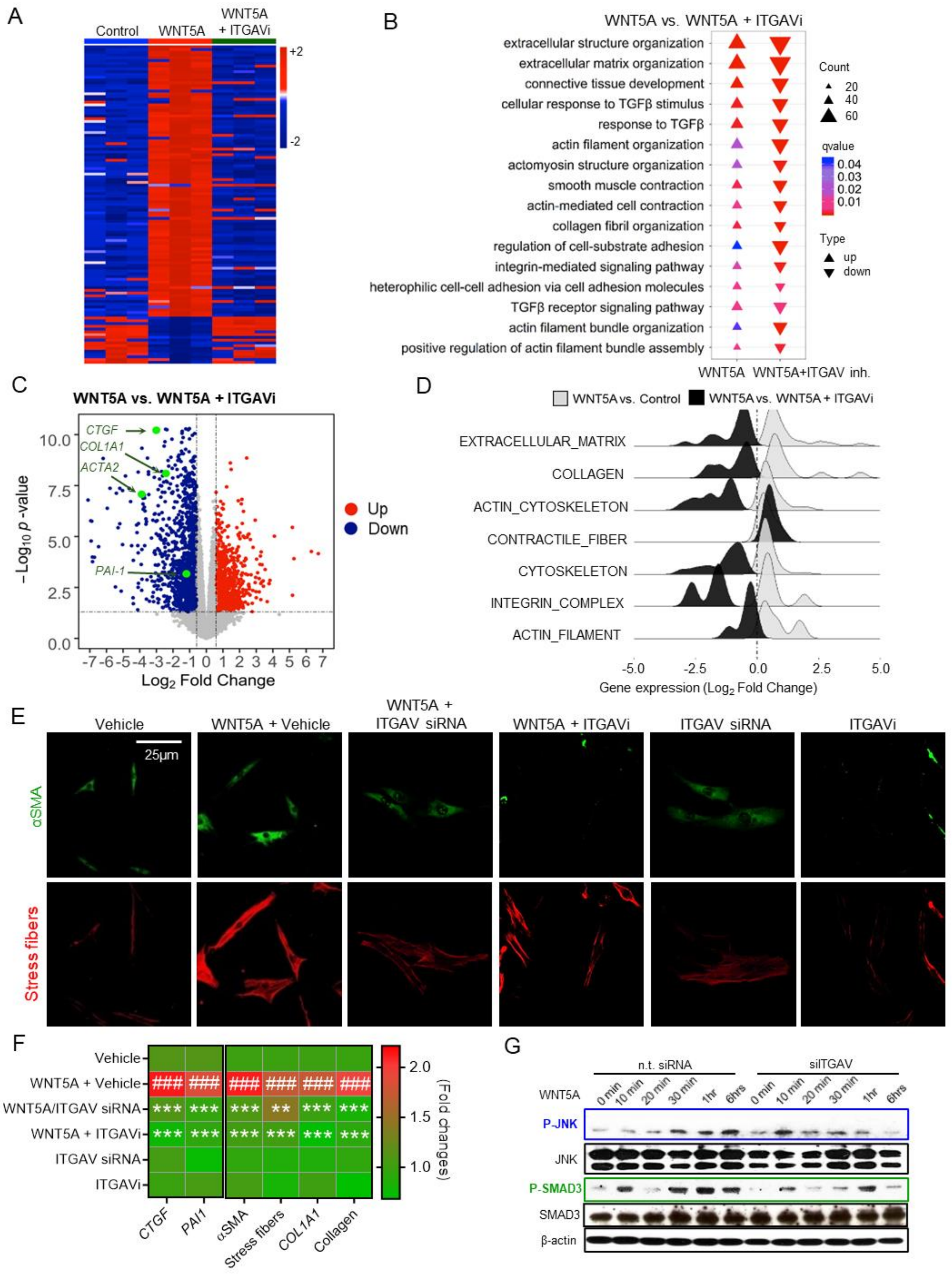

Supplementary Figure 12: The profibrotic effects of WNT5A are blocked by inhibition of ITGAV.

(**A**) Heatmap illustration of DEGs in WNT5A-stimulated fibroblasts with ITGAV inhibitor (ITGAVi) compared to WNT5A stimulated fibroblasts (n = 3 each group). (**B**) Negative enrichment scores (de-enrichment) of GO biological processes related to fibroblast-to-myofibroblast transition and fibrosis. (**C**) Volcano plot of DEGs in WNT5A stimulated human dermal fibroblasts treated with ITGAVi or with vehicle. (**D**) Ridgeline plot highlighting the de-enrichment of WNT5A-induced GSEA-gene sets related to fibroblast-to-myofibroblast transition and tissue fibrosis in fibroblasts incubated with WNT5A with or without ITGAVi (n = 3 each group). (**E**) Representative IF images of αSMA and stress fibers in WNT5A-stimulated fibroblasts with and without *ITGAV* siRNA or ITGAVi compared to respective controls. (**F**) Changes of the WNT5A-induced upregulation of *CTGF* and *PAI-1* mRNA, in αSMA expression and stress fiber formation, of *COL1A1* mRNA and in the levels of soluble collagen by *ITGAV* siRNA or ITGAVi compared to respective controls (n = 3 each group) as shown by heatmaps. (**G**) Representative Western blot images of pJNK and pSMAD3 in fibroblasts stimulated with WNT5A upon transfection with ITGAV siRNA or non-targeting siRNA at different time points (n = 3). statistical significance were determined by one-way ANOVA with Tukey's multiple comparison test. # indicates differences of WNT5A stimulated fibroblasts compared to controls. * indicates differences of WNT5A-stimulated fibroblasts treated with inhibitor or target siRNA compared to WNT5A -stimulated fibroblasts incubated with vehicle. ## and **: $P < 0.01$; ### and ***: $P < 0.001$. ITGAVi: Integrin αV inhibitor.

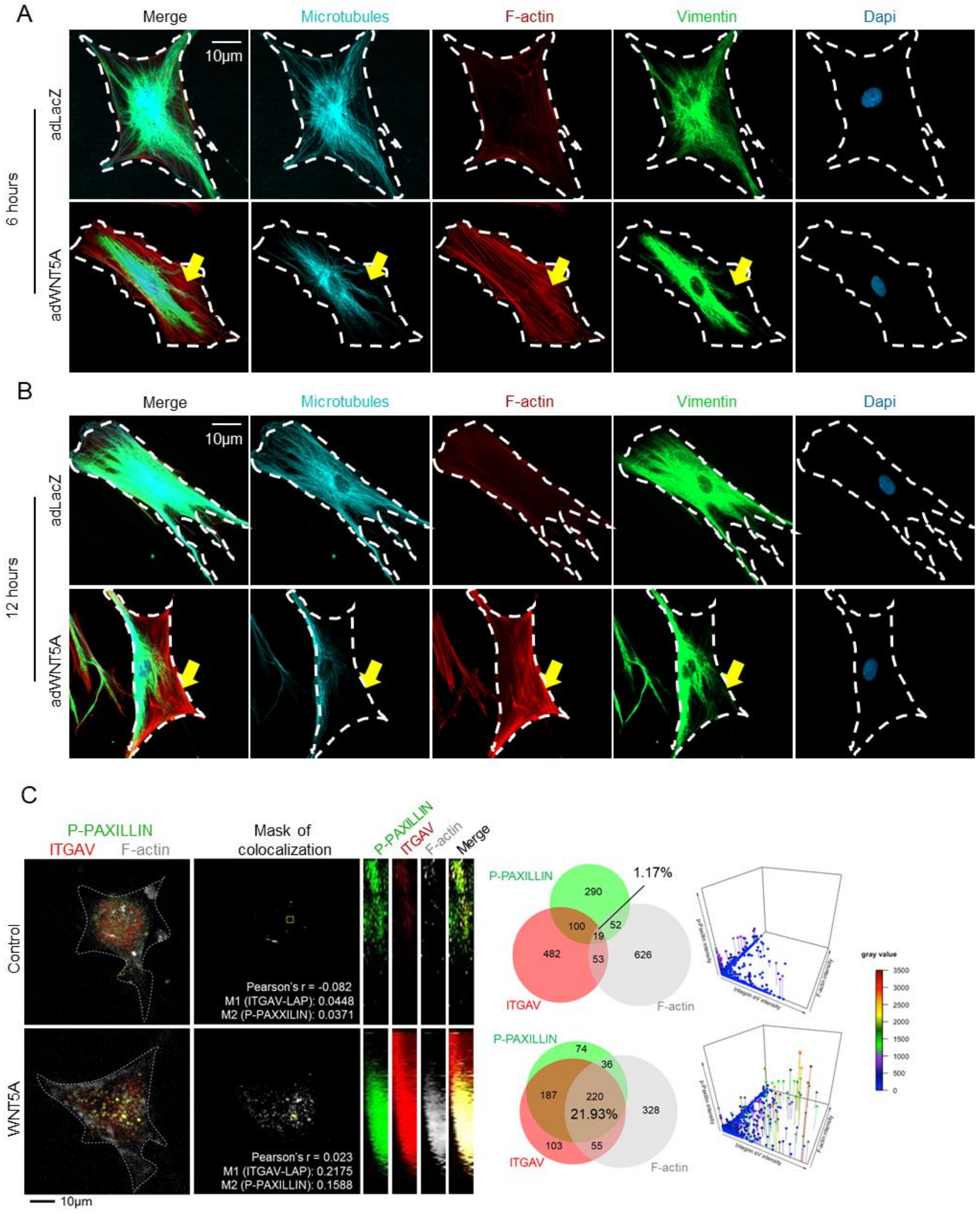

Supplementary Figure 13: Adenoviral overexpression of WNT5A induces cytoskeleton reorganization in fibroblasts.

(**A**) Representative images of cytoskeleton reorganization in fibroblasts upon infection with adenoviruses encoding for LacZ or WNT5A for 6 hours (n = 5 each group). (**B**) Representative images of cytoskeleton reorganization after 12 hours (n = 5 each group). Microtubules, F-actin,

Vimentin and the nucleus are stained as turquoise, red, green and blue, respectively. The yellow thick arrows point to the area of cytoskeleton rearrangement, in which F-actin is bundled and polymerized, while microtubules and vimentin filaments are dissociating and depolymerizing. The white dashed lines indicate cell borders. (**C**) Representative confocal images and Z-stack reconstructions showing colocalization of F-actin with ITGAV and P-PAXILLIN along with Venn diagrams demonstrating the relative colocalization of F-Actin with ITGAV and P-PAXILLIN and three-dimensional scatter plots showing the distribution and the intensity of each marker at each localization ($n \geq 10$ each group).

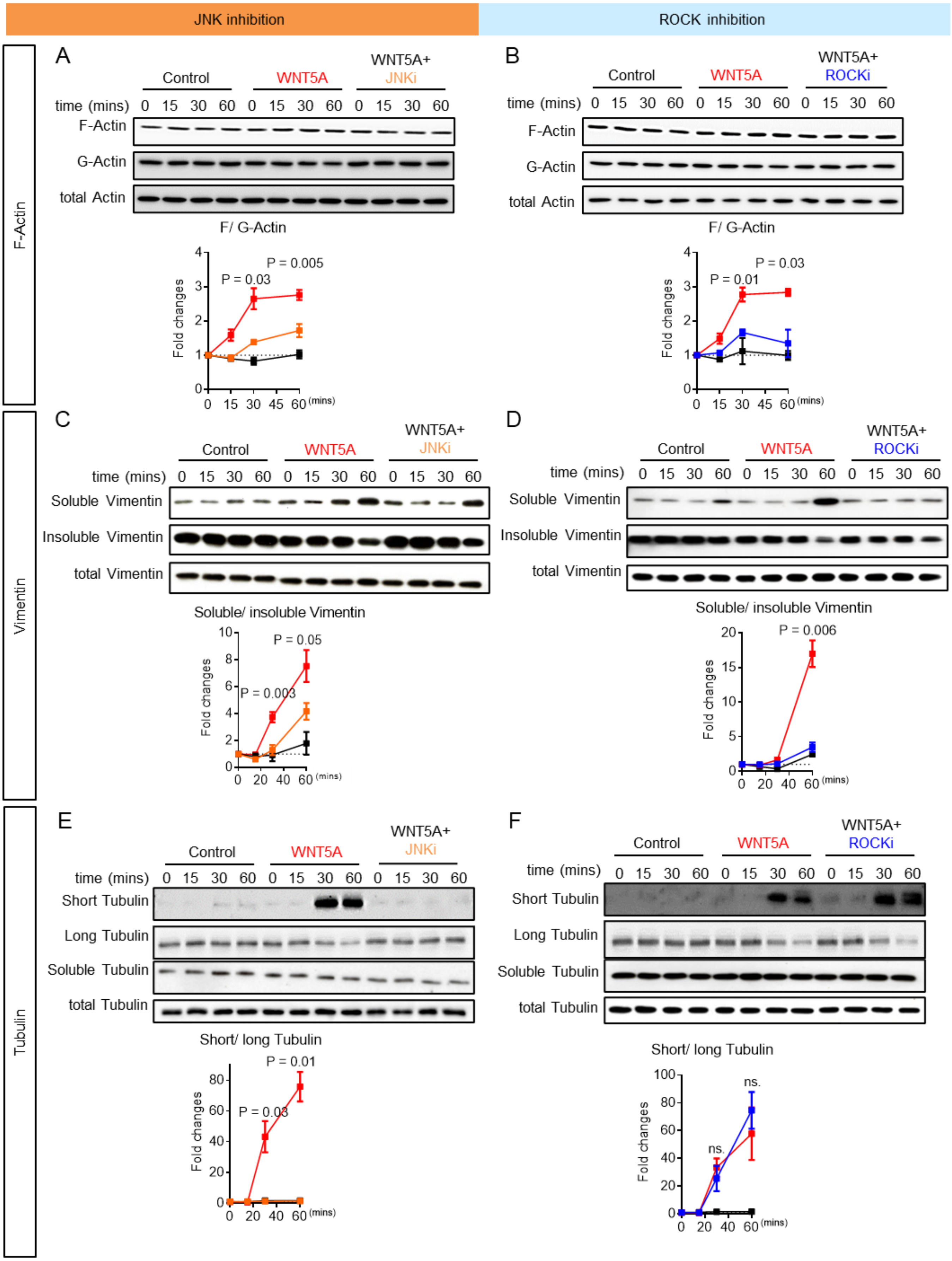

Supplementary Figure 14: JNK and ROCK coordinate WNT5A-induced changes in the organization of F-actin, Vimentin and Tubulin.

Cytoskeletal reorganization: WNT5A-induced shifts in the ratio of F-Actin to G-Actin (**A-B**), in the ratio of soluble to insoluble Vimentin (**C-D**) and short to long tubulin filaments (**E-F**) in the presence or absence of JNKi (**A, C, E**) or ROCKi (**B, D, F**) (n = 3 each group).
Results are shown as median ± IQR with data representing individual data points. The statistical significance was determined by one-way ANOVA with Tukey's multiple comparison test. JNKi: JNK inhibitor. ROCKi: ROCK inhibitor. ns. indicates no significant differences.

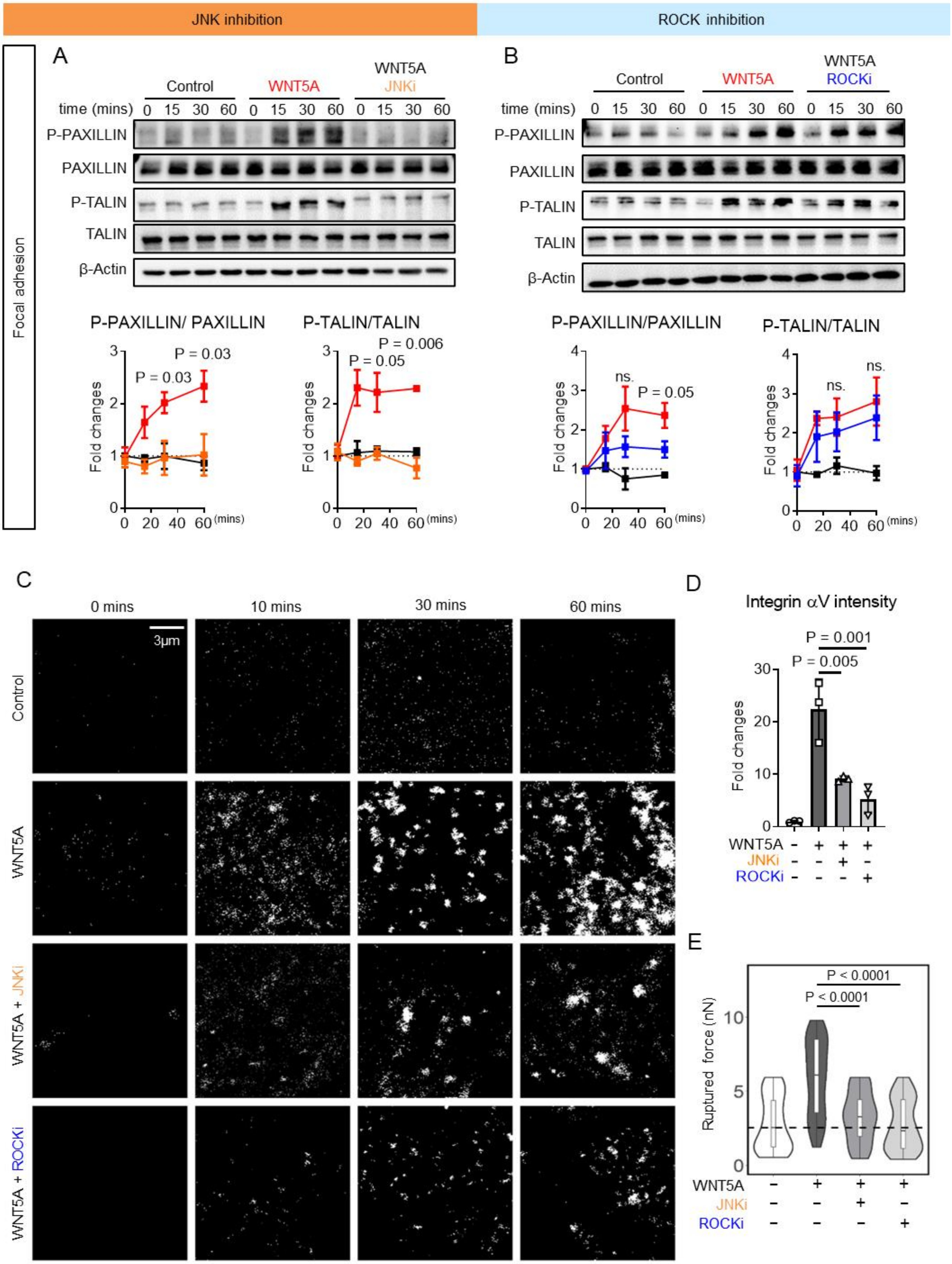

Supplementary Figure 15: JNK and ROCK coordinate WNT5A-induced changes in the formation of focal adhesions and ITGAV clustering.

(**A-B**) Formation of focal adhesions: Representative Western blots and quantifications showing the levels of P-PAXILLIN, PAXILLIN, P-TALIN and TALIN in fibroblasts stimulated with WNT5A with JNKi (**A**) or ROCKi (**B**), respectively (n = 3 each group).
(**C-E**) ITGAV clustering: (**C**) Representative confocal IF images and (**D**) quantification of WNT5A-induced clustering of ITGAV in the presence or absence of the JNKi or ROCKi (n = 3 each group). (lower magnification at the right indicating the selected area of the cell). (**E**) Violin plots showing the rupture force for LAP-TGFβ1 peptides in dermal fibroblasts incubated with WNT5A with or without JNKi or ROCKi ($n \geq 50$ each group).
Results are shown as median ± IQR with data representing individual data points. The statistical significance was determined by one-way ANOVA with Tukey's multiple comparison test. JNKi: JNK inhibitor. ROCKi: ROCK inhibitor. ns. indicates no significant differences.

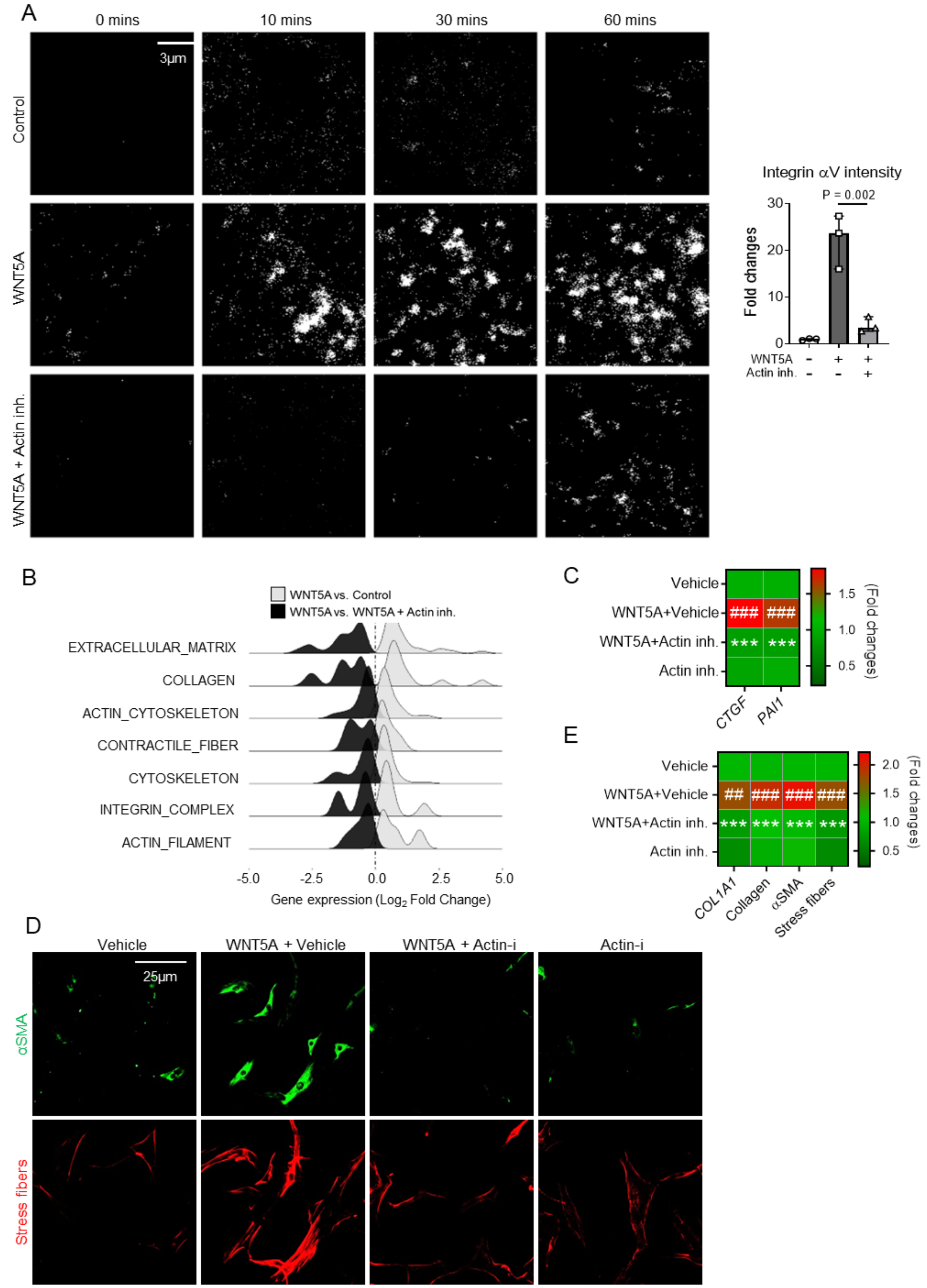

Supplementary Figure 16: WNT5A-induced fibroblast activation and tissue fibrosis requires actin rearrangement.

(**A**) Representative confocal IF images (higher magnification, above) and quantification of WNT5A-induced clustering of ITGAV in the presence or absence of the actin inhibitor Cytochalasin D (n = 3 each group, lower magnification below indicating the selected area for higher magnification). (**B**) Ridgeline plot highlighting the negative enrichment scores (de-enrichment) of WNT5A-induced GSEA-gene sets related to fibroblast-to-myofibroblast transition and tissue fibrosis by Cytochalasin D (n = 3 each group). (**C**) mRNA levels of the TGFβ/SMAD target genes *COL1A1*-, *CTGF*- and *PAI1* mRNA in fibroblasts (n = 3 each group). (**D**) Representative IF images of αSMA expression (green) and stress fiber formation (red) in fibroblasts stimulated with recombinant WNT5A with and without Cytochalasin D (n = 3 each group). (**E**) Changes in *COL1A1* mRNA, collagen release, αSMA expression and stress fiber formation shown as heatmaps (n = 3 each group). Results are shown as median ± IQR with data representing individual data points. The statistical significance was determined by one-way ANOVA with Tukey's multiple comparison test. # indicates differences of WNT5A stimulated fibroblasts compared to controls. * indicates differences of WNT5A-stimulated fibroblasts treated with inhibitor or target siRNA compared to WNT5A stimulated fibroblasts incubated with vehicle. ##: P < 0.01; ### and ***: P < 0.001. Actin-i: Actin inhibitor.

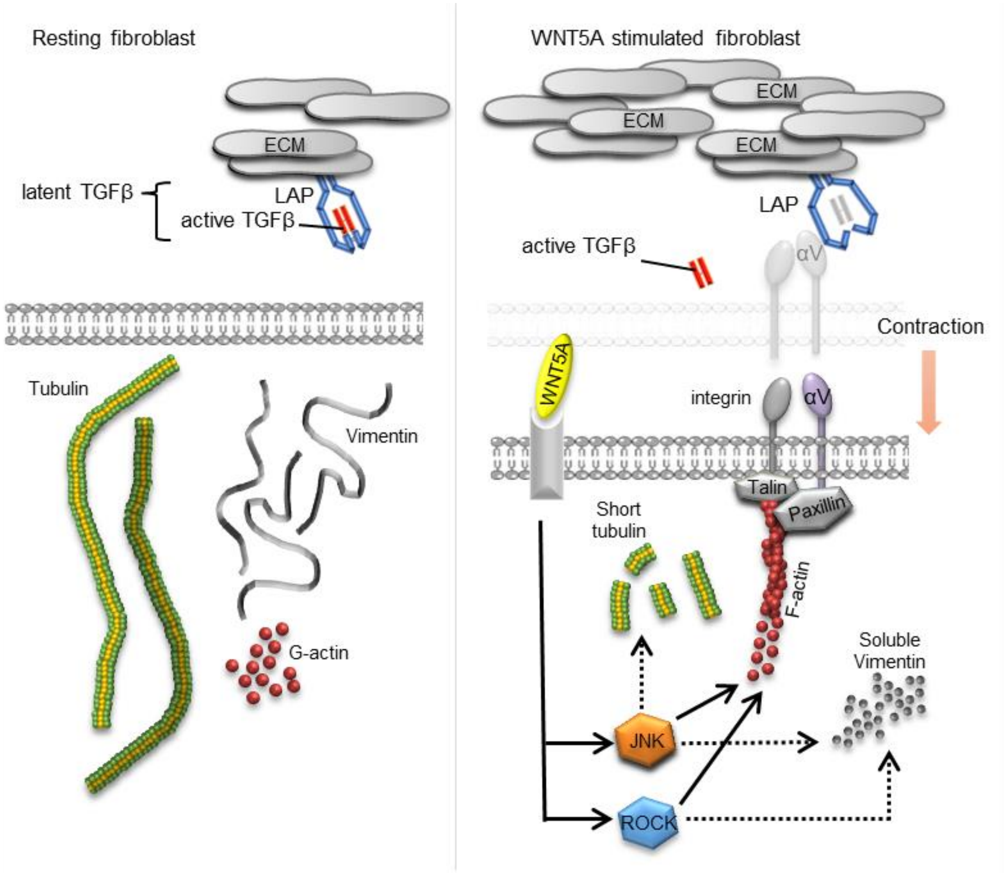

Supplementary Figure 17: Schematic illustration of the activation of latent TGFβ by WNT5A.

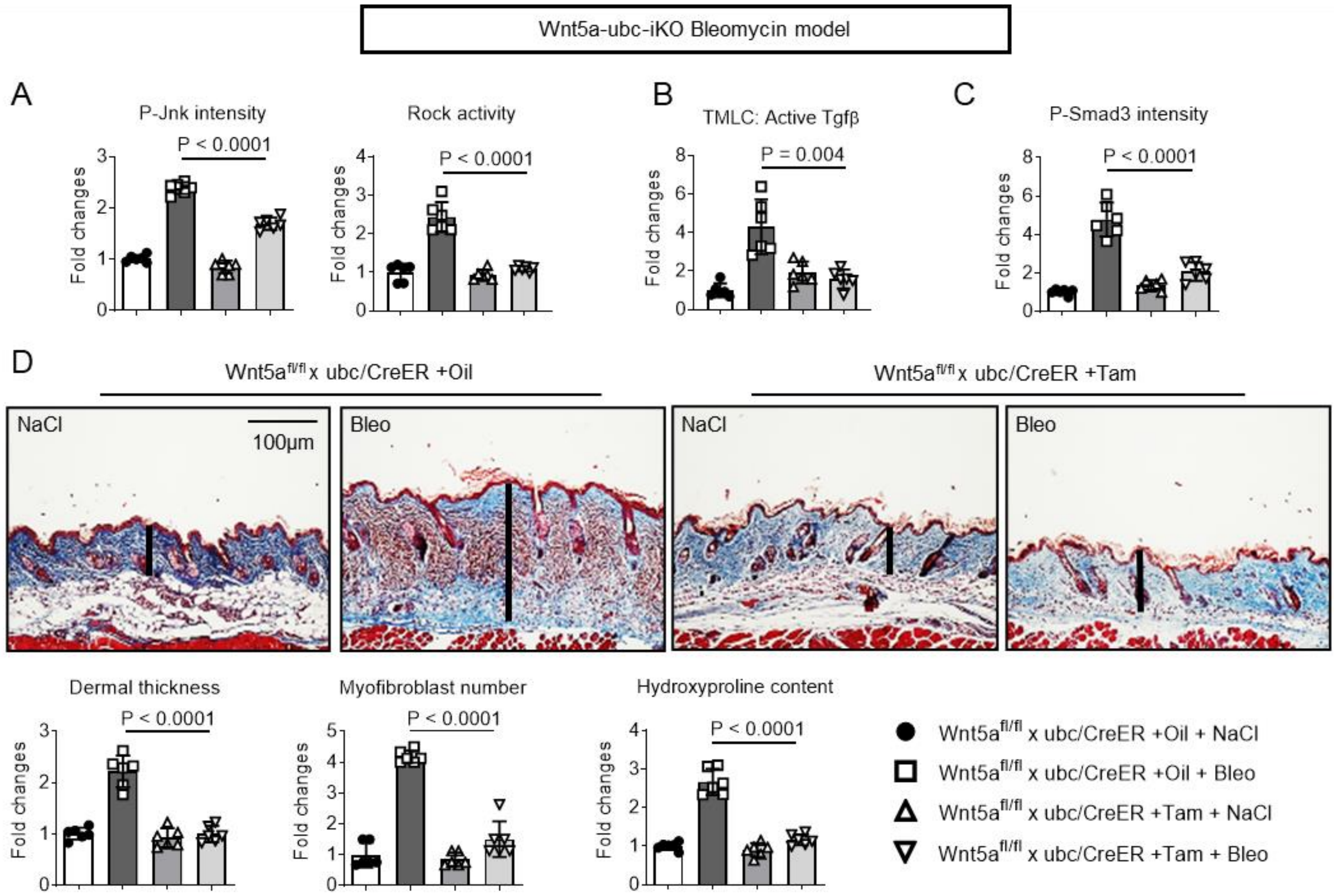

Supplementary Figure 18: Ubiquitous, inducible knockout of Wnt5a ameliorates bleomycin-induces skin fibrosis.

(**A**) Quantification of the IF staining for P-Jnk, and of Rock activity. (**B**) Level of active Tgfβ measured by TMLC. (**C**) Quantification of the IF staining for P-Smad3. (**D**) Representative Trichrome stainings and quantification of the dermal thickness, of myofibroblast counts, and of the hydroxyproline content (n = 6 each group). Results are shown as median ± IQR with data representing individual data points. The statistical significance was determined by one-way ANOVA with Tukey’s multiple comparison test. Bleo: Bleomycin. Tam: Tamoxifen. iKO: inducible knockout.

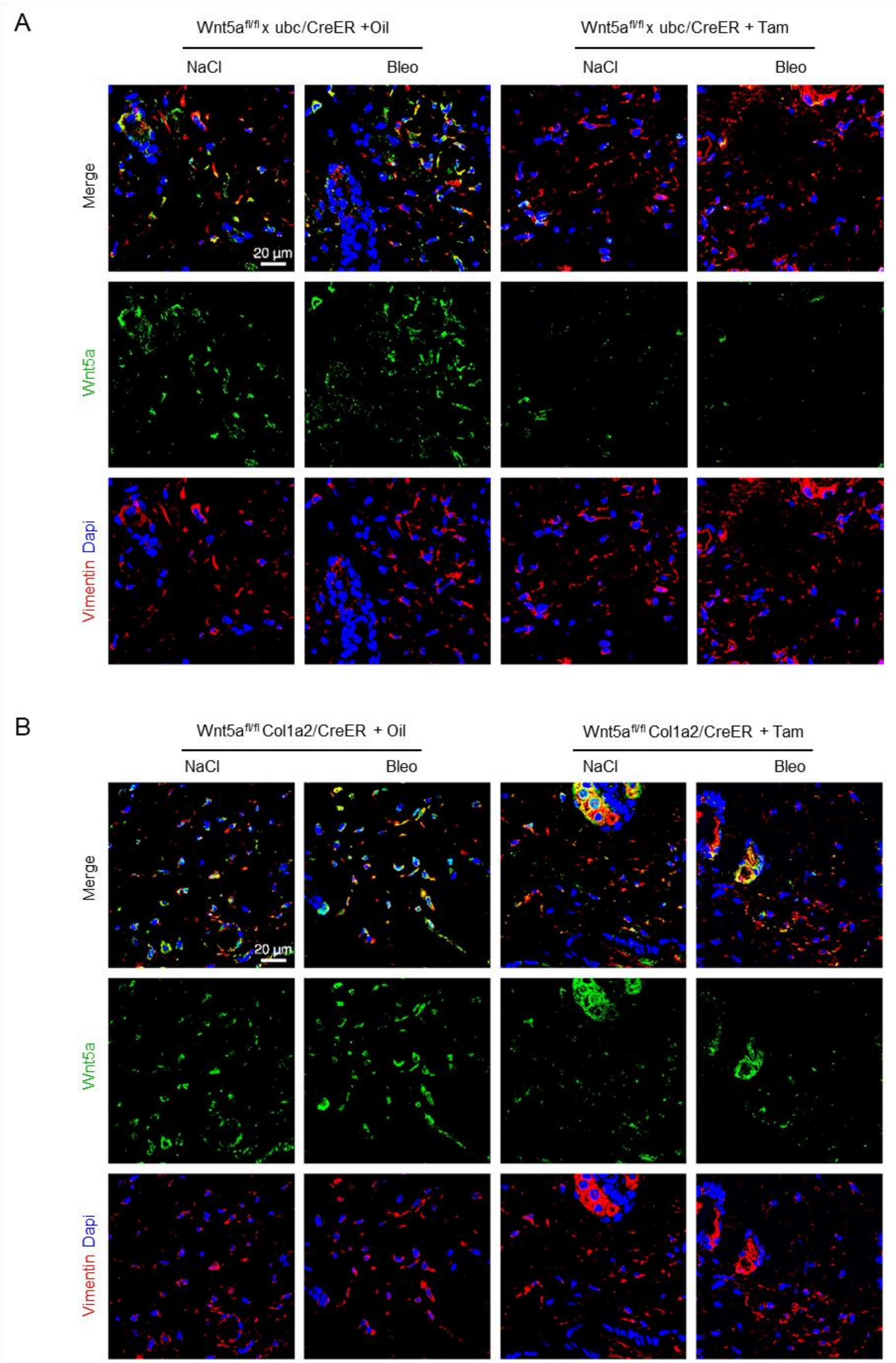

Supplementary Figure 19: Efficiency of ubiquitous or fibroblast-specific, inducible knockout of Wnt5a.

(**A**) Representative confocal images of Wnt5a expression in the skin of mice with ubiquitous knockout of Wnt5a (n = 6 each group). (**B**) Representative confocal images of Wnt5a expression in mice with fibroblast specific knockout of Wnt5a (n = 6 each group). Bleo: Bleomycin. Tam: Tamoxifen. iKO: inducible knockout.

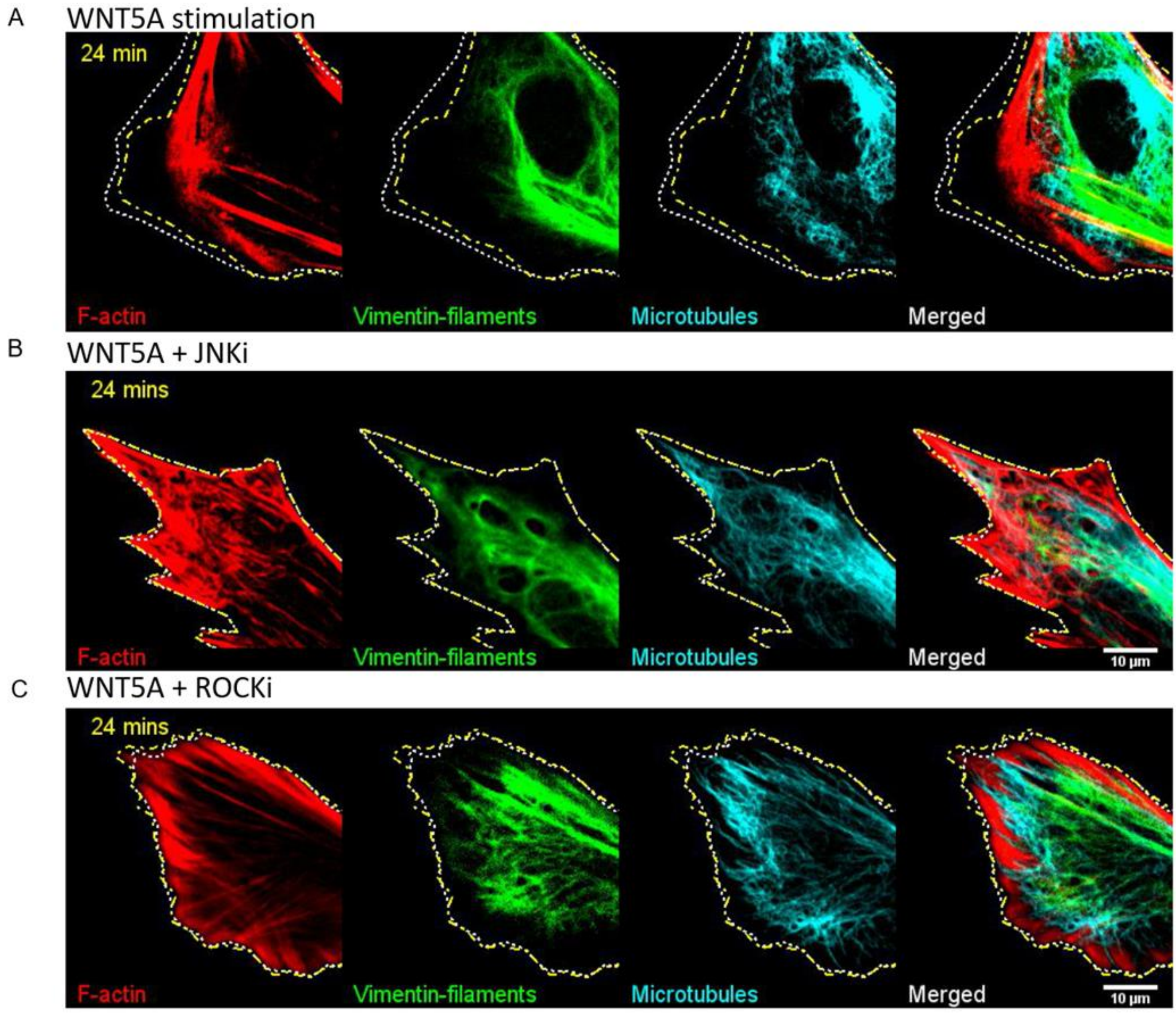

Supplementary video 1: Dynamic changes in F-actin, Vimentin filaments and microtubules in fibroblasts stimulated with WNT5A with or without JNK or ROCK inhibition.

(**A**) Fibroblasts stimulated with WNT5A. Representative example of live cell imaging of human dermal fibroblasts before and up to 90 mins after stimulation with recombinant WNT5A using a spinning disk microscope. WNT5A induces cellular contraction with formation of stress fibers (red) at the contractile pole and parallel retraction of vimentin filaments (green) and degradation of the tubulin network (blue) at these sites. The cell membrane at the beginning of the observation periods are indicated by white dotted lines and the movement of the cellular membrane is indicated by yellow dotted lines. (**B**) Fibroblasts pretreated with JNKi before WNT5A stimulation. (**C**) Fibroblasts pretreated with ROCKi before WNT5A stimulation. JNKi: JNK inhibitor. ROCKi: ROCK inhibitor.
