# Supplementary material for: Noncanonical WNT5A controls the activation of latent TGF-β to drive fibroblast activation and tissue fibrosis
Source: J Clin Invest. 2024 Mar 26;134(10):e159884. doi: 10.1172/JCI159884 (PMC11093613; doi:10.1172/JCI159884)

Full unedited gel for Figure 1F

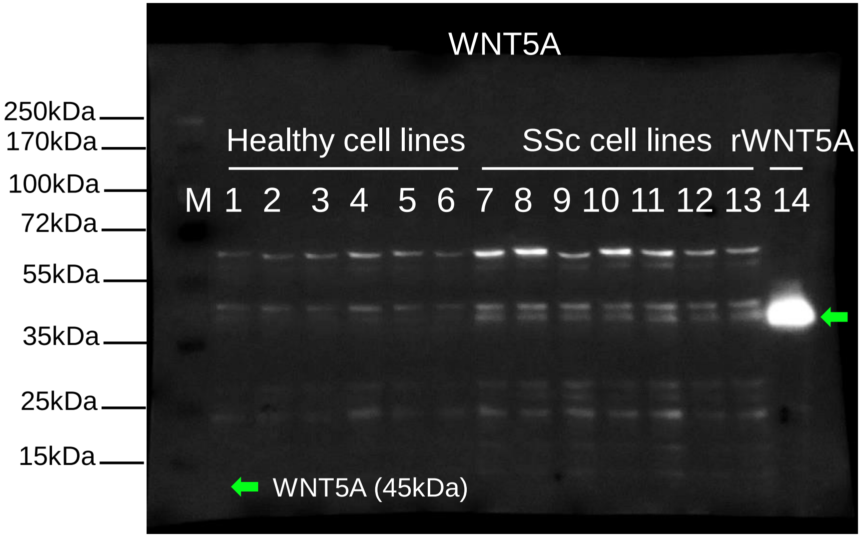

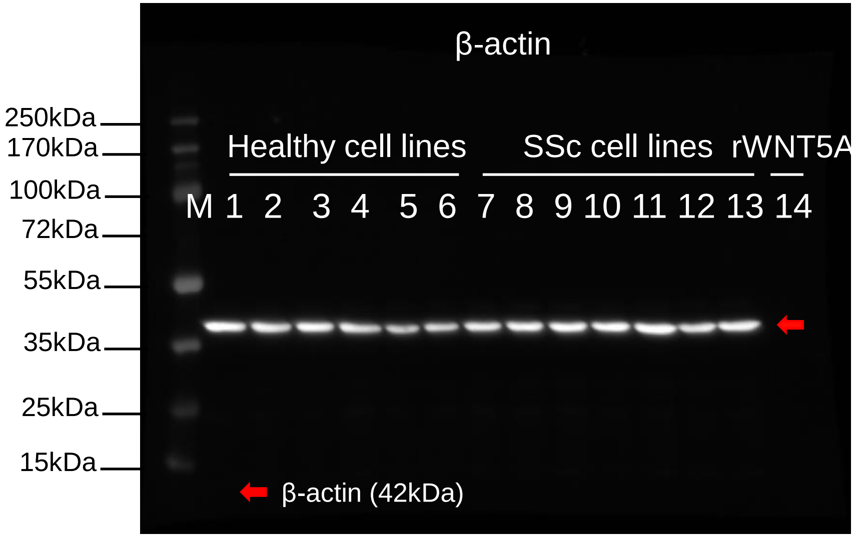

WNT5A - inversed

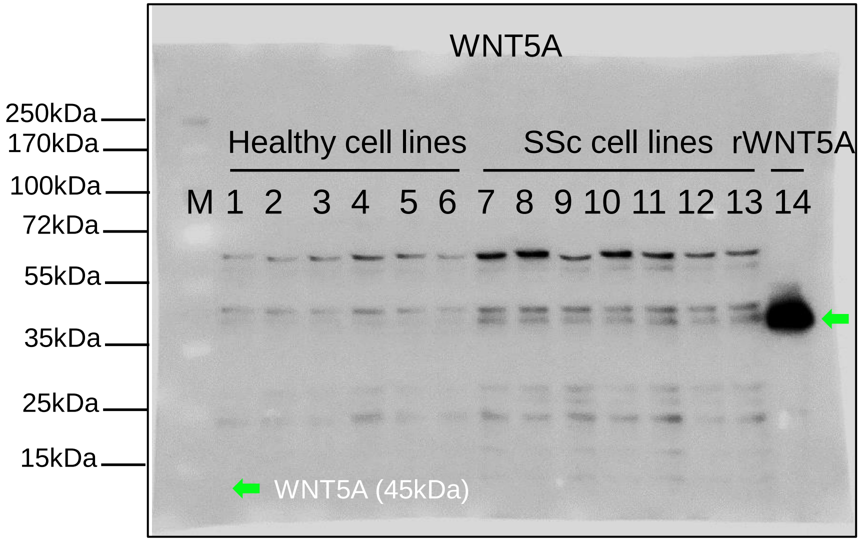

β-actin - inversed

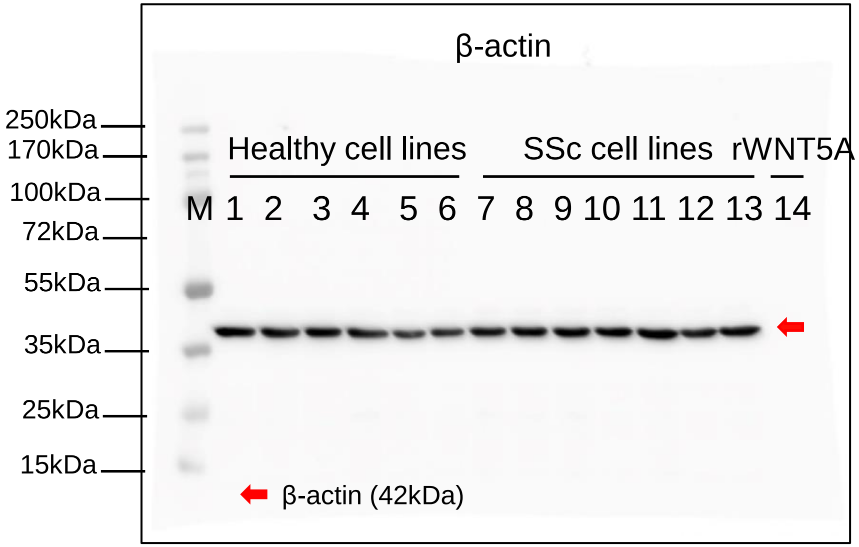

Full unedited gel for Supplementary Figure 2C

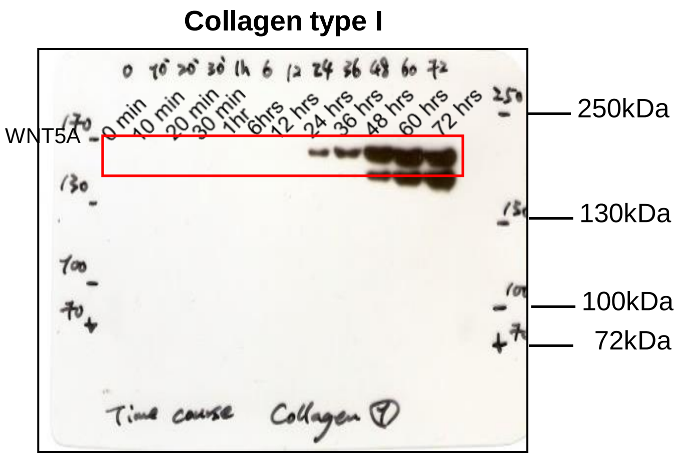

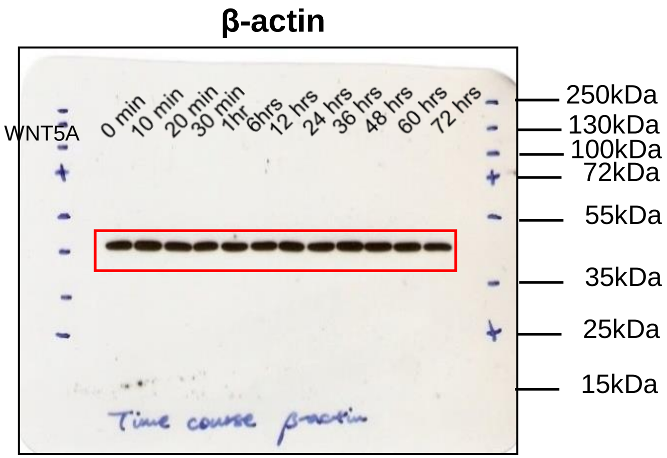

Full unedited gel for Supplementary Figure 2G

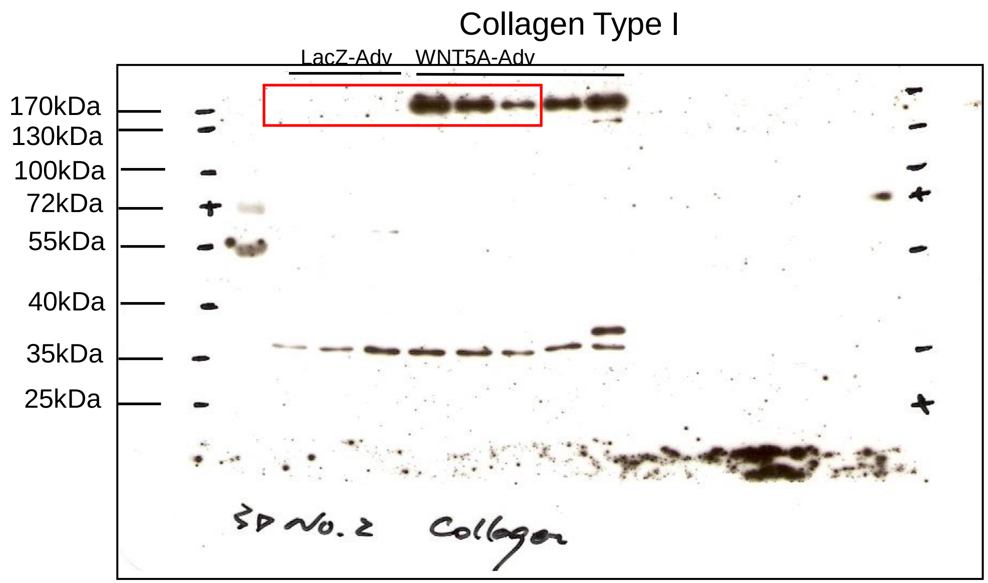

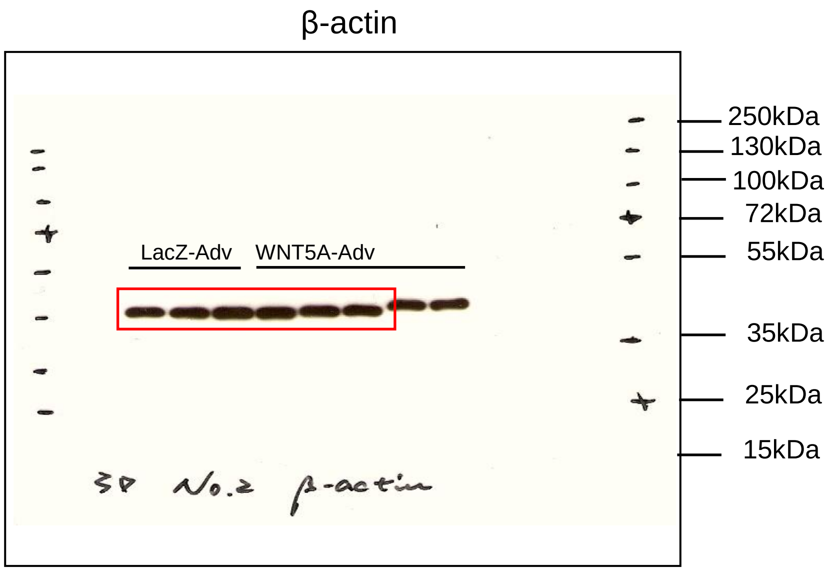

Full unedited gel for Supplementary Figure 4A

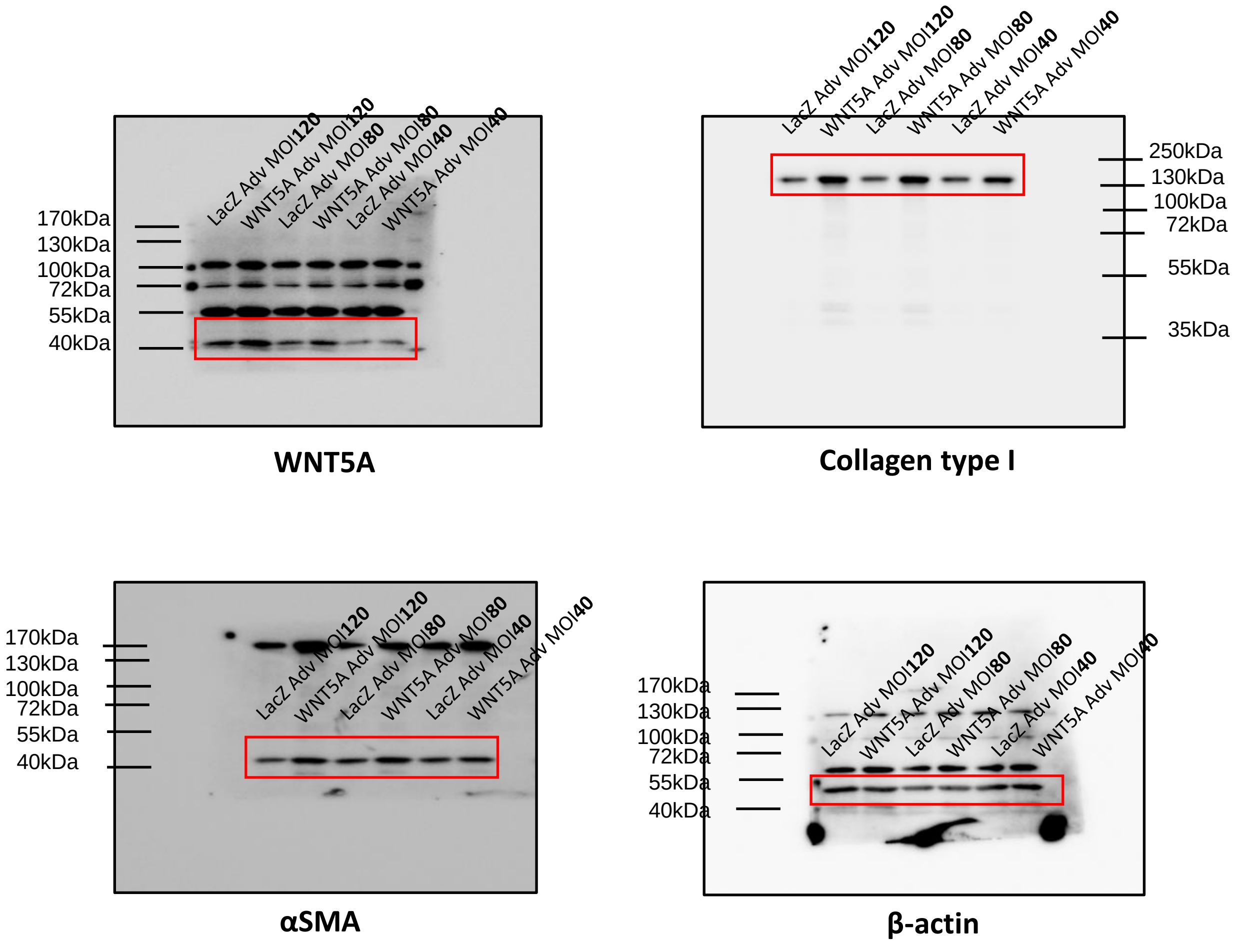

Full unedited gel for Figure 3A

P-JNK

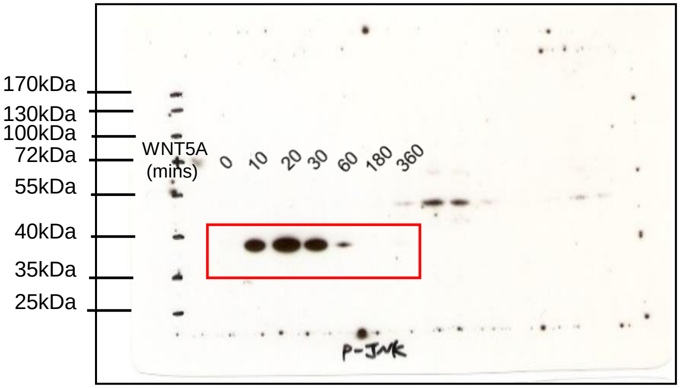

JNK

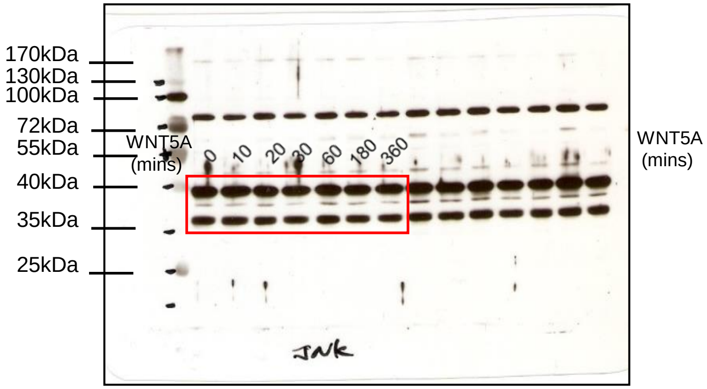

β-actin

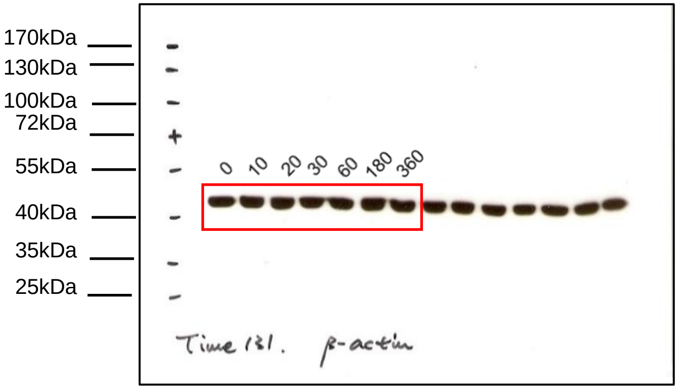

Full unedited gel for Supplementary Figure 5A

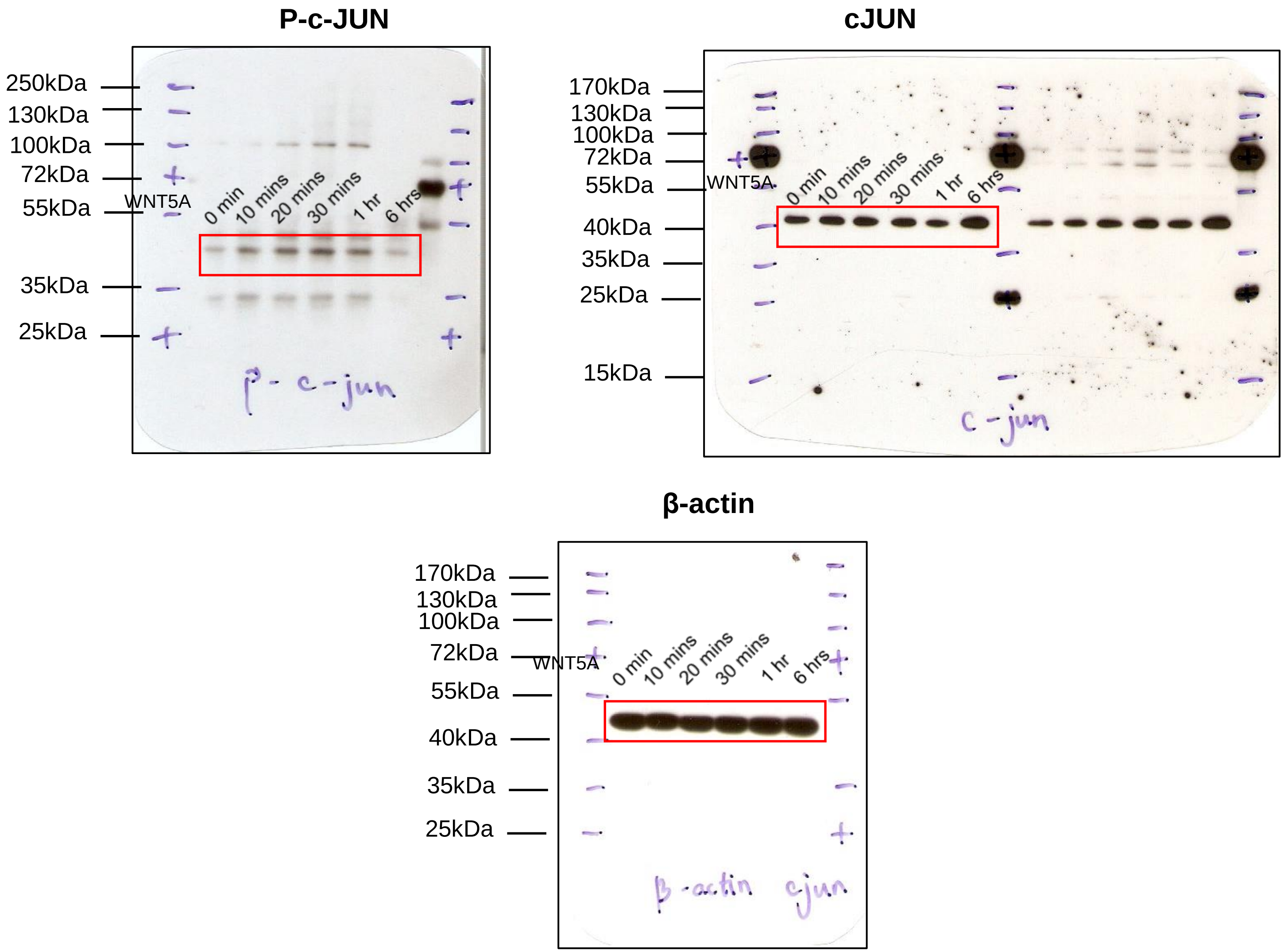

Full unedited gel for Supplementary Figure 8A

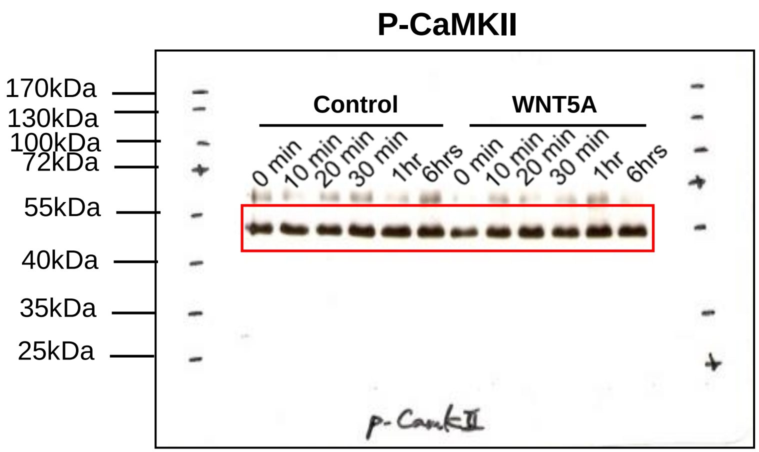

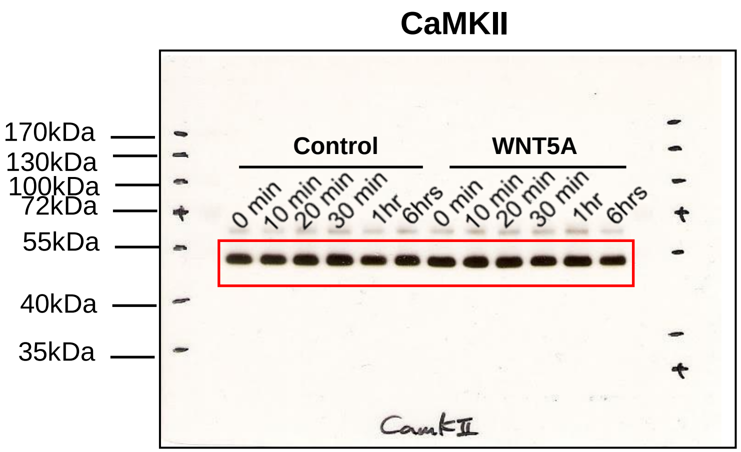

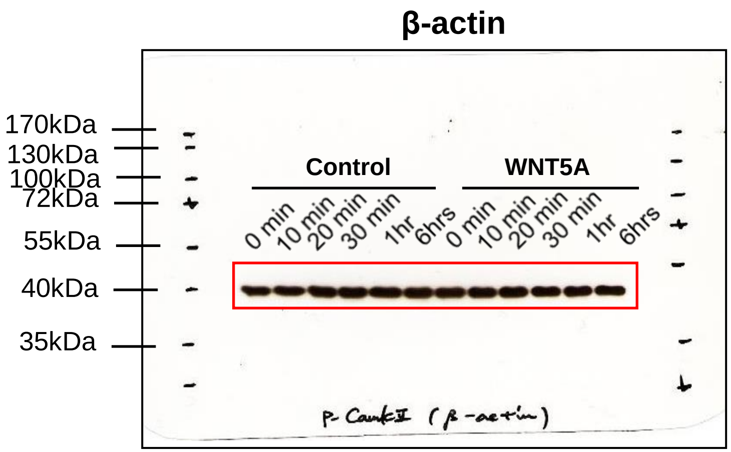

Full unedited gel for Supplementary Figure 8F

β-catenin

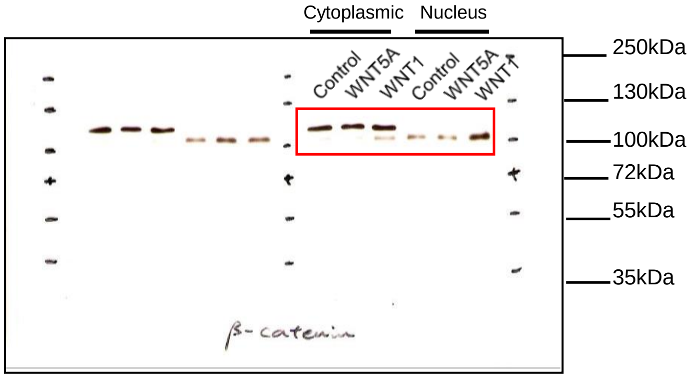

Lamin A/C

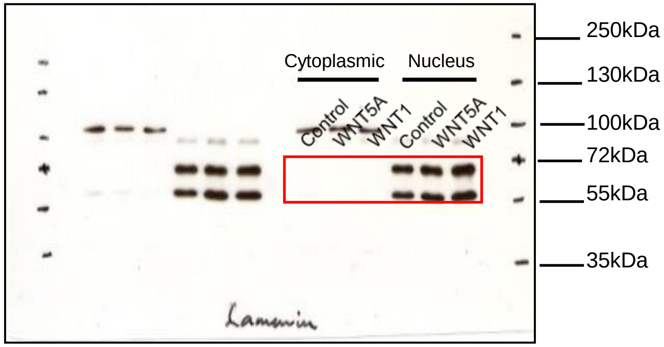

β-actin

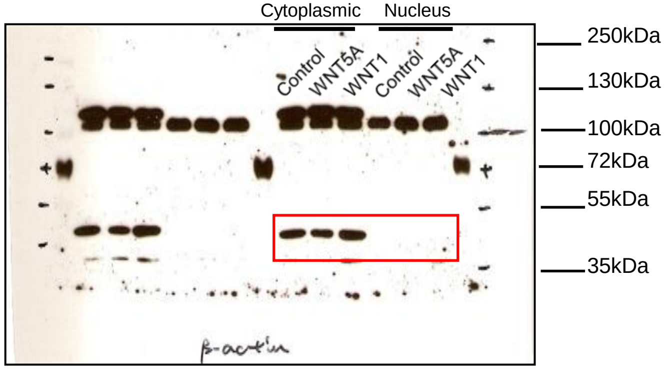

Full unedited gel for Figure 4A

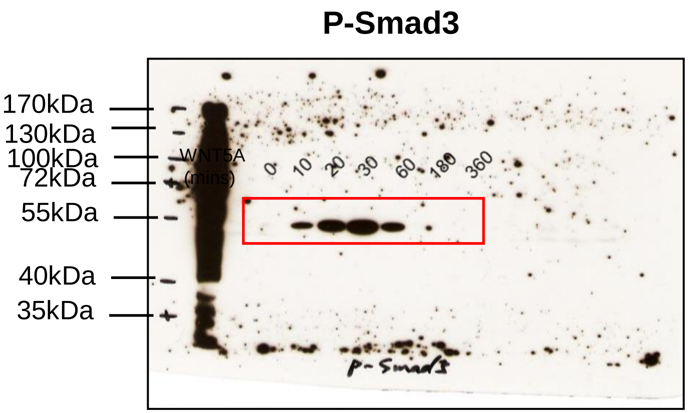

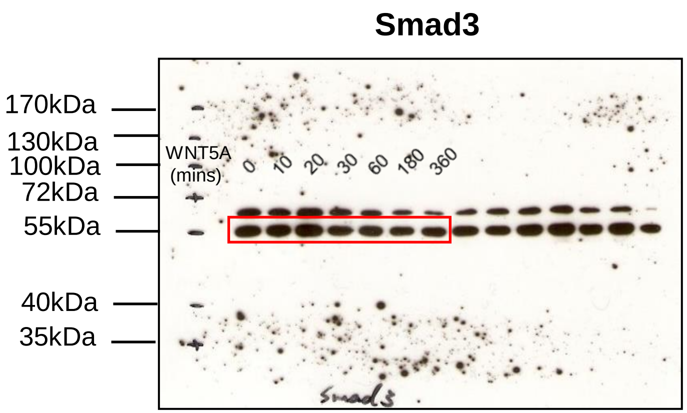

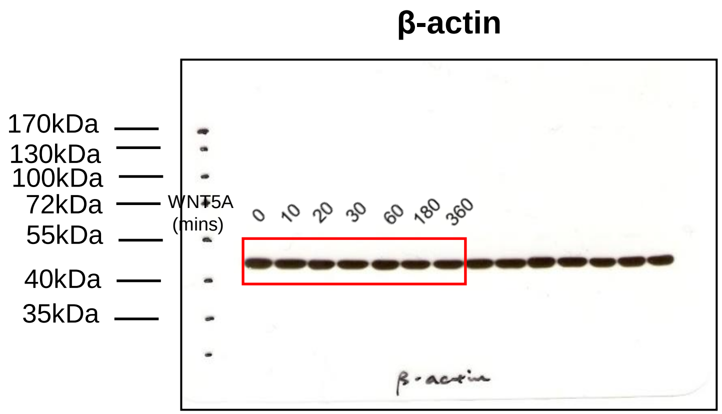

Full unedited gel for Supplementary Figure 9D

P-SMAD3

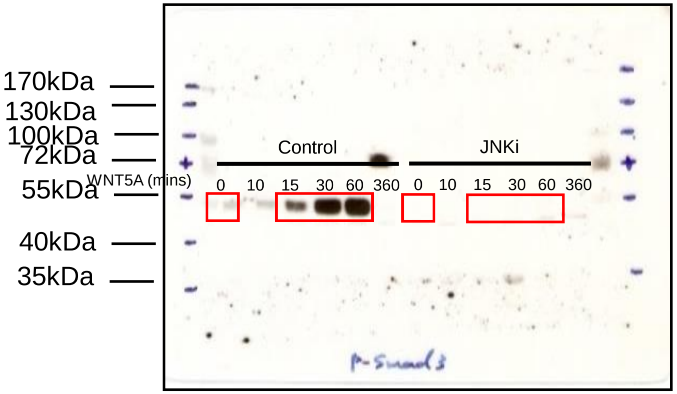

SMAD3

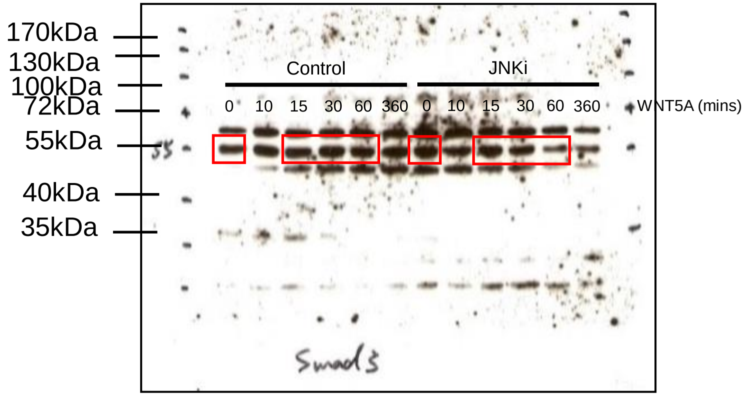

β-actin

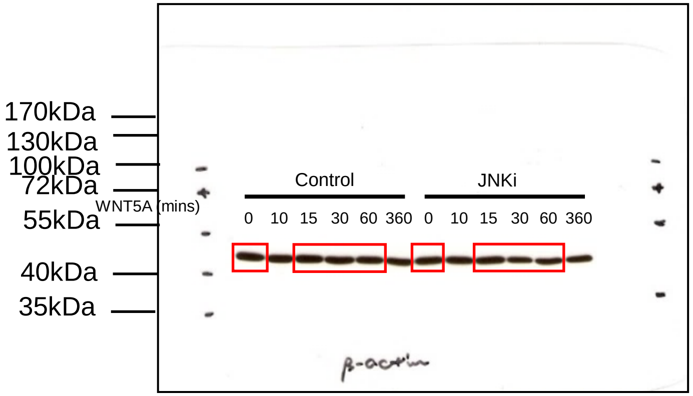

Full unedited gel for Supplementary Figure 9G

P-SMAD3

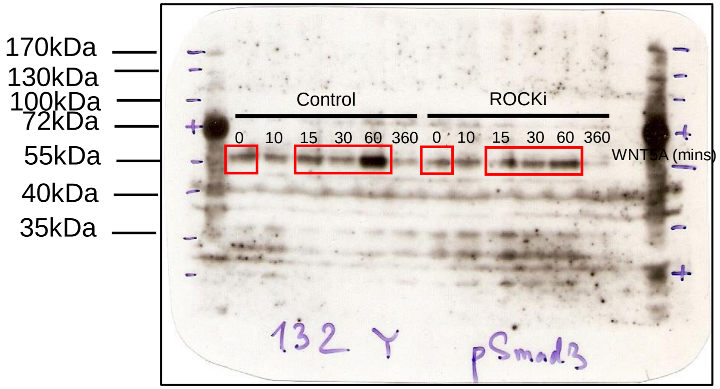

SMAD3

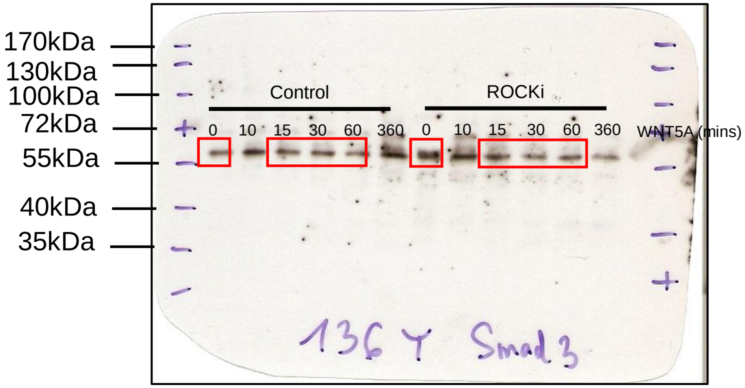

β-actin

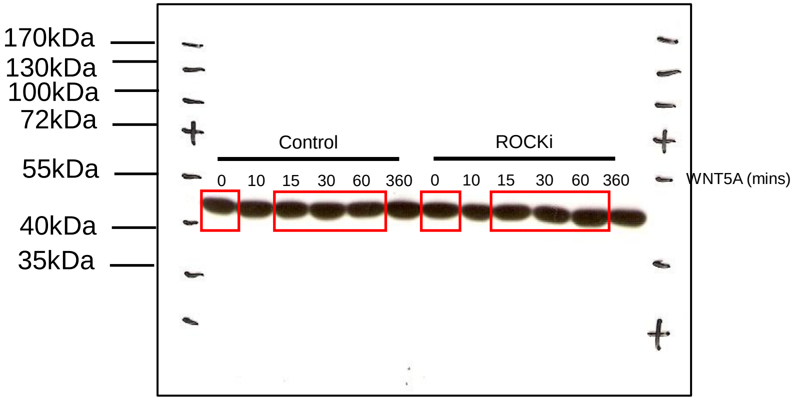

Full unedited gel for Supplementary Figure 10A

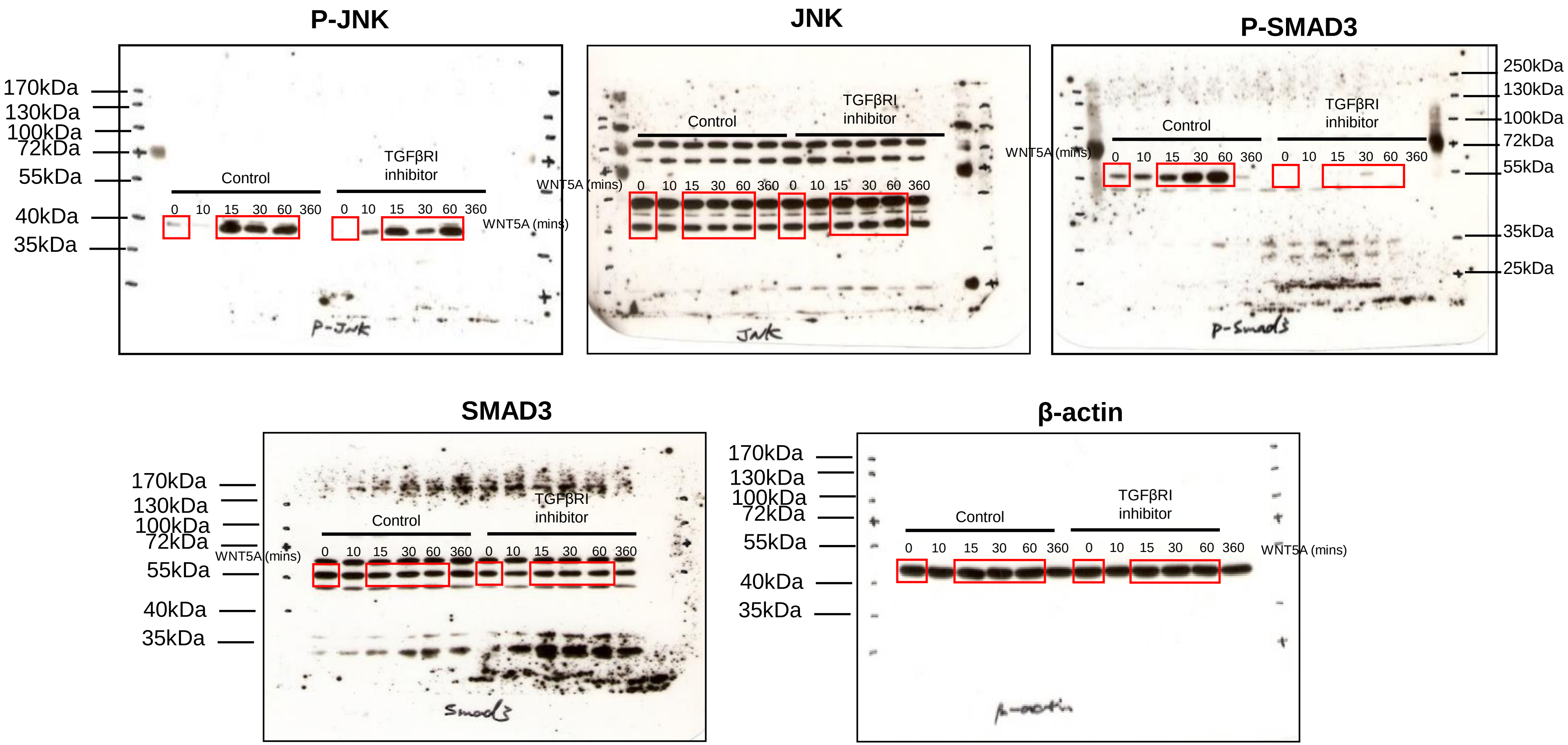

Full unedited gel for Supplementary Figure 10B

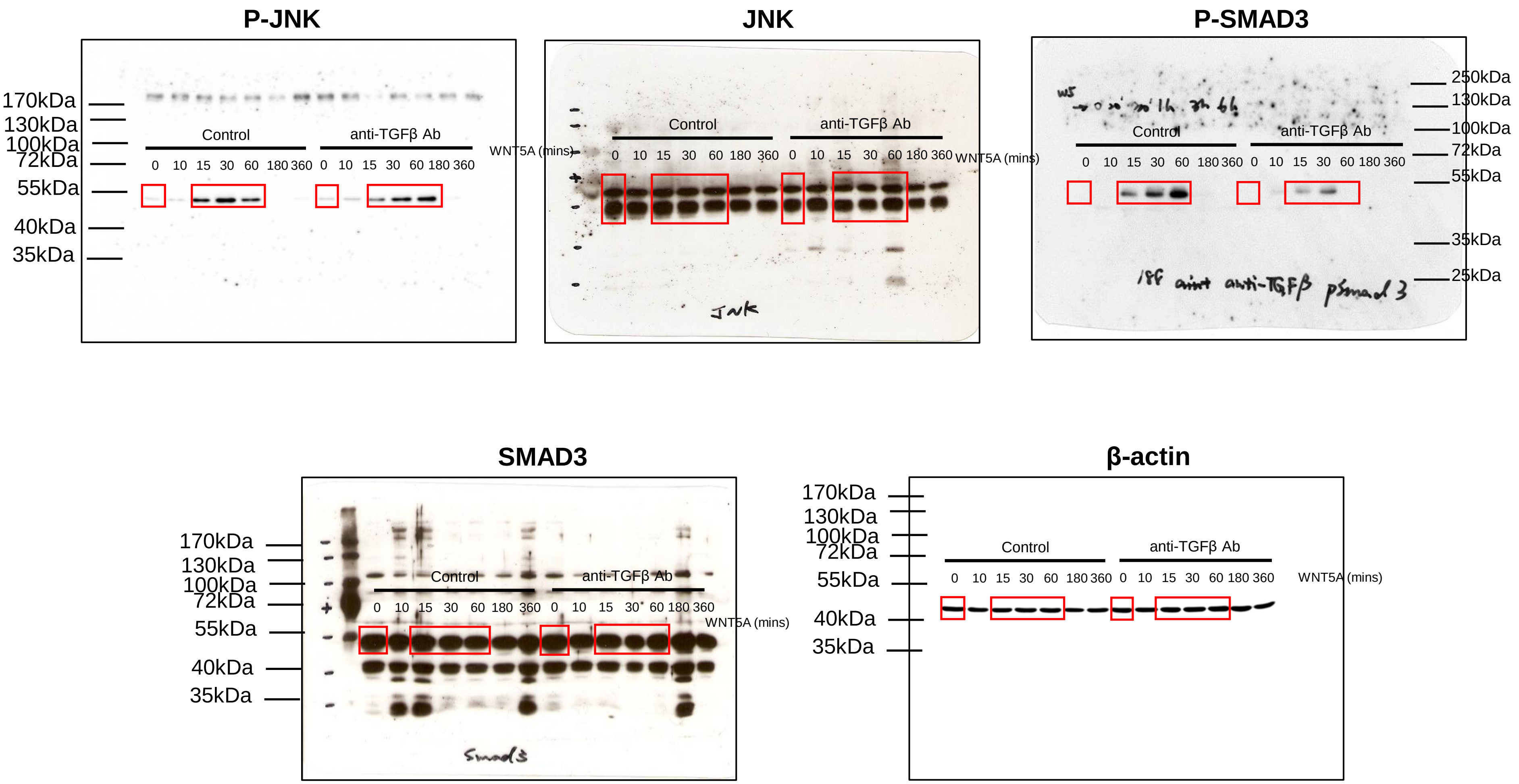

Full unedited gel for Figure 5B

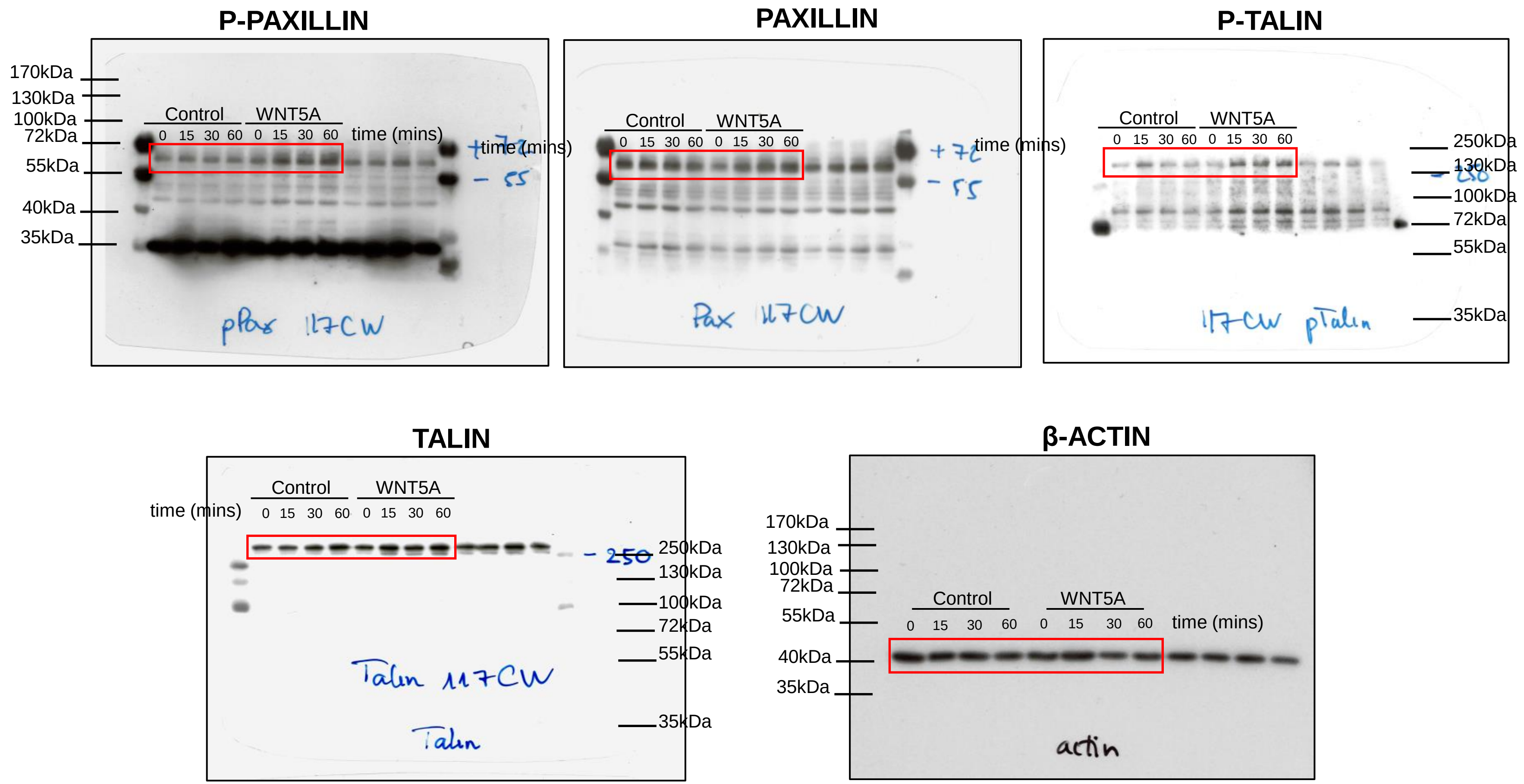

Full unedited gel for Figure 5G

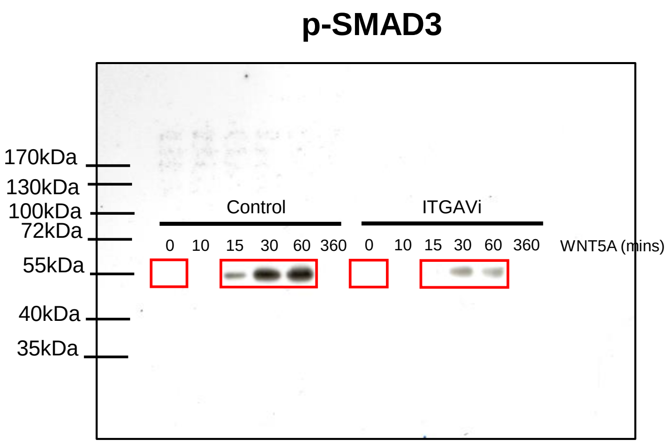

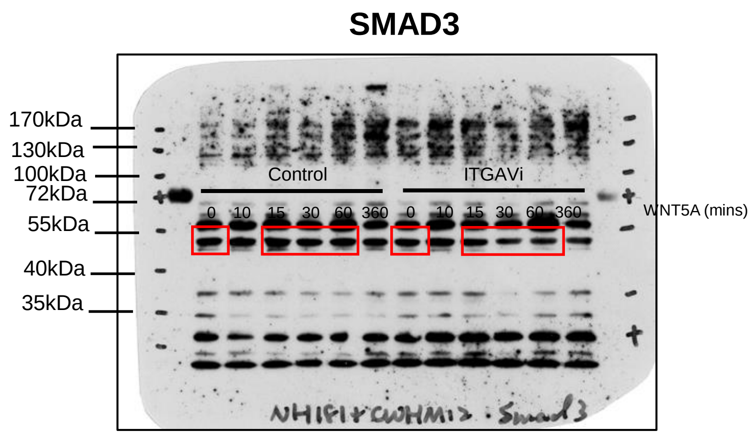

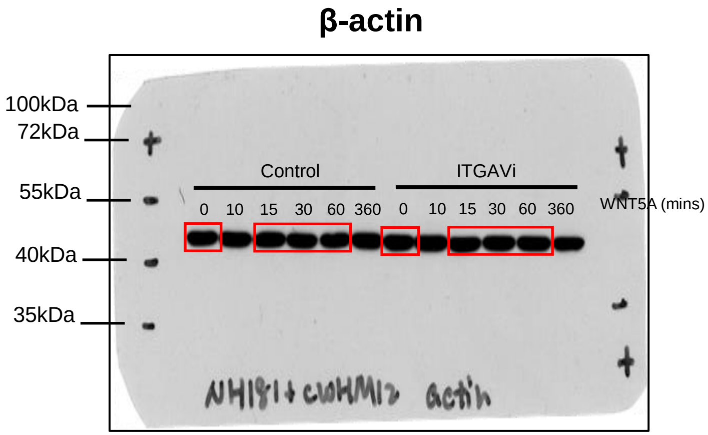

Full unedited gel for Supplementary Figure 12G

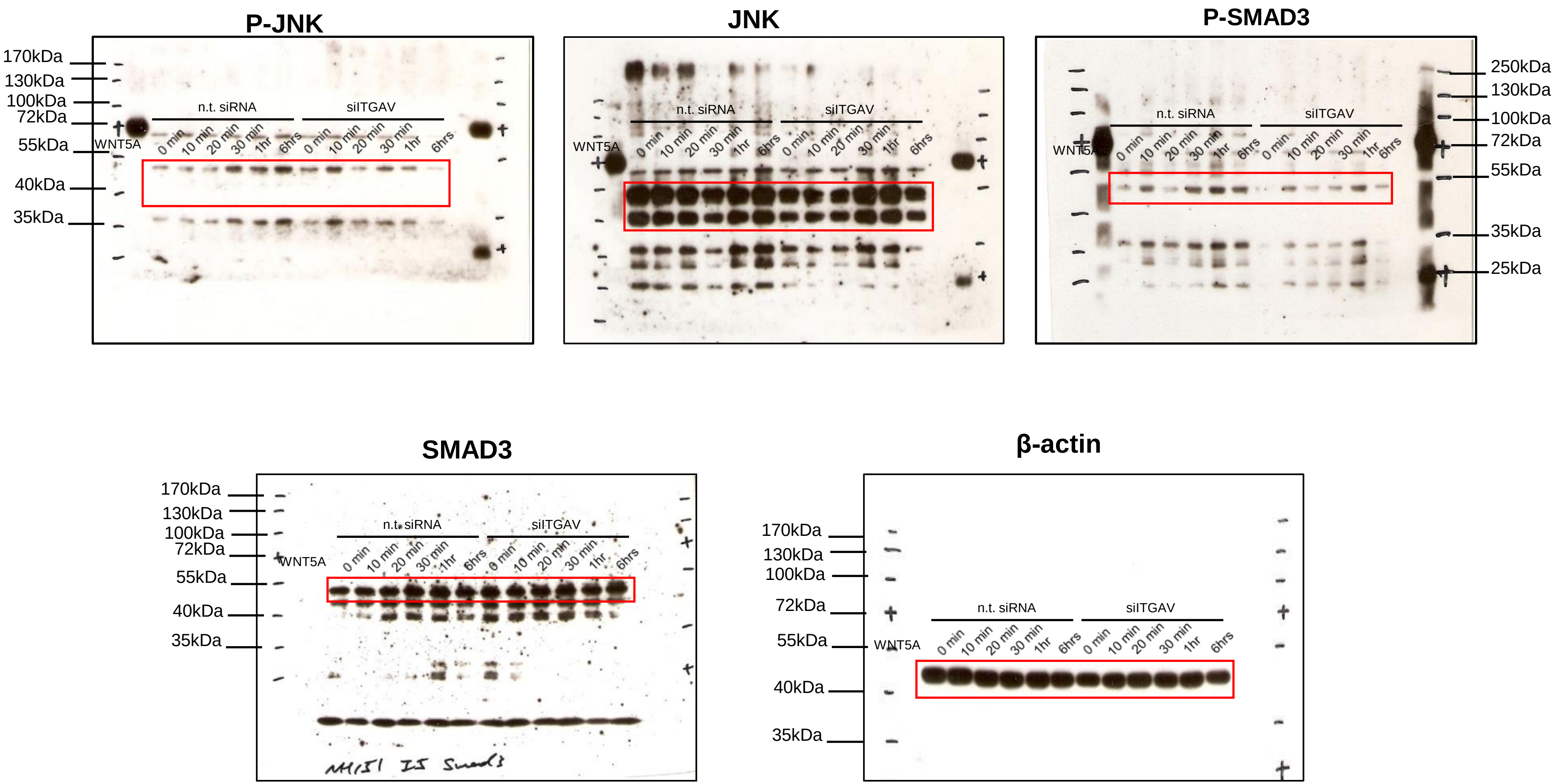

Full unedited gel for Figure 6E

p-SMAD3

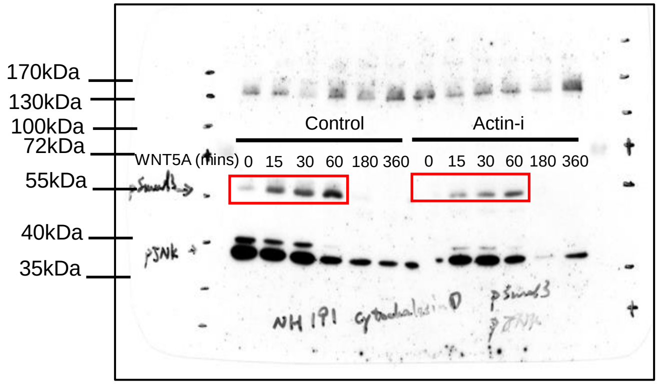

SMAD3

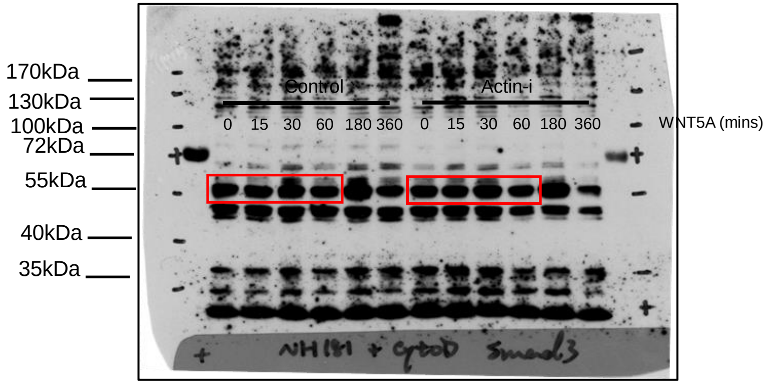

β-actin

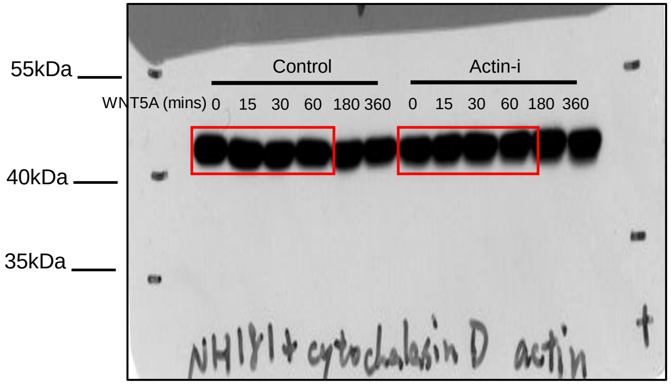

Full unedited gel for Supplementary Figure 14A

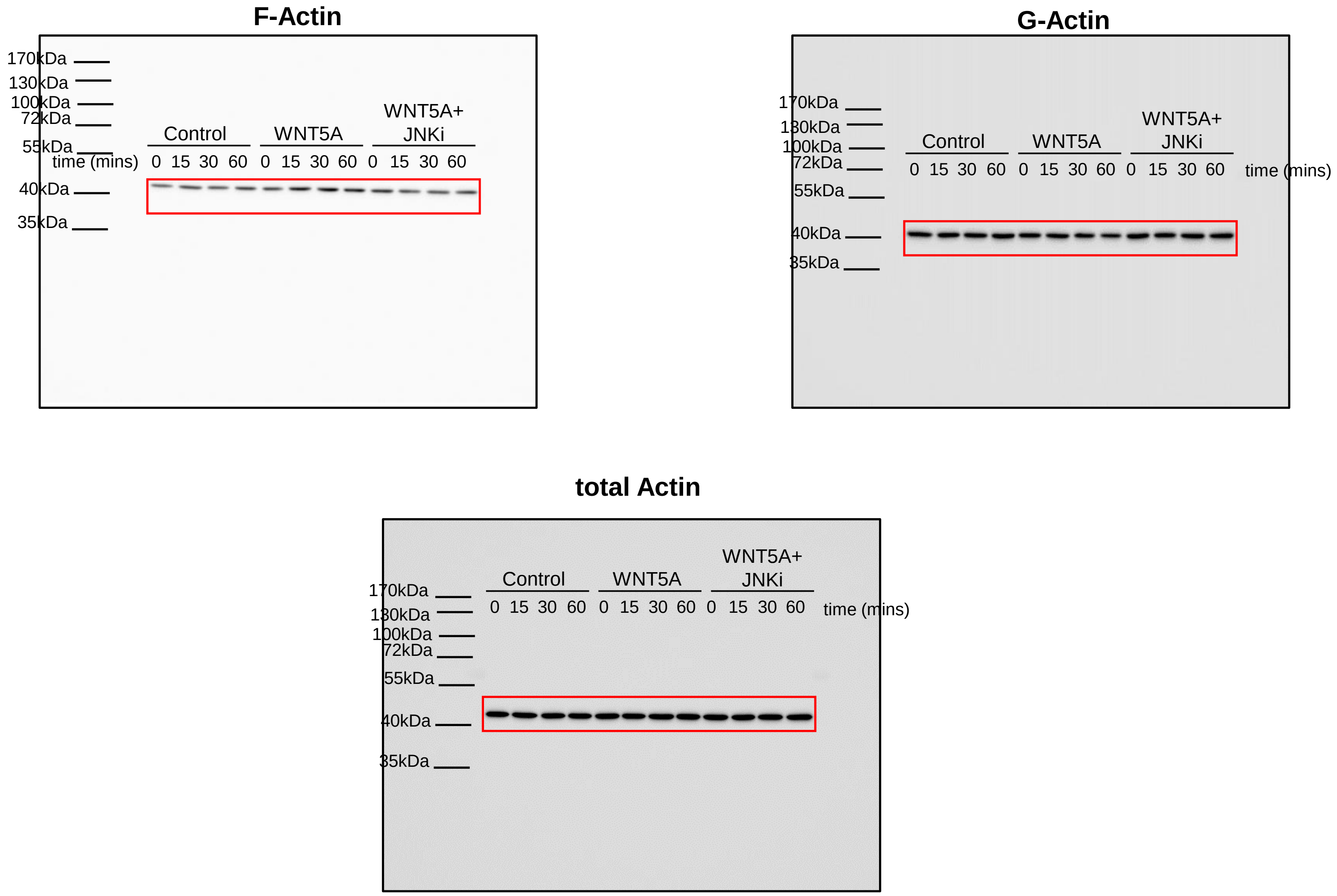

Full unedited gel for Supplementary Figure 14B

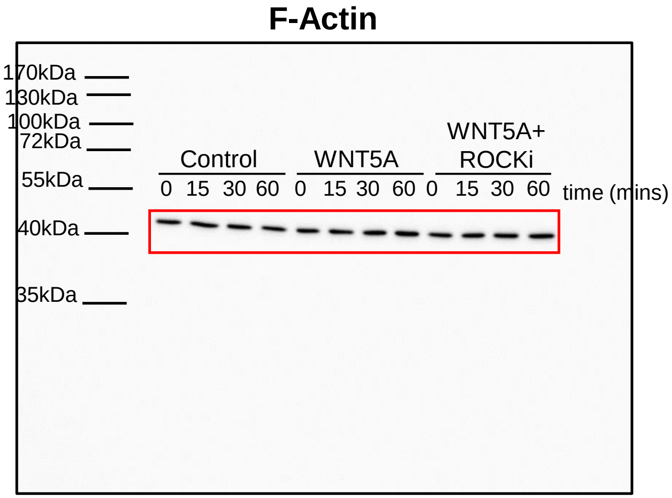

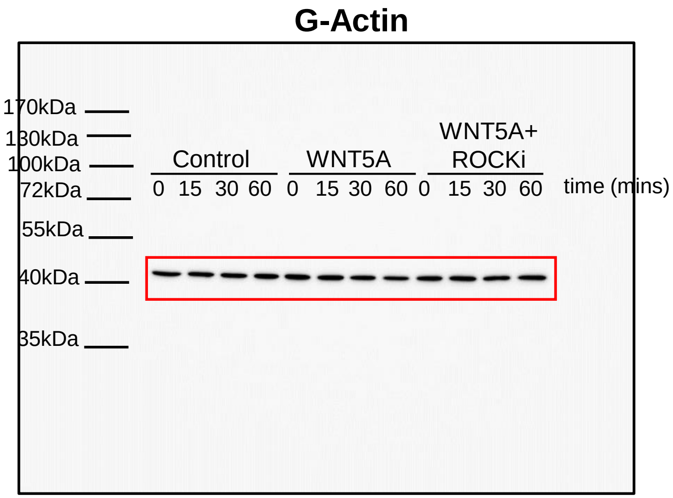

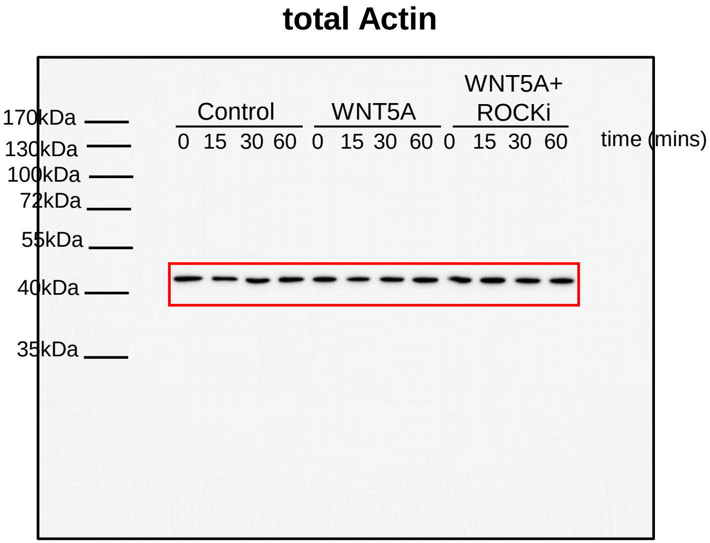

Full unedited gel for Supplementary Figure 14C

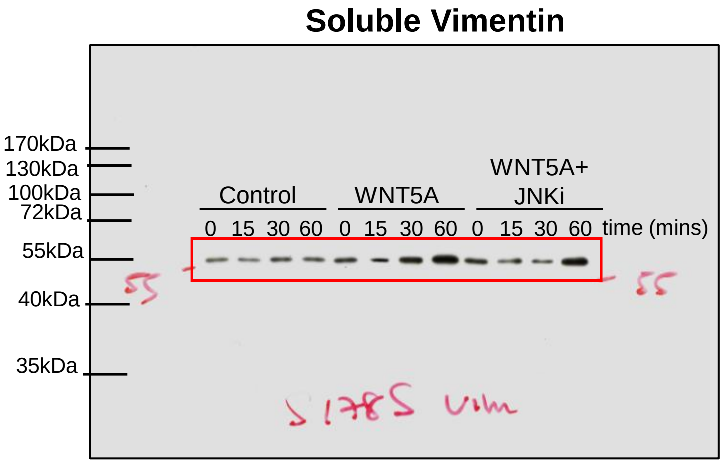

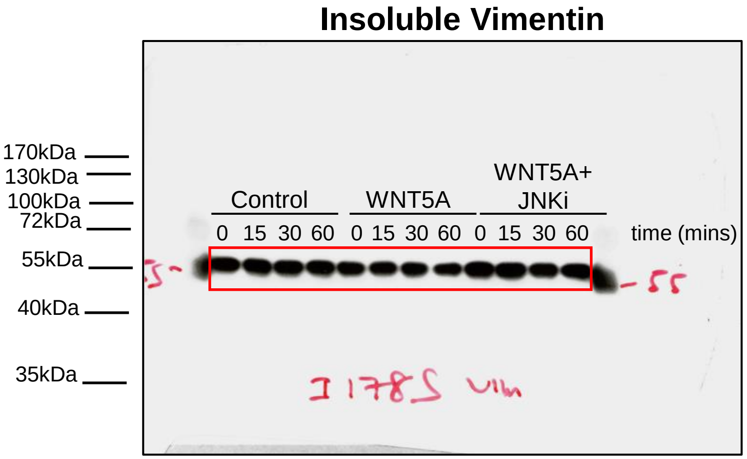

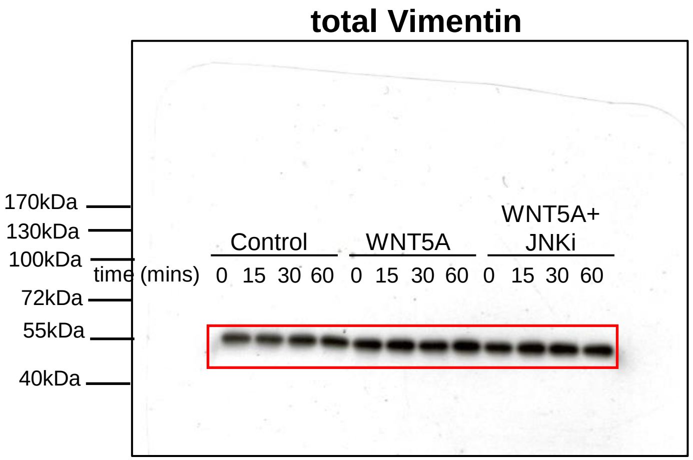

Full unedited gel for Supplementary Figure 14D

Soluble Vimentin

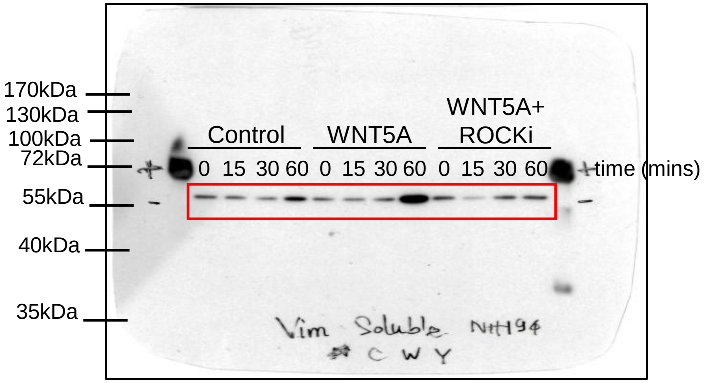

Insoluble Vimentin

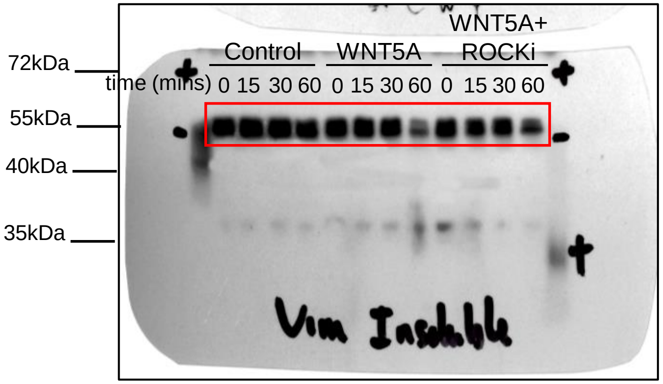

total Vimentin

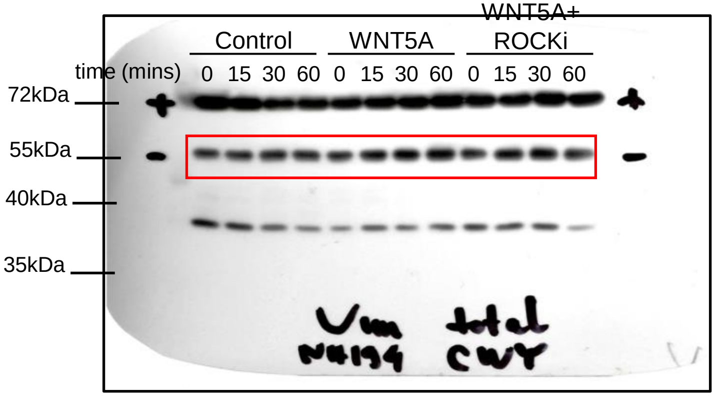

Full unedited gel for Supplementary Figure 14E

Short Tubulin

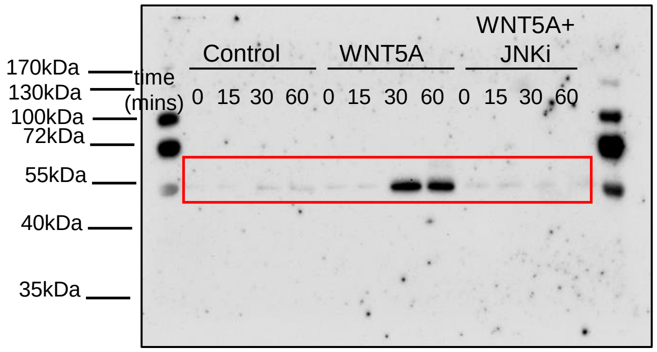

Long Tubulin

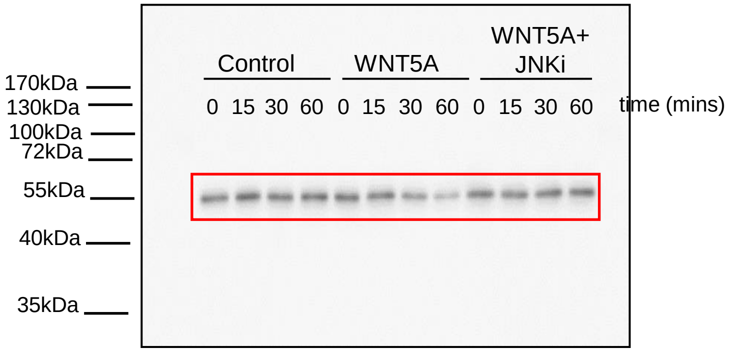

Soluble Tubulin

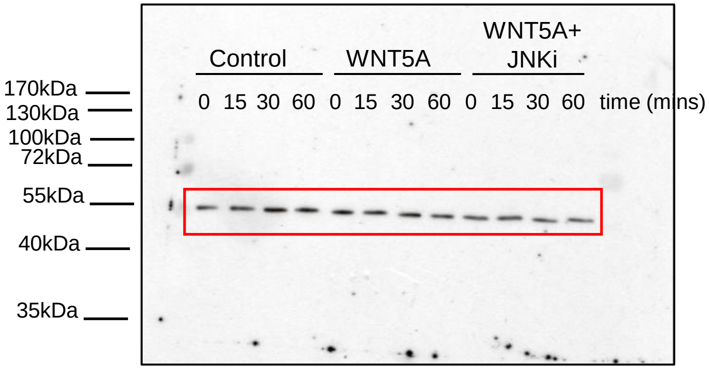

total Tubulin

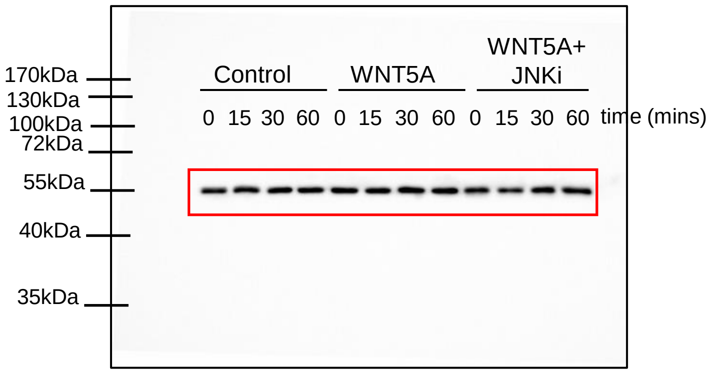

Full unedited gel for Supplementary Figure 10F

Short Tubulin

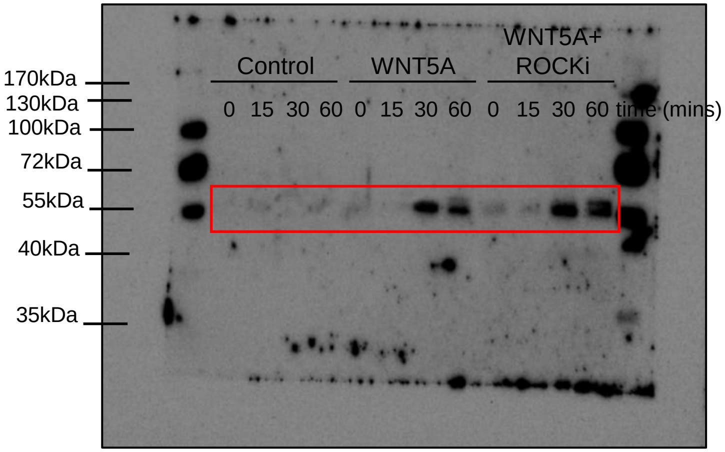

Long Tubulin

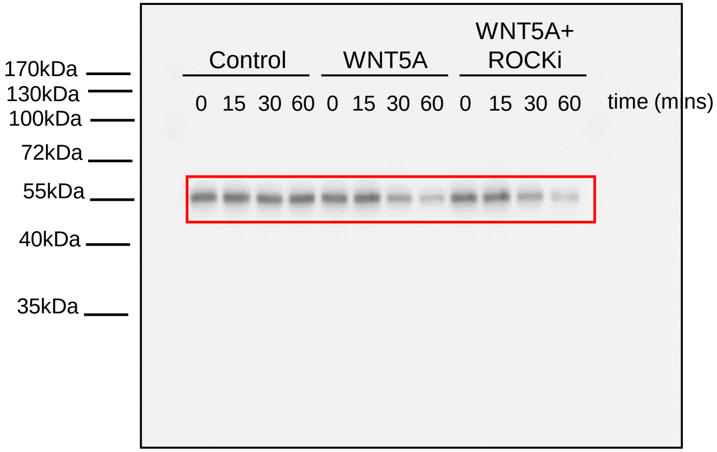

Soluble Tubulin

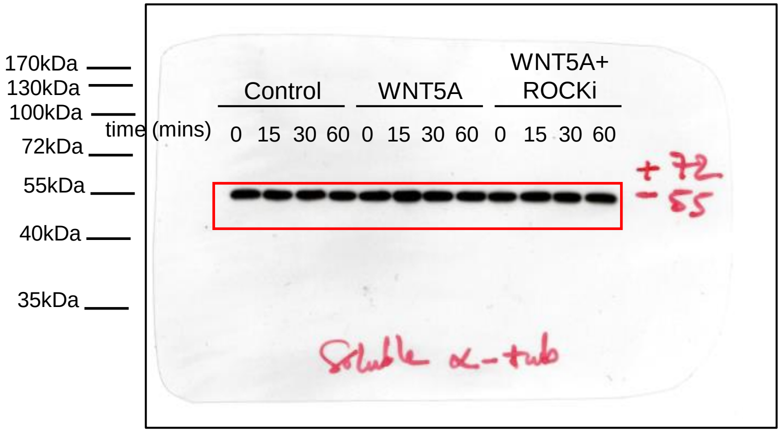

total Tubulin

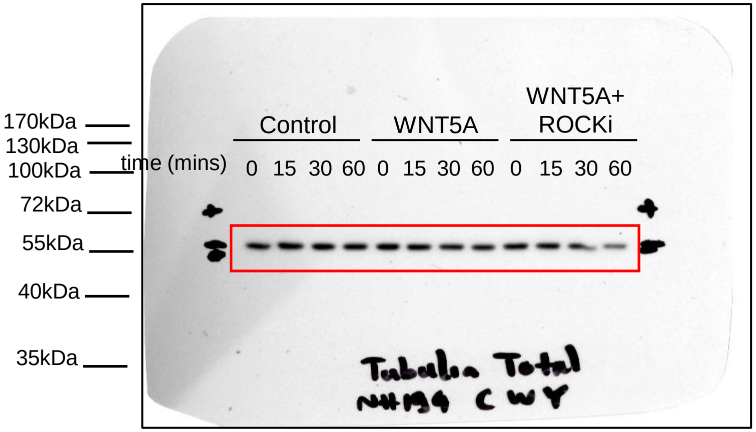

Full unedited gel for Supplementary Figure 15A

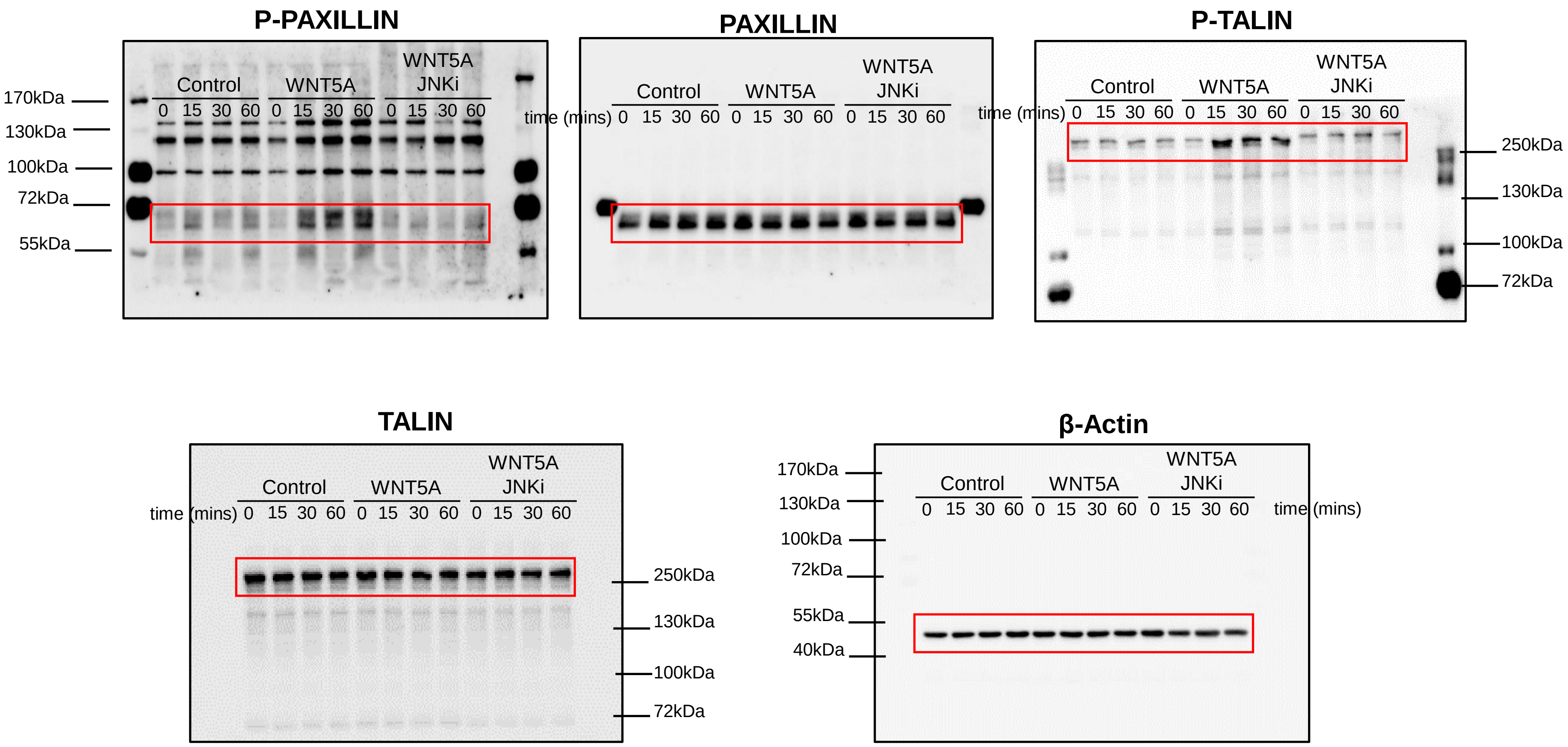

Full unedited gel for Supplementary Figure 15B

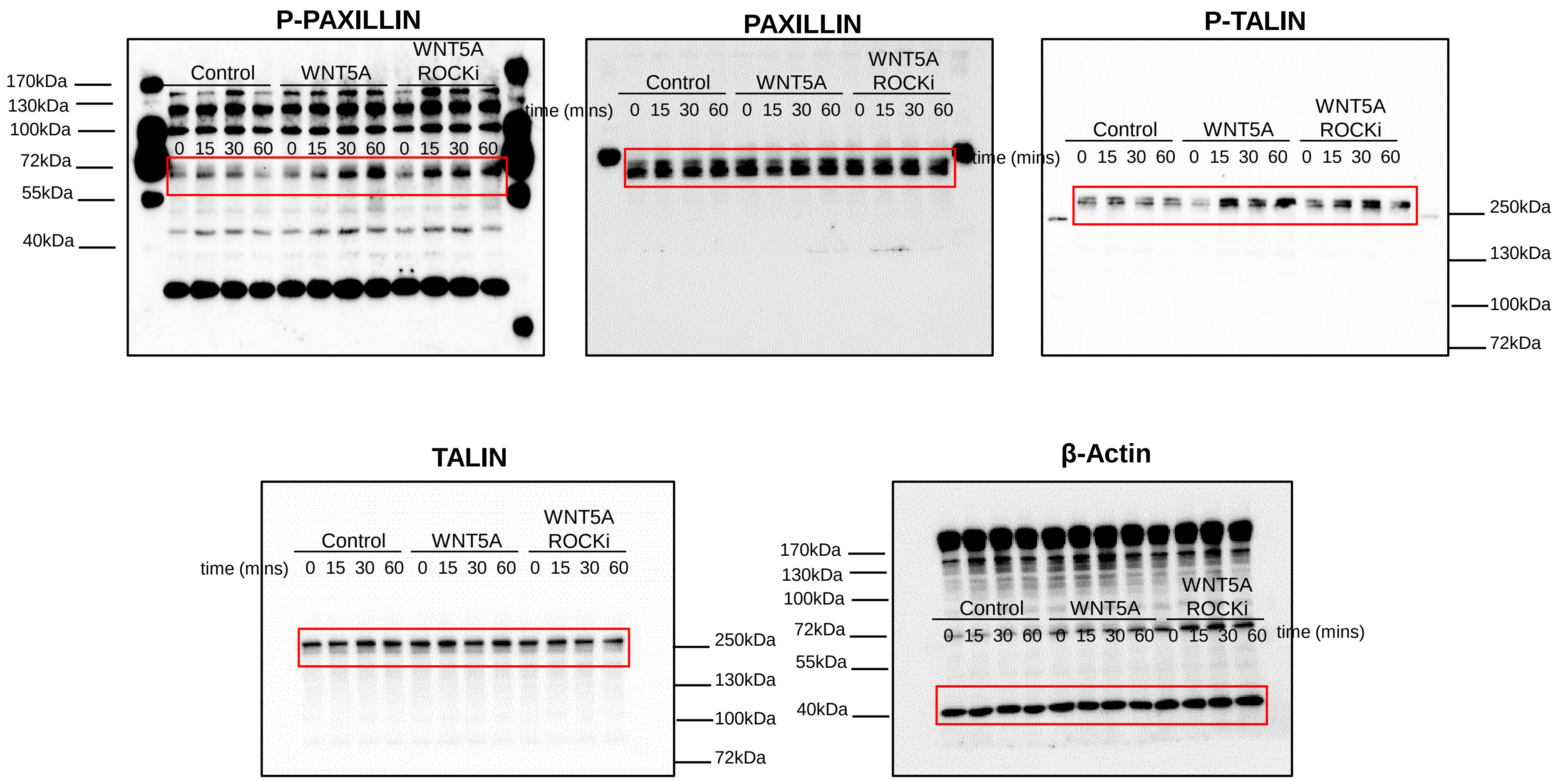

Supplement: Unedited blot and gel images [file jci-134-159884-s037.pdf]
